# Supplementary material for: Cu(I) Complexes of Multidentate N,C,N- and P,C,P-Carbodiphosphorane Ligands and Their Photoluminescence
Source: Molecules. 2020 Sep 1;25(17):3990. doi: 10.3390/molecules25173990 (PMC7504792; doi:10.3390/molecules25173990)
Supplement: Supplementary file 1 [file molecules-25-03990-s001.pdf]

Supporting Information

Cu(I) Complexes of Multidentate *N,C,N*- and *P,C,P*-Carbodiphosphorane Ligands and their Photoluminescence

Marius Klein[a], *Nemrud Demirel, Alexander Schinabeck*[b], *Hartmut Yersin*[b], *Jörg Sundermeyer**[a]

[a] Fachbereich Chemie and Wissenschaftliches Zentrum für Materialwissenschaften, Philipps-Universität Marburg, Hans-Meerwein-Straße 4, 35043 Marburg, Germany

[b] Institute for Physical Chemistry, University of Regensburg, 93040 Regensburg, Germany

Table of Contents

Experimental Procedures 3

Synthesis 3

- [(CuCl)$_2$(CDP(Py)$_2$)] (2) 3
- [(CuI)$_2$(CDP(Py)$_2$)] (3) 4
- [(CuPPh$_3$)$_2$(CDP(Py)$_2$)](PF$_6$)$_2$ (4) 4
- [(Cu(PC$_6$H$_4$OMe)$_3$)$_2$(CDP(Py)$_2$)](PF$_6$)$_2$ (5) 5
- [Cu$_2$(DPEPhos)(CDP(Py)$_2$)](PF$_6$)$_2$ (6) 5
- [Cu$_2$(XantPhos)(CDP(Py)$_2$)](PF$_6$)$_2$ (7) 6
- [Cu$_2$(dppf)(CDP(Py)$_2$)](PF$_6$)$_2$ (8) 6
- [(CuCarb)$_2$(CDP(Py)$_2$)] (11) 7
- [(CuS(C$_6$F$_5$))$_2$(CDP(Py)$_2$)] (9) 7
- (CDP(CH$_2$PPh$_2$)$_2$ (13) 8
- [(CuCl)$_2$(CDP(CH$_2$PPh$_2$)$_2$] (14) 8
- [(CuI)$_2$(CDP(CH$_2$PPh$_2$)$_2$] (15) 9
- [(CuSPh)$_2$(CDP(CH$_2$PPh$_2$)$_2$] (16) 9
- [CuCl(CDPH(CH$_2$PPh$_2$)$_2$]PF$_6$ (17) 9
- [CuPPh$_3$(CH(PPh$_2$CHPPh$_2$)$_2$] (18) 10

NMR spectroscopy 10

- [(CuCl)$_2$(CDP(Py)$_2$)] (2) 10
- [(CuI)$_2$(CDP(Py)$_2$)] (3) 12
- [(CuPPh$_3$)$_2$(CDP(Py)$_2$)](PF$_6$)$_2$ (4) 14
- [(Cu(PPhOMe)$_3$)$_2$(CDP(Py)$_2$)](PF$_6$)$_2$ (5) 15
- [Cu$_2$(DPEPhos)(CDP(Py)$_2$)](PF$_6$)$_2$ (6) 17
- [Cu$_2$(XantPhos)(CDP(Py)$_2$)](PF$_6$)$_2$ (7) 18
- [Cu$_2$(dppf)(CDP(Py)$_2$)](PF$_6$)$_2$ (8) 19

[$(CuCarb)_2(CDP(Py)_2)$] (11) 21
[$(CuS(C_6F_5))_2(CDP(Py)_2)$] (9) 23
($CDP(CH_2PPh_2)_2$ (13) 24
[$(CuCl)_2(CDP(CH_2PPh_2)_2$] (14) 25
[$(CuI)_2(CDP(CH_2PPh_2)_2$] (15) 26
[$(CuSPh)_2(CDP(CH_2PPh_2)_2$] (16) 28
[$CuCl(CDPH(CH_2PPh_2)_2$]PF_6 (17) 29
[$CuPPh_3(CH(PPh_2CHPPh_2)_2$] (18) 30

IR Spectroscopy 31

Crystallographic Data 32
[$(CuCl)_2(CDP(Py)_2)$] (2) 32
[$(CuCl)_2(CDP(Py)_2)$] (3) 33
[$(CuPPh_3)_2(CDP(Py)_2)$]$(PF_6)_2$ (4) 34
[$(Cu(PPhOMe)_3)_2(CDP(Py)_2)$]$(PF_6)_2$ (5) 35
[Cu_2(DPEPhos)($CDP(Py)_2$)]$(PF_6)_2$ (6) 36
[Cu_2(XantPhos)($CDP(Py)_2$)]$(PF_6)_2$ (7) 37
[Cu_2(dppf)($CDP(Py)_2$)]$(PF_6)_2$ (8) 38
[$(CuCarb)_2(CDP(Py)_2)$] (11) 39
[$(CuS(C_6F_5))_2(CDP(Py)_2)$] (9) 40
[$(CuCl)_2(CDP(CH_2PPh_2)_2$] (14) 41
[$(CuI)_2(CDP(CH_2PPh_2)_2$] (15) 42
[$(CuSPh)_2(CDP(CH_2PPh_2)_2$] (16) 43
[$CuCl(CDPH(CH_2PPh_2)_2$]PF_6 (17) 44
[$CuPPh_3(CH(PPh_2CHPPh_2)_2$] (18) 45

Chemical calculations 46
Deprotonation of 19 46
Kohn-Sham orbitals 52
Atomic coordinates 53

Literature 53

Cartesian coordinates of calculated structures (XYZ)……… …………………………55ff

Experimental Procedures

All reactions were carried out under inert atmosphere using standard Schlenk techniques. Air or moisture sensitive substances were stored in a nitrogen-flushed glovebox. Solvents were purified according to common literature procedures[1] and stored under an inert atmosphere over molecular sieve (3 Å or 4 Å). CDP(Py)$_2$[2] and (CH$_2$)(dppm)$_2$[3,4] were synthesized according to the literature.

^{1}H, ^{13}C and ^{31}P NMR spectra were recorded on a Bruker Avance III HD 250, Avance II 300, Avance III HD 300 or Avance III HD 500 spectrometer at room temperature if not stated differed. Chemical shift δ is denoted relatively to SiMe$_4$ (^{1}H, ^{13}C) or 85% H$_3$PO$_4$ (^{31}P). ^{1}H and ^{13}C NMR spectra were referenced to the solvent signals.[5] Multiplicity is abbreviated as follows: s (singlet), d (doublet), t (triplet), q (quartet), m (multiplet), br (broad). High-resolution mass spectrometry was performed on a Thermo Fisher Scientific LTQ-FT Ultra or a Jeol AccuTOF GCv., elemental analysis on an Elementar Vario Micro Cube. IR spectra and UV/Vis spectroscopy measurements were recorded in a glovebox on a Bruker Alpha ATR-FT-IR and with an AvaLight-DHclight source and an AvaSpec-2048 detector using quartz cuvettes and dichloromethane as solvent. UV/Vis spectra were taken at different concentrations to calculate the molar extinction coefficient by using the Beer–Lambert law. Cyclic voltammetry measurements were carried out with an Ivium Technologies Iviumstat or a Metrohm AutolabPGSTAT204 using a RHD Instruments microcell HC under nitrogen at 25 °C. A platinum working electrode (diameterd=0.25 mm) and a platinum counter electrode was used, as well as a silver/silver sulfide reference electrode. Ferrocene was used as internal standard. Dried[6] DMSO from Sigma–Aldrich was used as solvent and tetrabutylammonium hexafluorophosphate (TBAPF$_6$; ≥99.0 %, from Fluka) was used as electrolyte (50 mmol·L^{-1}) for electrochemical analysis.

Synthesis

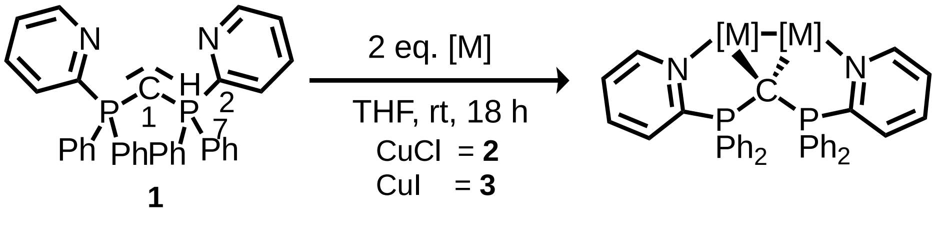

[(CuCl)$_2$(CDP(Py)$_2$)] (2)

A 446 mg portion (0.83 mmol, 1 eq.) of **1** and 164 mg (1.66 mmol, 2 eq.) of cupper(I) iodide were suspended in 15.0 ml THF at room temperature and stirred for 18 h. The solvent was then evaporated and the precipitate was washed with 5 · 15 ml of diethyl ether and 5 · 15 ml of pentane. The highly orange residue was dried in vacuo and collected in an 86% yield (523 mg, 0.71 mmol). ^{31}P{^{1}H} NMR (CCl$_2$D$_2$, 101 MHz): δ 21.4 (s) ppm. ^{1}H NMR (CCl$_2$D$_2$, 300 MHz): δ 8.84-8.83 (d, $J_{H,H}$ = 4.5 Hz, 2H, H6), 7.77-7.72 (m, 2H, H4), 7.70-7.63 (m, 8H, H8), 7.54-7.50 (m, 2H, H5), 7.44 (t, $J_{H,H}$ = 7.6 Hz, 4H, H10), 7.37-7.34 (m, 2H, H3), 7.25 (t, $J_{H,H}$ = 7.55 Hz, 8H, H9) ppm. ^{13}C{^{1}H} NMR (CCl$_2$D$_2$, 75 MHz): δ

155.0-153.8 (m, *C*2), 149.9 (t, $^{3}J_{C,P}$ = 8.12 Hz, *C*6), 137.9 (t, ^{3}JC,P = 5.03 Hz, *C*4), 133.4 (t, $^{2}J_{C,P}$ = 4.97 Hz, *C*8), 132.6 (s, *C*10), 129.1 (t, $^{3}J_{C,P}$ = 5.82 Hz, *C*9), 128.8-127.8 (m, *C*7), 128.0 (m, *C*3), 126.8 (s, *C*5), -3.9 (m, *C*1) ppm. APCI-MS: *m/z* 735.1 $[M+H]^{+}$. Elem. anal. (%)[1] found C, 56.49; H, 3.92; N, 4.05; $C_{35}H_{28}N_2P_2Cu_2Cl_2$ required C, 57.07; H, 3.83; N, 3.80.

[(CuI)$_2$(CDP(Py)$_2$)] (3)

A 100 mg portion (0.19 mmol, 1 eq.) of **1** and 71 mg (0.37 mmol, 2 eq.) of cupper(I) iodide were suspended in 5.0 ml THF at room temperature and stirred for 18 h. The solvent was then evaporated and the precipitate was washed with 5 · 5 ml of diethyl ether and 5 · 5 ml of pentane. The highly orange residue was dried in vacuo and collected in a 63% yield (107 mg, 0.12 mmol). $^{31}P\{^{1}H\}$ NMR (CCl_2D_2, 101 MHz): δ 21.5 (s) ppm. ^{1}H NMR (CCl_2D_2, 300 MHz): δ 8.98-8.97 (d, $J_{H,H}$ = 4.9 Hz, 2H, *H*6), 7.79-7.75 (m, 2H, *H*4), 7.65-7.59 (m, 10H, *H*8, *H*5), 7.44 (tt, $J_{H,H}$ =0.8, 7.5 Hz, 4H, *H*10), 7.39-7.36 (m, 2H, *H*3), 7.26-7.3 (m, 8H, *H*9) ppm. $^{13}C\{^{1}H\}$ NMR (CCl_2D_2, 75 MHz): δ 150.9 (m, *C*6), 137.9 (t, ^{3}JC,P = 5.5 Hz, *C*4), 133.6 (t, $^{2}J_{C,P}$ = 4.9 Hz, *C*8), 132.7 (s, *C*10), 129.3 (t, $^{3}J_{C,P}$ = 5.7 Hz, *C*9), 128.7 (m, *C*7), 128.3 (s, *C*3), 127.2 (s, *C*5) ppm. Elem. anal. (%) found C, 45.52; H, 3.32; N, 3.25; $C_{35}H_{28}N_2P_2Cu_2Cl_2$ required C, 45.72; H, 3.07; N, 3.05.

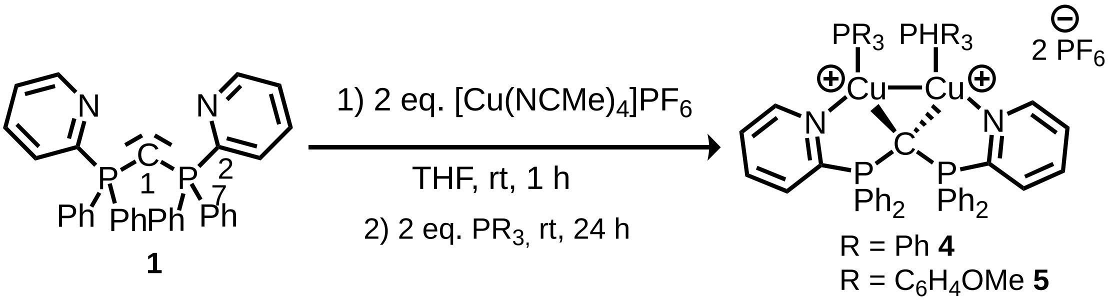

[(CuPPh$_3$)$_2$(CDP(Py)$_2$)](PF$_6$)$_2$ (4)

A 50.0 mg portion (0.09 mmol, 1 eq.) of **1** and 69.2 mg (0.18 mmol, 2 eq.) of tetrakis(acetonitrile)copper(I) hexafluorophosphate were treated with 5.0 ml THF at room temperature and stirred for 1 h. A change in colour was observed (red→dark red→brown→orange→clear yellow). A 48.7 mg portion (0.18 mmol, 2 eq.) of triphenylphosphine was added and the mixture stirred for additional 24 h. The solvent was then removed and the precipitate formed was washed with 2 · 5 ml of diethyl ether and 2 · 5 ml of pentane. The light yellow residue was dried in vacuo and collected in a 72%

[1] As a matter of fact, the combustion analysis of air sensitive metal containing compounds is sometimes tricky in our departmental analytic lab: We learnt from experimental deviations observed in the range 0.5 up to 1,5% from one and the same phase pure, single crystalline metal organic sample, that such deviations - rather untypical for purely organic non-air sensitive reference compounds - have to be expected in some cases. Deviations are dependent on the time between weighting the 4 mg sample into the vial (under a blanket of argon), putting the vial into the vial holder of the sample carousel and the delay time of its combustion. Finally, they are dependent on the type of metal, the combustion temperature, additives for better combustion and for less formation of metal carbides and nitrides. As we have isolated nearly all compounds in single crystalline phase and have characterized even more than those reported here via XRD analysis, we are absolutely confident, that all compounds described herein were isolated in analytically phase pure form – sometimes with additional solvent molecules in the lattice, difficult to eliminate under vacuum and higher temperatures.

yield (100 mg, 0.07 mmol). $^{31}P\{^1H\}$ NMR (CCl_2D_2, 101 MHz): δ 32.9 (s), 3.5 (s), 145.1 (sep.) ppm. ^1H NMR (CCl_2D_2, 300 MHz): δ 8.13 (t, $J_{H,H}$ = 7.5 Hz, 2H, CH_{arom}), 7.96 (d, $J_{H,H}$ =4.8 Hz, 2H, CH_{arom}), 7.71-7.68 (m, 2H, CH_{arom}), 7.57 (t, $J_{H,H}$ = 6.0 Hz, 2H, CH_{arom}), 7.47 (q, $J_{H,H}$ = 8.4 Hz, 10H, CH_{arom}), 7.28-7.15 (m, 20H, CH_{arom}), 7.07 (t, $J_{H,H}$ =7.2 Hz, 8H, CH_{arom}), 6.93 (dd, $J_{H,H}$ =7.4, 11.3 Hz, 12H, CH_{arom}) ppm. $^{13}C\{^1H\}$ NMR (CCl_2D_2, 75 MHz): δ 151.3, 146.2, 141.0, 134.3, 133.9, 133.7, 133.2 (t, $^2J_{C,P}$ = 4.8 Hz), 132.1, 130.8, 130.3, 130.2, 130.2, 130.1, 130.0, 129.5 ppm. Elem. anal. (%) found C, 56.36; H, 4.26; N, 1.92; $C_{71}H_{58}N_2P_6Cu_2F_{12}$ required C, 57.61; H, 3.95; N, 1.89. ϕ_{PL} = 36%.

[(Cu(PC$_6$H$_4$OMe)$_3$)$_2$(CDP(Py)$_2$)](PF$_6$)$_2$ (5)

A 50.0 mg portion (0.09 mmol, 1 eq.) of **1** and 69.2 mg (0.18 mmol, 2 eq.) of tetrakis(acetonitrile)copper(I) hexafluorophosphate were treated with 5.0 ml THF at room temperature and stirred for 1 h. A change in colour was observed (red→dark red→brown→orange→clear yellow). A 66.0 mg portion (0.18 mmol, 2 eq.) of tris(*o*-methoxyphenyl)phosphine was added and the mixture stirred for additional 24 h. The solvent was then removed and the precipitate formed was washed with 2 · 5 ml of diethyl ether and 2 · 5 ml of pentane. The light yellow residue was dried in vacuo and collected in a 47% yield (72.0 mg, 0.04 mmol). $^{31}P\{^1H\}$ NMR (CCl_2D_2, 101 MHz): δ 32.8 (s), –0.3 (s), 144.5 (sep.) ppm. ^1H NMR (CCl_2D_2, 300 MHz): δ 8.12-8.07 (m, 4H, CH_{arom}), 7.72-7.69(m, 2H, CH_{arom}), 7.61 (t, $J_{H,H}$ = 5.7 Hz, 2H, CH_{arom}), 7.47 (t, $J_{H,H}$ = 6.3 Hz, 4H, CH_{arom}), 7.47 (q, $J_{H,H}$ = 8.4 Hz, 10H, CH_{arom}), 7.28-7.27 (m, 8H, CH_{arom}), 7.12-7.08 (m, 8H, CH_{arom}), 6.87-6.81 (m, 12H, CH_{arom}), 6.64 d, $J_{H,H}$ = 7.7 Hz, 2H, CH_{arom}), 3.79 (s, 18H, 6 · CH_3) ppm. $^{13}C\{^1H\}$ NMR (CCl_2D_2, 75 MHz): δ 162.6, 151.4, 140.7, 135.3 (d, $^2J_{C,P}$ = 16.1 Hz), 134.2, 133.3 (t, $^2J_{C,P}$ = 4.9 Hz), 130.1 (t, $^2J_{C,P}$ = 6.0 Hz), 129.2, 122.4, 121.8, 115.6 (d, $^2J_{C,P}$ = 11.2 Hz), 56.1 ppm. Elem. anal. (%) found C, 57.98; H, 4.29; N, 1.89; $C_{77}H_{70}N_2P_6O_6Cu_2F_{12}$ required C, 57.07; H, 3.78; N, 1.87.

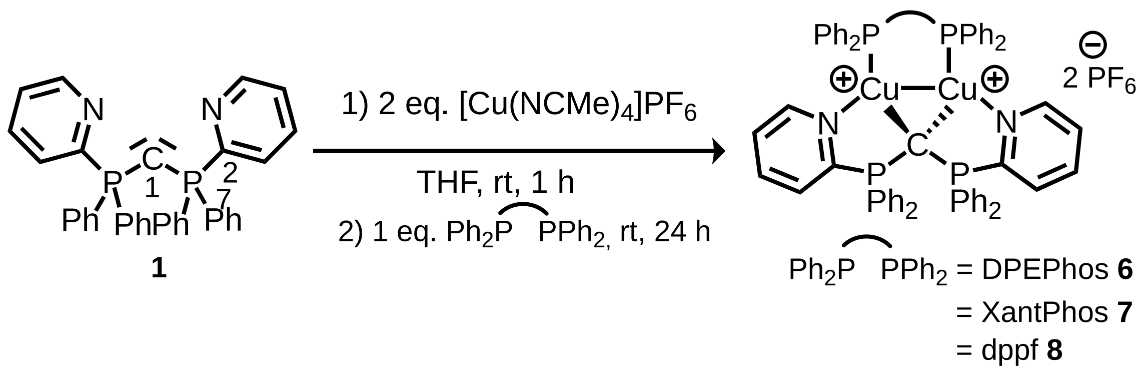

[Cu$_2$(DPEPhos)(CDP(Py)$_2$)](PF$_6$)$_2$ (6)

A 50.0 mg portion (0.09 mmol, 1 eq.) of **1** and 69.2 mg (0.18 mmol, 2 eq.) of tetrakis(acetonitrile)copper(I) hexafluorophosphate were treated with 5.0 ml THF at room temperature and stirred for 1 h. A change in colour was observed (red→dark red→brown→orange→clear yellow). A 50.0 mg portion (0.09 mmol, 1 eq.) of bis[(2-diphenylphosphino)phenyl] ether was added and the mixture stirred for additional 24 h.

The solvent was then removed and the precipitate formed was washed with 2 · 5 ml of diethyl ether and 2 · 5 ml of pentane. The light yellow residue was dried in vacuo and collected in an 86% yield (120.0 mg, 0.08 mmol). $^{31}P\{^{1}H\}$ NMR (CCl_2D_2, 101 MHz): δ 29.7 (s), –8.7 (s), 144.5 (sep.) ppm. ^{1}H NMR (CCl_2D_2, 300 MHz): δ 8.05-7.99 (m, 2H, CH_{arom}), 7.83-7.79(m, 2H, CH_{arom}), 7.71-7.64 (m, 4H, CH_{arom}), 7.55-7.51 (m, 2H, CH_{arom}), 7.46-7.34 (m, 12H, CH_{arom}), 7.30-7.26 (m, 4H, CH_{arom}), 7.21-7.06 (m, 18H, CH_{arom}), 6.91-6.75 (m, 10H, CH_{arom}), 6.70-6.65 (m, 2H, CH_{arom}). Elem. anal. (%) found C, 56.42; H, 4.06; N, 1.51; $C_{71}H_{56}N_2P_6Cu_2F_{12}O$ required C, 56.13; H, 4.53; N, 1.66.

$[Cu_2(XantPhos)(CDP(Py)_2)](PF_6)_2$ (7)

A 50.0 mg portion (0.09 mmol, 1 eq.) of **1** and 69.9 mg (0.18 mmol, 2 eq.) of tetrakis(acetonitrile)copper(I) hexafluorophosphate were treated with 5.0 ml THF at room temperature and stirred for 1 h. A change in colour was observed (red→dark red→brown→orange→clear yellow). A 53.7 mg portion (0.09 mmol, 1 eq.) of 4,5-bis(diphenylphosphino)-9,9-dimethylxanthene was added and the mixture stirred for additional 24 h. The solvent was then removed and the precipitate formed was washed with 2 · 5 ml of diethyl ether and 2 · 5 ml of pentane. The light yellow residue was dried in vacuo and collected in an 81% yield (93.0 mg, 0.08 mmol). $^{31}P\{^{1}H\}$ NMR (CCl_2D_2, 101 MHz): δ 34.9 (s), –6.1 (s), 143.8 (sep.) ppm. ^{1}H NMR (CCl_2D_2, 300 MHz): δ 8.13 (d, $J_{H,H}$ = 4.6 Hz, 2H, CH_{arom}), 7.98 (t, $J_{H,H}$ = 7.0 Hz, 2H, CH_{arom}), 7.64 (d, $J_{H,H}$ = 6.8 Hz, 2H, CH_{arom}), 7.54-7.43 (m, 10H, CH_{arom}), 7.43 (br.s, 2H, CH_{arom}), 7.19-7.07 (m, 22H, CH_{arom}), 6.78 (br.s, 10H, CH_{arom}), 6.40 (t, $J_{H,H}$ = 8.3 Hz, 2H, CH_{arom}), 1.65 (s, 6H, 2 · CH_3). $^{13}C\{^{1}H\}$ NMR (CCl_2D, 75 MHz): δ 156.2, 155.5, 151.6 (d, $^{2}J_{C,P}$ = 8.8 Hz), 151.3(t, $^{2}J_{C,P}$ = 6.7 Hz), 140.3 (t, $^{2}J_{C,P}$ = 4.7 Hz), 134.2, 133.9 (t, $^{2}J_{C,P}$ = 7.5 Hz), 133.7, 133.4, 133.1, 132.3, 131.0, 130.4, 130.3, 130.1 (d, $^{2}J_{C,P}$ = 10.1 Hz), 129.8, 129.4, 129.0, 125.1 (d, $^{2}J_{C,P}$ = 6.1 Hz), 118.0, 117.7, 34.8, 33.4 ppm. Elem. anal. (%) found C, 57.01; H, 4.16; N, 2.09; $C_{74}H_{60}N_2P_6Cu_2F_{12}O$ required C, 57.93; H, 3.94; N, 1.83.

$[Cu_2(dppf)(CDP(Py)_2)](PF_6)_2$ (8)

A 50.0 mg portion (0.09 mmol, 1 eq.) of **1** and 69.9 mg (0.18 mmol, 2 eq.) of tetrakis(acetonitrile)copper(I) hexafluorophosphate were treated with 5.0 ml THF at room temperature and stirred for 1 h. A change in colour was observed (red→dark red→brown→orange→clear yellow). A 51.5 mg portion (0.09 mmol, 1 eq.) of 1,1'-bis(diphenylphosphino)ferrocene was added and the mixture stirred for additional 24 h. The solvent was then removed and the precipitate formed was washed with 2 · 5 ml of diethyl ether and 2 · 5 ml of pentane. The light yellow residue was dried in vacuo and collected in a 90% yield (102.0 mg, 0.08 mmol). $^{31}P\{^{1}H\}$ NMR (CCl_2D_2, 101 MHz): δ 36.5 (s), –3.1 (s), 143.9 (sep.) ppm. ^{1}H NMR (CCl_2D_2, 300 MHz): δ 8.33 (d, $J_{H,H}$ = 4.8 Hz, 2H, CH_{arom}), 8.27 (t, $J_{H,H}$ = 6.9 Hz, 2H, CH_{arom}), 7.83-7.79 (m, 4H, CH_{arom}), 7.53-717 (m, 30H, CH_{arom}), 7.00 (br.s, 8H, CH_{arom}), 4.45-4.21 (m, 10H, CpH) ppm. $^{13}C\{^{1}H\}$ NMR (CCl_2D_2, 75 MHz): δ 155.9, 151.9, 140.9, 134.2, 133.1, 131.7, 130.3 (t, $^{2}J_{C,P}$ = 5.9 Hz), 129.6, 73.8,

73.1, 72.2, 71.8 ppm. Elem. anal. (%) found C, 53.26; H, 3.83; N, 1.98; $C_{69}H_{56}N_2P_6Cu_2F_{12}Fe$ required C, 53.78; H, 3.76; N, 1.98.

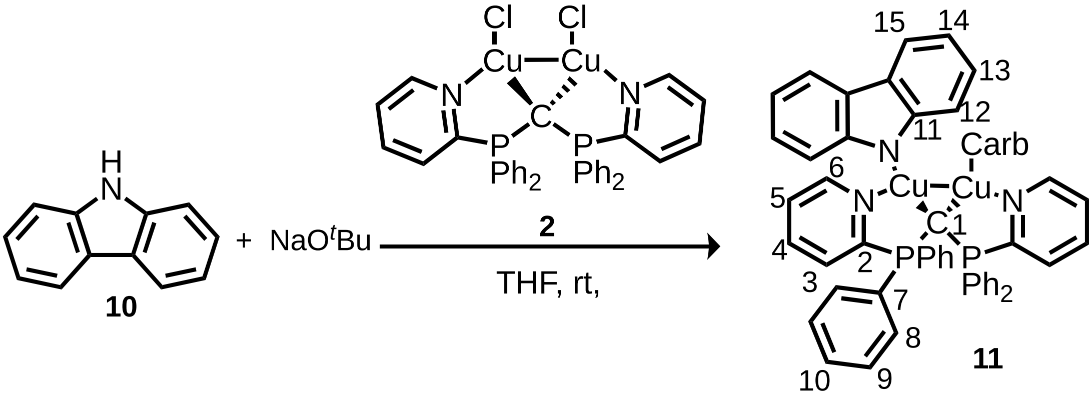

[(CuCarb)$_2$(CDP(Py)$_2$)] (11)

A 23.0 mg portion (0.14 mmol, 2 eq.) of carbazole (**10**) and 13.0 mg (0.14 mmol, 2 eq.) of sodium *tert*-butoxide were treated with 2.0 ml THF at room temperature and stirred for 3 h. A suspension of 50.0 mg of [(CuCl)$_2$(CDP(Py)$_2$] (**2**, 0.07 mmol, 1 eq.) in 2.0 ml THF was added and the reaction mixture stirred for additional 70 h at room temperature. The solvent was then removed and the precipitate formed was washed with 3 · 5 ml of diethyl ether and 3 · 5 ml of pentane. The light orange residue was dried in vacuo and collected in a 56% yield (38.0 mg, 0.04 mmol). $^{31}P\{^1H\}$ NMR (CCl_2D_2, 101 MHz): δ 22.8 ppm. ^1H NMR (CCl_2D_2, 300 MHz): δ 8.50 (d, $J_{H,H}$ = 4.7 Hz, 2H, *H*6), 7.97 (dd, $J_{H,H}$ = 0.4, 7.5 Hz, 4H, *H*11), 7.94 (ddd, $J_{H,H}$ = 1.1, 8.3, 12.3 Hz, 8H, *H*8), 7.60 (ddt, $J_{H,H}$ = 1.8, 4.9, 7.7 Hz, 2H, *H*4), 7.42 (t, $J_{H,H}$ = 7.4 Hz, 4H, *H*10), 7.34-7.30 (m, 4H, *H*5,*H*3), 7.22-7.18 (m, 12H, *H*9,*H*12), 6.95 (dt, $J_{H,H}$ = 1.3, 7.0 Hz, 4H, *H*13), 6.95 (dt, $J_{H,H}$ = 0.9, 7.3 Hz, 4H, *H*14) ppm. $^{13}C\{^1H\}$ NMR (CCl_2D_2, 75 MHz): δ 155.4-154.2(m, *C*2), 151.2 (s, *C*16), 150.6 (t, $^2J_{C,P}$ = 8.3 Hz, *C*6), 137.9 (t, $^2J_{C,P}$ = 5.1 Hz, *C*4), 133.8 (t, $^2J_{C,P}$ = 4.8 Hz, *C*8), 132.8 (s, *C*10), 129.3 (t, $^2J_{C,P}$ = 5.9 Hz, *C*9), 128.6-127.7 (m, *C*3,*C*7), 126.7 (s, *C*5), 124.5 (s, *C*15), 123.5 (s, *C*13), 119.6 (s, *C*11), 115.3 (s, *C*12), 114.9 (s, *C*13), –0.9 (t, $^2J_{C,P}$ = 54.0 Hz, *C*1) ppm. LIFDI-HRMS: *m/z* 539.18016. $[M-H]^+$. Elem. anal. (%) found C, 70.68; H, 4.44; N, 5.61; $C_{59}H_{44}N_4P_2Cu_2$ required C, 71.00; H, 4.66; N, 5.01.

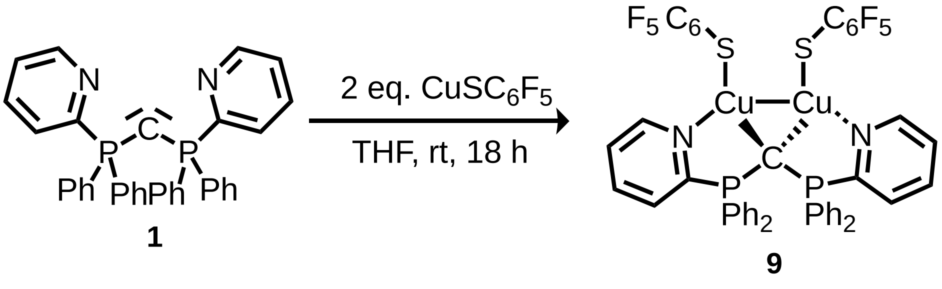

[(CuS(C$_6$F$_5$))$_2$(CDP(Py)$_2$)] (9)

A 76.0 mg portion (0.14 mmol, 1 eq.) of **1** and 74.0 mg (0.28 mmol, 2 eq.) of cupper(I) pentafluorothiophenolate were suspended in 5.0 ml THF at room temperature and stirred for 18 h. The solvent was then evaporated and the precipitate was washed with 5 · 15 ml of diethyl ether and 5 · 15 ml of pentane. The light orange residue was dried

in vacuo and collected in a 27% yield (40.0 mg, 0.04 mmol). $^{31}P\{^{1}H\}$ NMR (CCl_2D_2, 101 MHz): δ 23.1 ppm. ^{1}H NMR (CCl_2D_2, 300 MHz): δ 8.81-8.66 (m, 2H, CH_{arom}), 7.94-7.87 (m, 5H, CH_{arom}), 7.72-7.66 (m, 2H, CH_{arom}), 7.60-7.47 (m, 10H, CH_{arom}), 7.46-7.40 (m, 4H, CH_{arom}), 7.27-7.12 (m, 5H, CH_{arom}) ppm. Elem. anal. (%) found C, 42.47; H, 2.24; N, 2.28; $C_{47}H_{28}P_4F_{22}N_2S_2$ required C, 41.70; H, 2.08; N, 2.07.

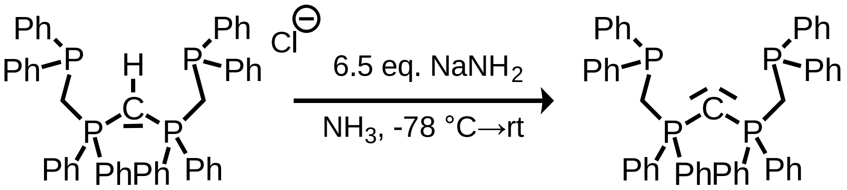

12 **13**

(CDP(CH₂PPh₂)₂ (13)

A 250.0 mg portion (0.30 mmol, 1.0 eq.) of [CH(dppm)$_2$]Cl[3,7] (**12**) and 80.4 mg $NaNH_2$ (2.1 mmol, 6.5 eq.) were suspended in NH_3 (l.) at –78 °C and stirred till the temperature reached room temperature, causing the NH_3 to vaporize. The light yellow residue was disolved in toluene and the feed was removed via filtration. The solvent was then evaporated and the precipitate was washed with 2 · 15 ml of pentane. The yellow residue was dried in vacuo and collected in a 98% yield (233.1 mg, 0.30 mmol). $^{31}P\{^{1}H\}$ NMR (C_6D_6, 101 MHz): δ 23.2 (dt, J = 8.0, 14.7 Hz), –15.8 (d, J = 118.5 Hz) ppm. ^{1}H NMR (CCl_2D_2, 300 MHz): δ 7.09-7.71 (m, 8H, CH_{arom}), 7.51-7.31 (m, 8H, CH_{arom}), 7.08-6.88 (m, 24H, CH_{arom}), 3.44 (br.s, 3H, CH/CH_2), 1.44 (s, 1H, CH) ppm. APCI-HRMS: m/z 781.24799 $[M+H]^+$. Elem. anal. (%) found C, 77.99; H, 5.74; $C_{51}H_{44}P_4$ required C, 78.45; H, 5.68.

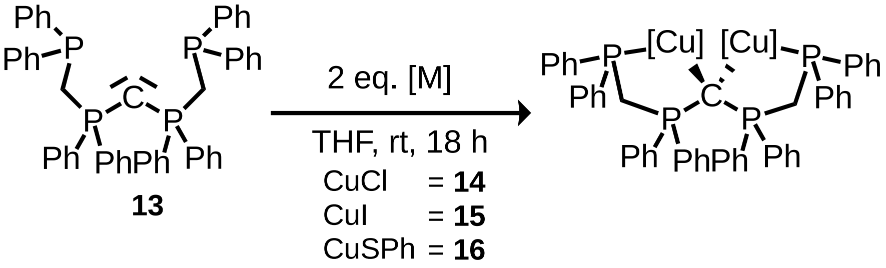

[(CuCl)₂(CDP(CH₂PPh₂)₂] (14)

A 50.0 mg portion (0.06 mmol, 1 eq.) of **13** and 12.9 mg (0.12 mmol, 2 eq.) of cupper(I) chloride were suspended in 5.0 ml THF at room temperature and stirred for 1 h. The solvent was then evaporated and the precipitate was washed with 3 · 15 ml of diethyl ether and 2 · 15 ml of pentane. The light orange residue was dried in vacuo and collected in a 77% yield (48.1 mg, 0.05 mmol). $^{31}P\{^{1}H\}$ NMR (CCl_2D_2, 101 MHz): δ 20.4 (dd, J = 34.6, 49.9 Hz), –32.2-–32.9 (m) ppm. ^{31}P-^{1}H NMR (CCl_2D_2, 101 MHz): δ 20.1 (m), –32.5 (m) ppm. ^{1}H NMR (CCl_2D_2, 300 MHz): δ 7.45 (m, 18H, CH_{arom}), 7.18 (m, 16H, CH_{arom}), 6.95 (m, 6H, CH_{arom}), 6.79 (br.s, 6H, CH_{arom}), 4.06 (br.s, 4H, 2 · CH_2) ppm. APCI-HRMS: m/z 941.07087 $[M-Cl]^+$.

[(CuI)$_2$(CDP(CH$_2$PPh$_2$)$_2$] (15)

A 50.0 mg portion (0.06 mmol, 1 eq.) of **13** and 24.5 mg (0.12 mmol, 2 eq.) of cupper(I) iodide were suspended in 5.0 ml THF at room temperature and stirred for 2 h. The solvent was then evaporated and the precipitate was washed with 3 · 15 ml of diethyl ether and 2 · 15 ml of pentane. The light orange residue was dried in vacuo and collected in a 83% yield (62.0 mg, 0.05 mmol). $^{31}P\{^1H\}$ NMR (CCl_2D_2, 101 MHz): δ 22.2 (dd, J = 36.9, 55.4 Hz), –37.6-–38.2 (m) ppm. ^{31}P-^1H NMR (CCl_2D_2, 101 MHz): δ 22.5 (m), –38.0 (m) ppm. ^1H NMR (CCl_2D_2, 300 MHz): δ 7.72-7.31 (m, 16H, CH_{arom}), 7.17-6.70 (m, 24H, CH_{arom}), 6.79 (br.s, 6H, CH_{arom}), 4.19 (br.s, 4H, 2 · CH_2) ppm. APCI-HRMS: m/z 1160.9147 $[M+H]^+$, 971.0806 $[M-CuI+H]^+$, 781.2461 $[M-(CuI)_2+H]^+$.

[(CuSPh)$_2$(CDP(CH$_2$PPh$_2$)$_2$] (16)

A 58.0 mg portion (0.07 mmol, 1 eq.) of **13** and 26.0 mg (0.15 mmol, 2 eq.) of cupper(I) thiophenolate were suspended in 5.0 ml THF at room temperature and stirred for 42 h. The solvent was then evaporated and the precipitate was washed with 3 · 15 ml of diethyl ether and 2 · 15 ml of pentane. The light orange residue was dried in vacuo and collected in a 52% yield (58.0 mg, 0.04 mmol). $^{31}P\{^1H\}$ NMR (CCl_2D_2, 101 MHz): δ 19.8-19.0 (m), –33.1-–33.9 ppm. ^1H NMR (CCl_2D_2, 300 MHz): δ 7.45-7.30 (m, 22H, CH_{arom}), 7.16-7.05 (m, 10H, CH_{arom}), 6.97-6.92 (m, 12H, CH_{arom}), 6.79 (br.s, 6H, CH_{arom}), 4.11 (br.s, 4H, 2 · CH_2) ppm.

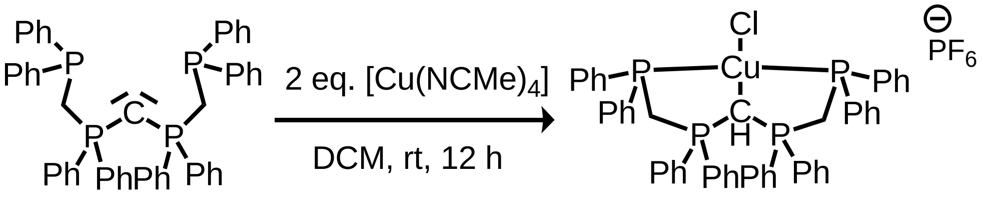

13 **17**

[CuCl(CDPH(CH$_2$PPh$_2$)$_2$]PF$_6$ (17)

A 57.2 mg portion (0.07 mmol, 1 eq.) of **13** and 27.2 mg (0.07 mmol, 1 eq.) of tetrakis(acetonitrile)copper(I) hexafluorophosphate were suspended in 5.0 ml DCM at room temperature and stirred for 12 h. The solvent was then evaporated and the precipitate was washed with 3 · 15 ml of diethyl ether and 2 · 15 ml of pentane. The light orange residue was dried in vacuo and collected in a 74% yield (55.2 mg, 0.05 mmol). $^{31}P\{^1H\}$ NMR (CCl_2D_2, 101 MHz): δ 20.8-19.2 (m), –29.6-–30.4 (m), –32.0-–33.0 (m) ppm. $^{19}F\{H\}$ NMR (CCl_2D_2, 282 MHz): δ 71.7 (d, J = 711 Hz).

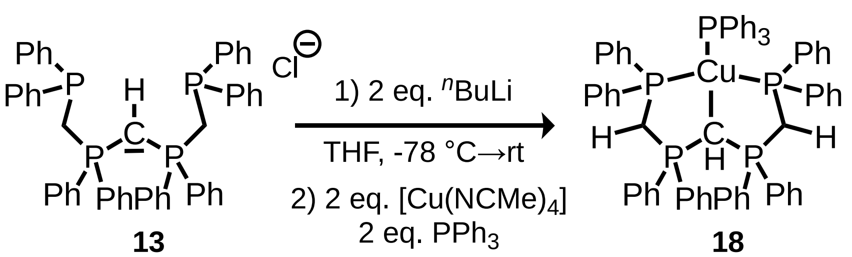

13 **18**

[CuPPh$_3$(CH(PPh$_2$CHPPh$_2$)$_2$] (18)

A solution of 100 mg of [CH(dppm)$_2$]Cl (**12**) (0.12 mmol, 1 eq.) in 10 ml of THF was treated with 1.0 ml of ^{n}BuLi in hexane (2.5 M, 0.24 mmol, 2.0 eq.) at –78 °C, while stirring. The reaction mixture was allowed to reach room temperature before a 55.7 mg portion (0.24, 2.0 eq.) of tetrakis(acetonitrile)copper(I) hexafluorophosphate was added. After stirring for an additional 1 h a 64.2 mg portion (0.24, 2.0 eq.) of triphenylphosphine was added and the mixture stirred for additional 15 h. The solvent was then removed and the precipitate formed was washed with 2 · 5 ml of diethyl ether and 2 · 5 ml of pentane. The light yellow residue was dried in vacuo and collected in a 73% yield (98.0 mg, 0.08 mmol). $^{31}P\{^1H\}$ NMR (CCl_2D_2, 101 MHz): δ 23.6 (d, $J_{P,P}$ = 142.2 Hz), 14.4 (d, $J_{P,P}$ = 118.7 Hz), –15.6 (d, $J_{P,P}$ = 142.2 Hz) ppm. ^1H NMR (CCl_2D_2, 300 MHz): δ 7.70-7.64 (m, 4H, CH_{arom}), 7.55-7.38(m, 6H, CH_{arom}), 7.34-7.12(m, 16H, CH_{arom}), 7.09-6.99 (m, 10H, CH_{arom}), 6.97-6.42 (m, 20H, CH_{arom}), 2.24 (t, $J_{P,H}$ = 8.2 Hz, 1H, CH), 1.66-1.61 (m, 2H, 2 · CH) ppm. Elem. anal. (%) found C, 74.67; H, 5.19; $C_{69}H_{59}P_5Cu$ required C, 74.89; H, 5.37.

NMR spectroscopy

[(CuCl)$_2$(CDP(Py)$_2$)] (2)

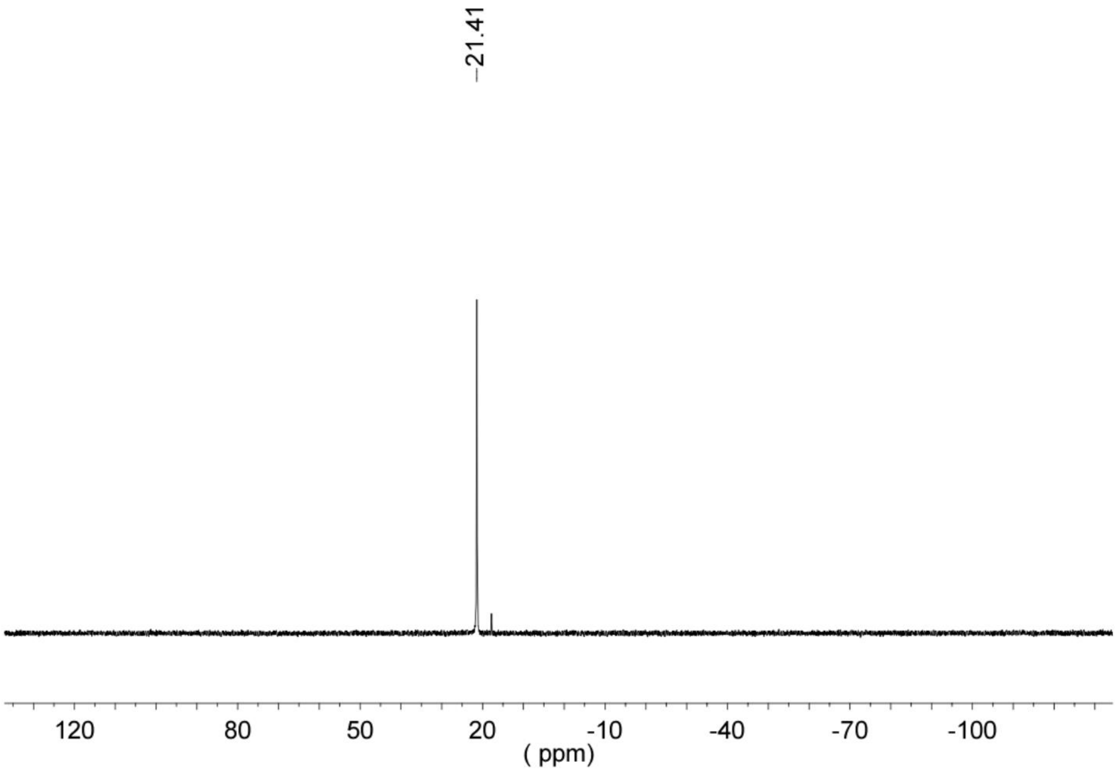

Figure S-1. $^{31}P\{^1H\}$ NMR (CCl_2D_2, 101 MHz, [ppm]) of [(CuCl)$_2$(CDP(Py)$_2$)] (**2**).

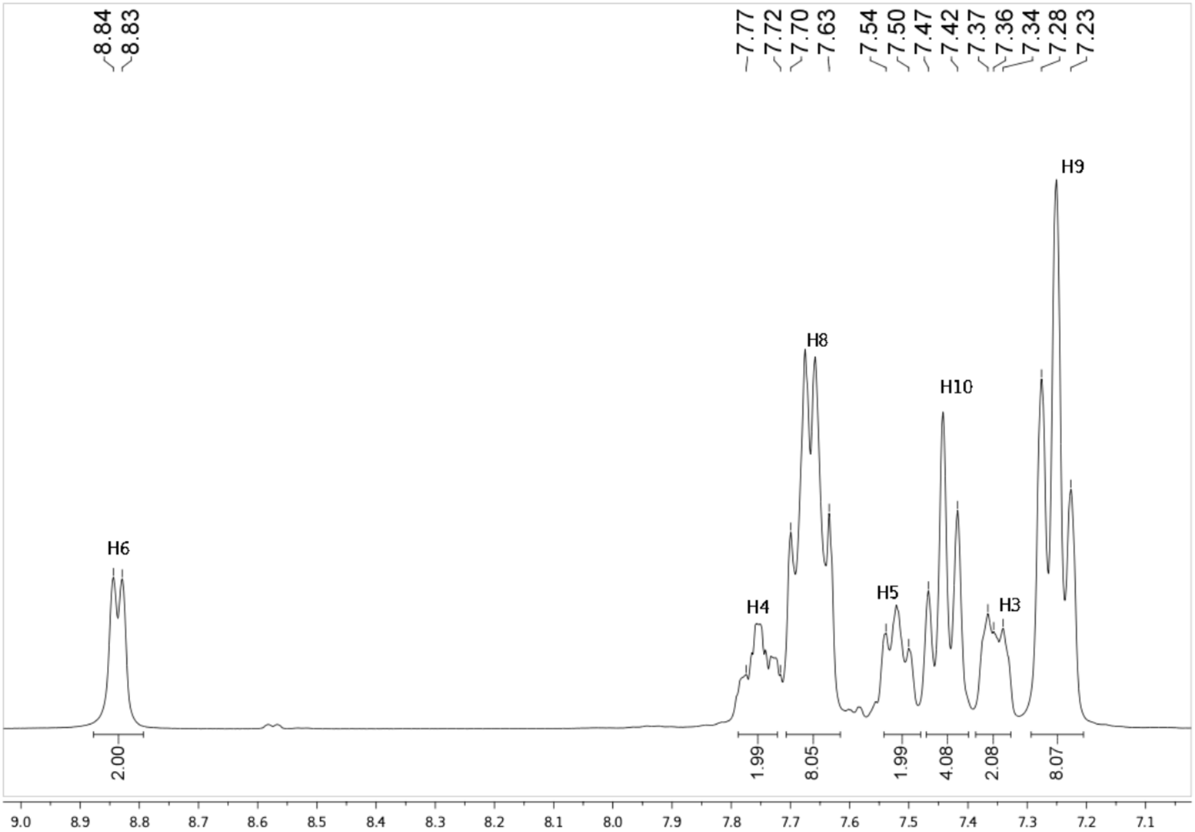

Figure S-2. ^{1}H NMR (CCl_2D_2, 300 MHz, [ppm]) of [$(CuCl)_2(CDP(Py)_2)$] (**2**).

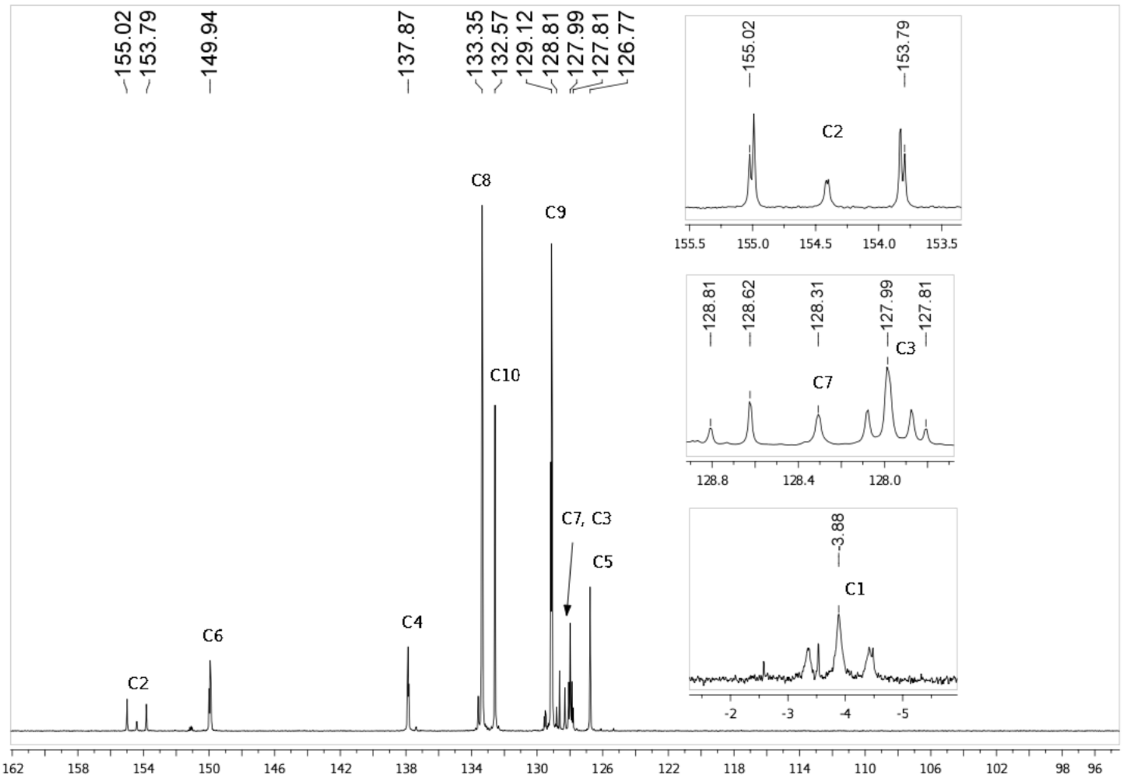

Figure S-3. $^{13}C\{^{1}H\}$ NMR (CCl_2D_2, 75 MHz, [ppm]) of [$(CuCl)_2(CDP(Py)_2)$] (**2**).

[(CuI)$_2$(CDP(Py)$_2$)] (3)

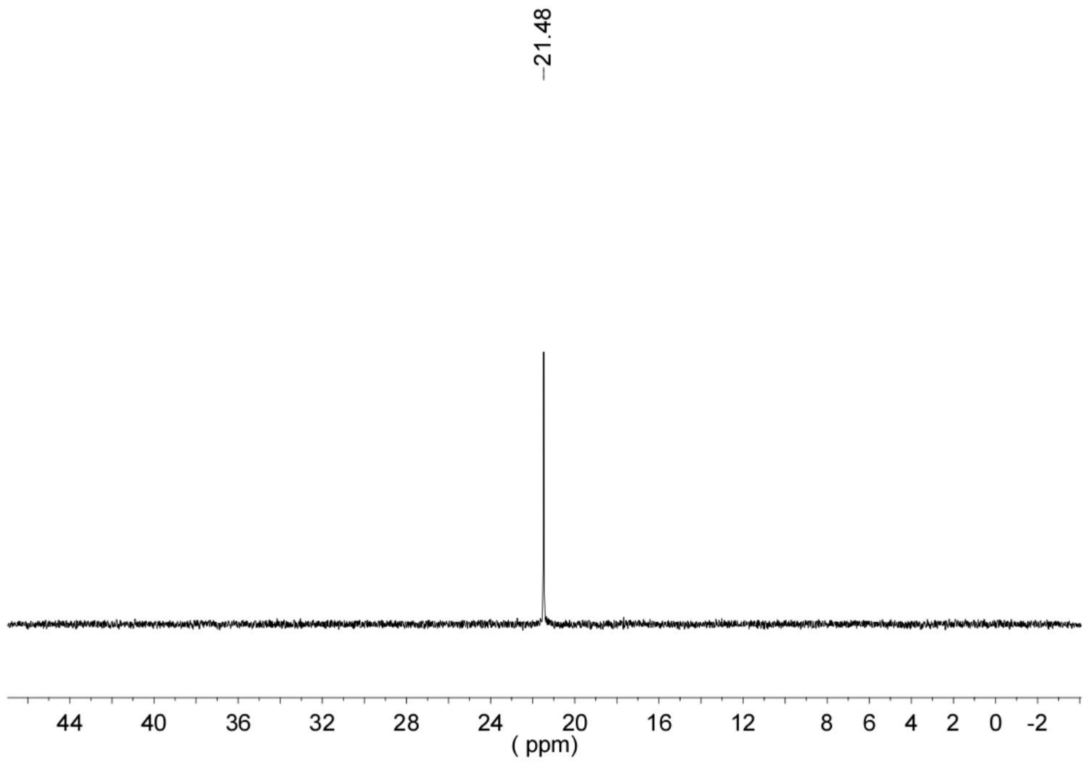

Figure S-4. $^{31}P\{^1H\}$ NMR (CCl_2D_2, 101 MHz, [ppm]) of [(CuI)$_2$(CDP(Py)$_2$)] (**3**).

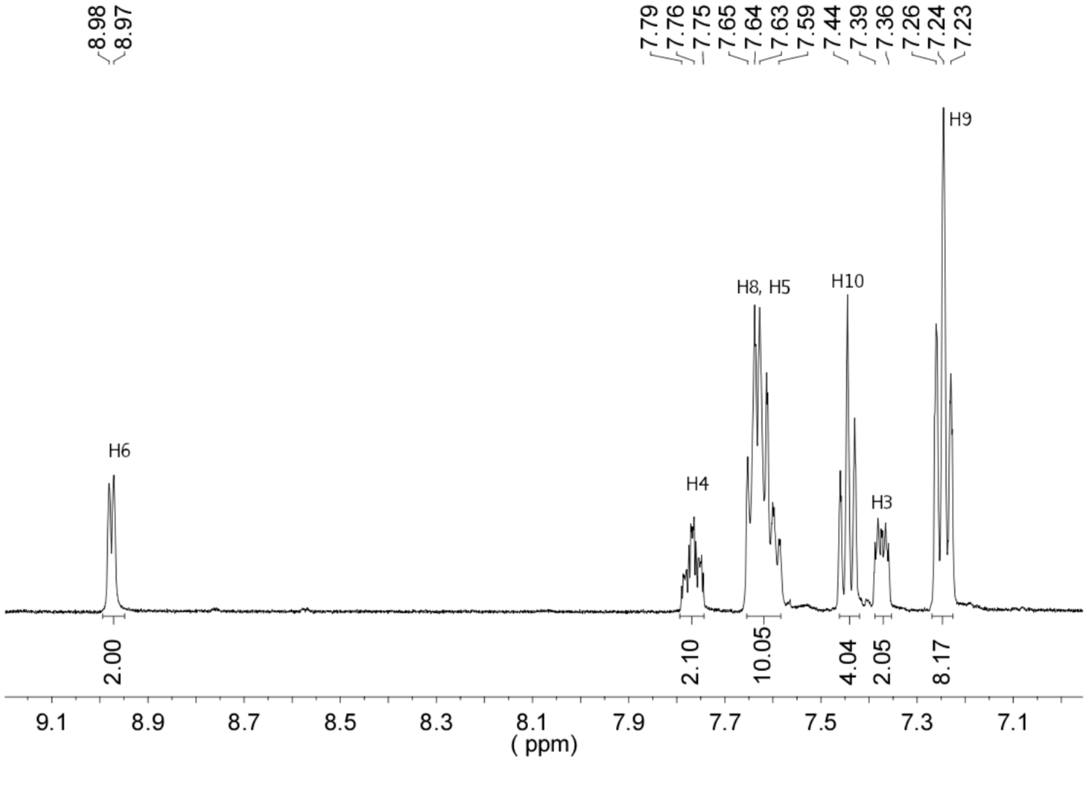

Figure S-5. ^1H NMR (CCl_2D_2, 300 MHz, [ppm]) of [(CuI)$_2$(CDP(Py)$_2$)] (**3**).

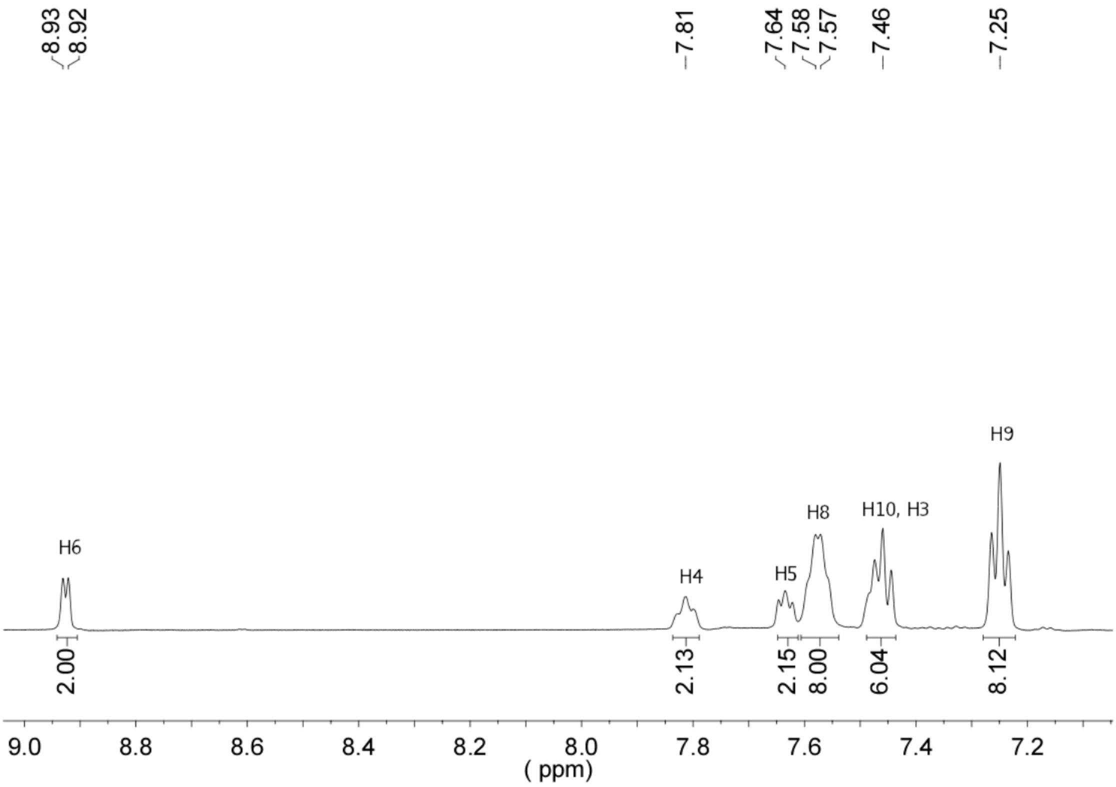

Figure S-6. ^{1}H NMR (CCl_2D_2, 200 K, 300 MHz, [ppm]) of [$(CuI)_2(CDP(Py)_2)$] (**3**).

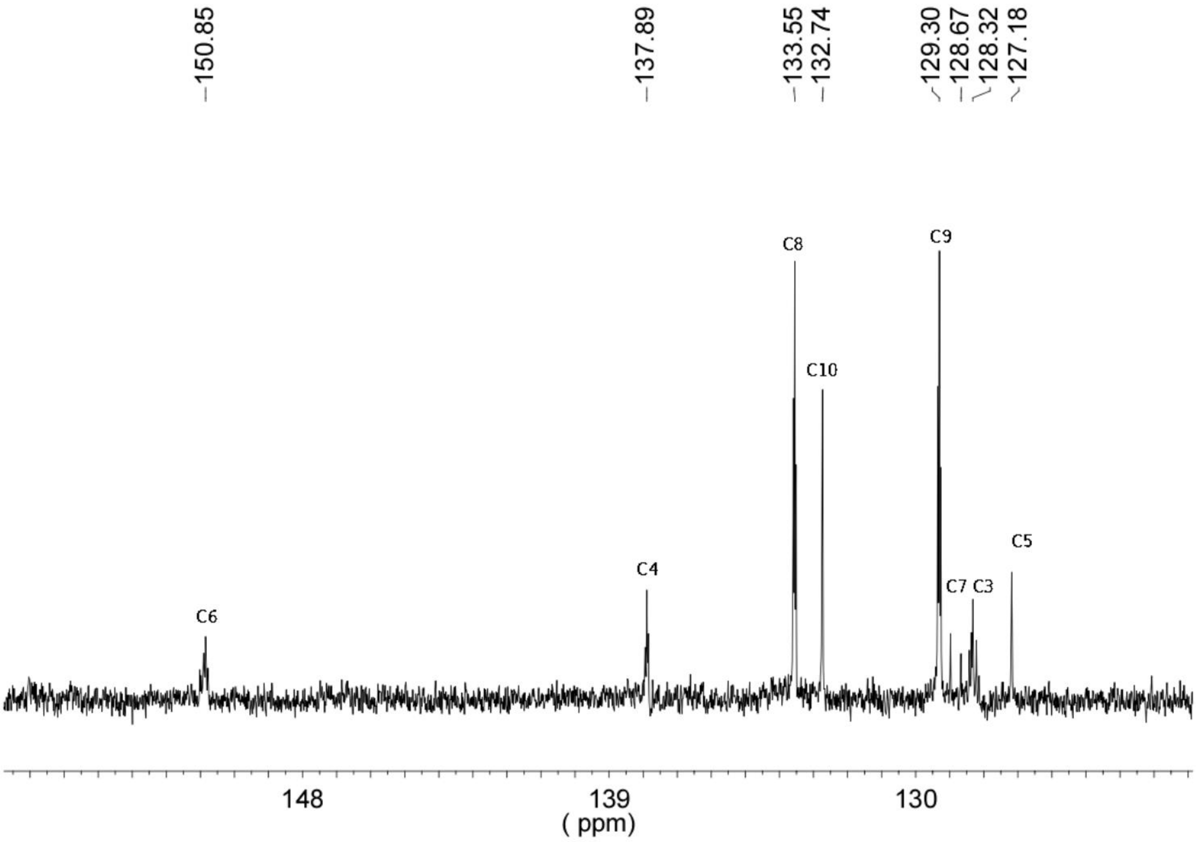

Figure S-7. $^{13}C\{^1H\}$ NMR (CCl_2D_2, 75 MHz, [ppm]) of [$(CuI)_2(CDP(Py)_2)$] (**3**).

[(CuPPh$_3$)$_2$(CDP(Py)$_2$)](PF$_6$)$_2$ (4)

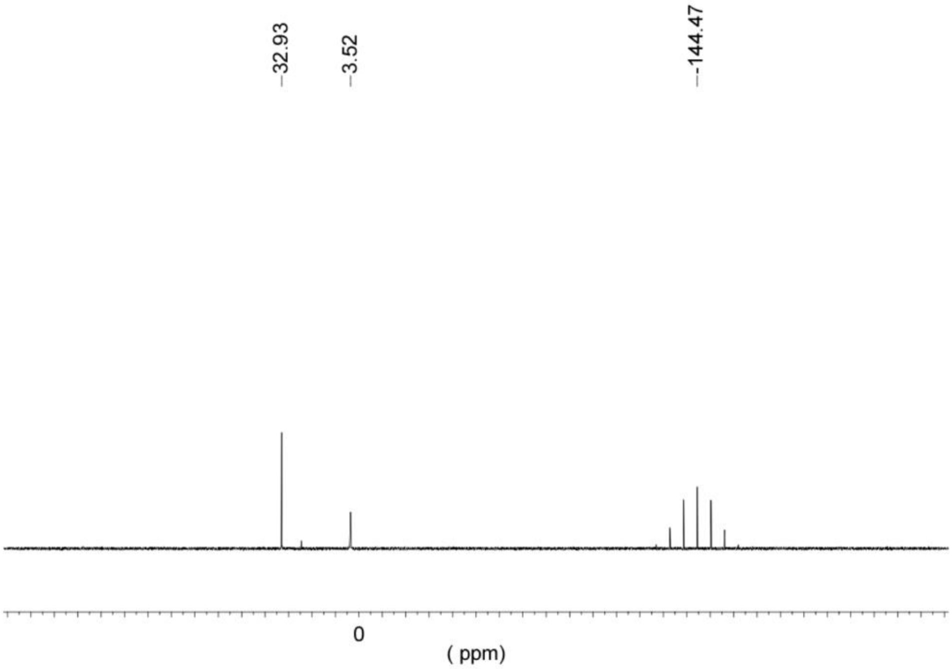

Figure S-8. ^{31}P{^{1}H} NMR (CCl$_2$D$_2$, 101 MHz, [ppm]) of [(CuPPh$_3$)$_2$(CDP(Py)$_2$)](PF$_6$)$_2$ (**4**).

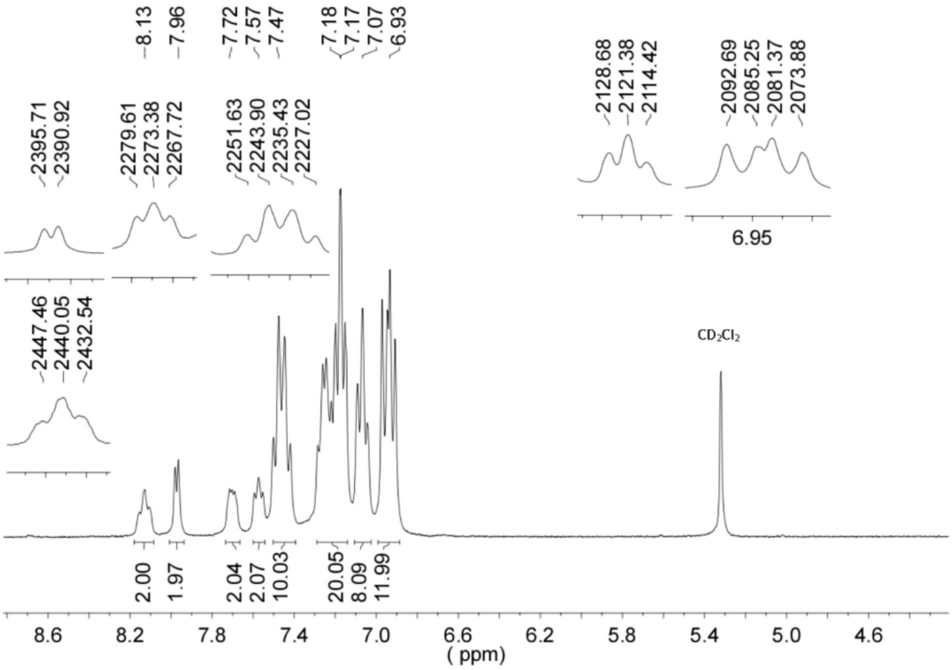

Figure S-9. ^{1}H NMR (CCl$_2$D$_2$, 300 MHz, [ppm]) of [(CuPPh$_3$)$_2$(CDP(Py)$_2$)](PF$_6$)$_2$ (**4**).

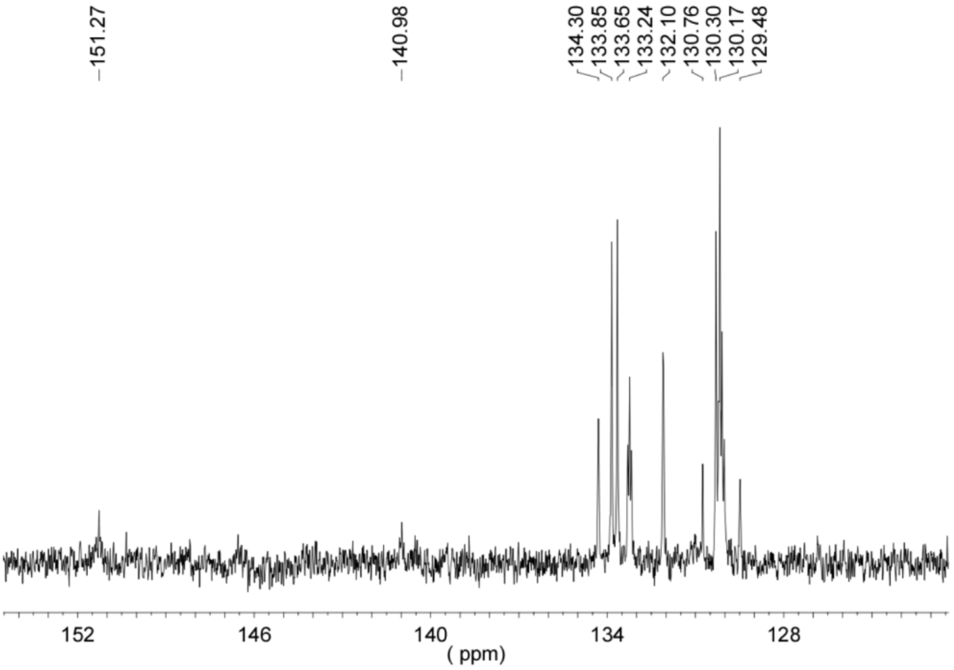

Figure S-10. $^{13}C\{^1H\}$ NMR (CCl_2D_2, 75 MHz, [ppm]) of $[(CuPPh_3)_2(CDP(Py)_2)](PF_6)_2$ (**4**).

$[(Cu(PPhOMe)_3)_2(CDP(Py)_2)](PF_6)_2$ (5)

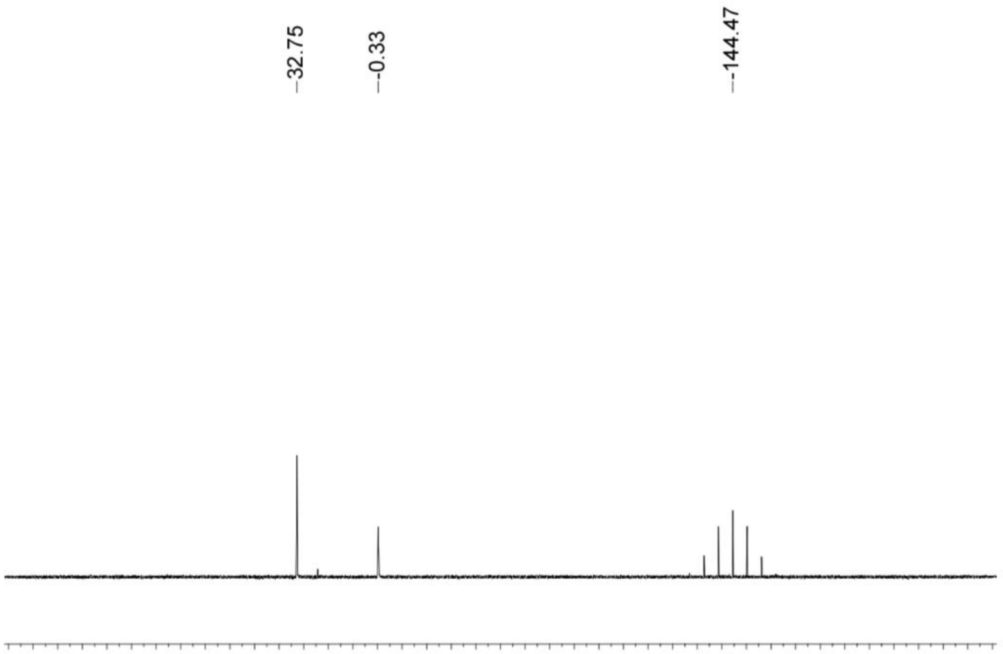

Figure S-11. $^{31}P\{^1H\}$ NMR (CCl_2D_2, 101 MHz, [ppm]) of $[(Cu(PPhOMe)_3)_2(CDP(Py)_2)](PF_6)_2$ (**5**).

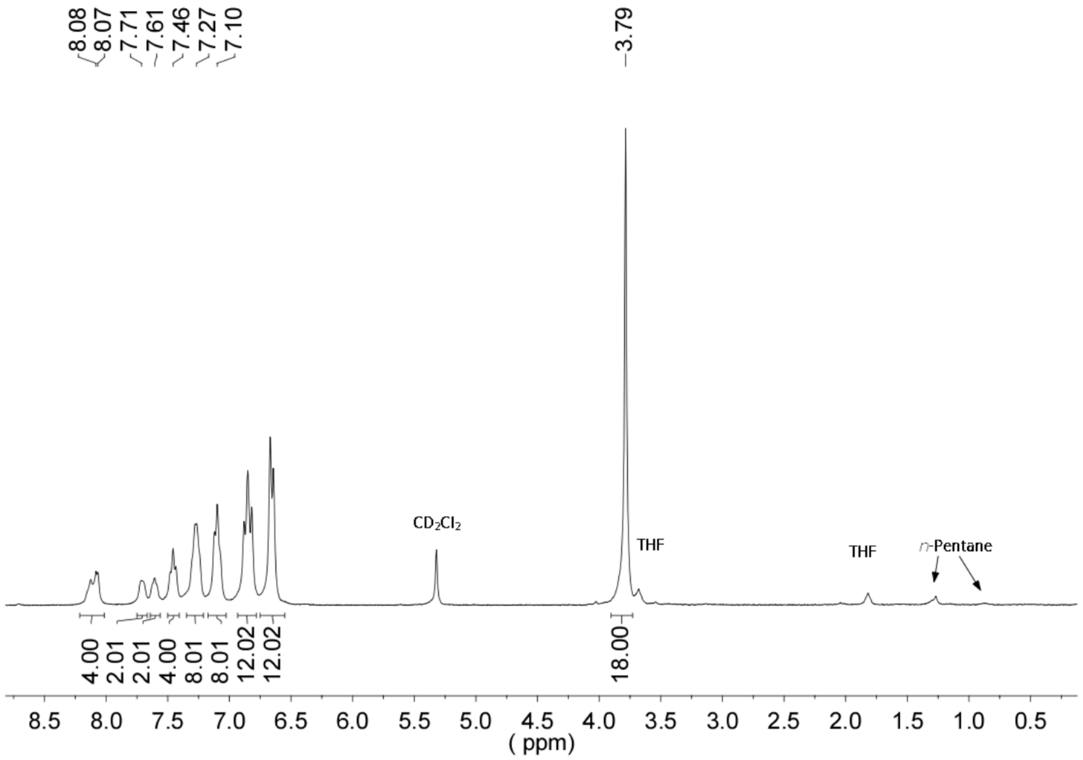

Figure S-12. ^{1}H NMR (CCl_2D_2, 300 MHz, [ppm]) of [(Cu(PPhOMe)$_3$)$_2$(CDP(Py)$_2$)](PF$_6$)$_2$ (**5**).

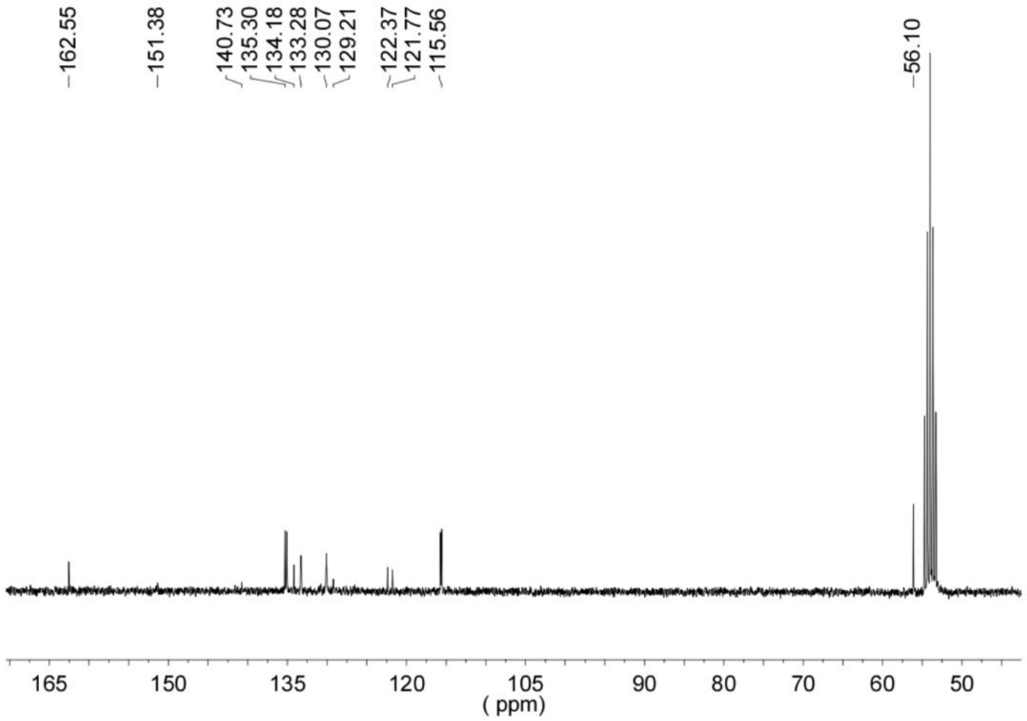

Figure S-13. $^{13}C\{^{1}H\}$ NMR (CCl_2D_2, 75 MHz, [ppm]) of [(Cu(PPhOMe)$_3$)$_2$(CDP(Py)$_2$)](PF$_6$)$_2$ (**5**).

$[Cu_2(DPEPhos)(CDP(Py)_2)](PF_6)_2$ (6)

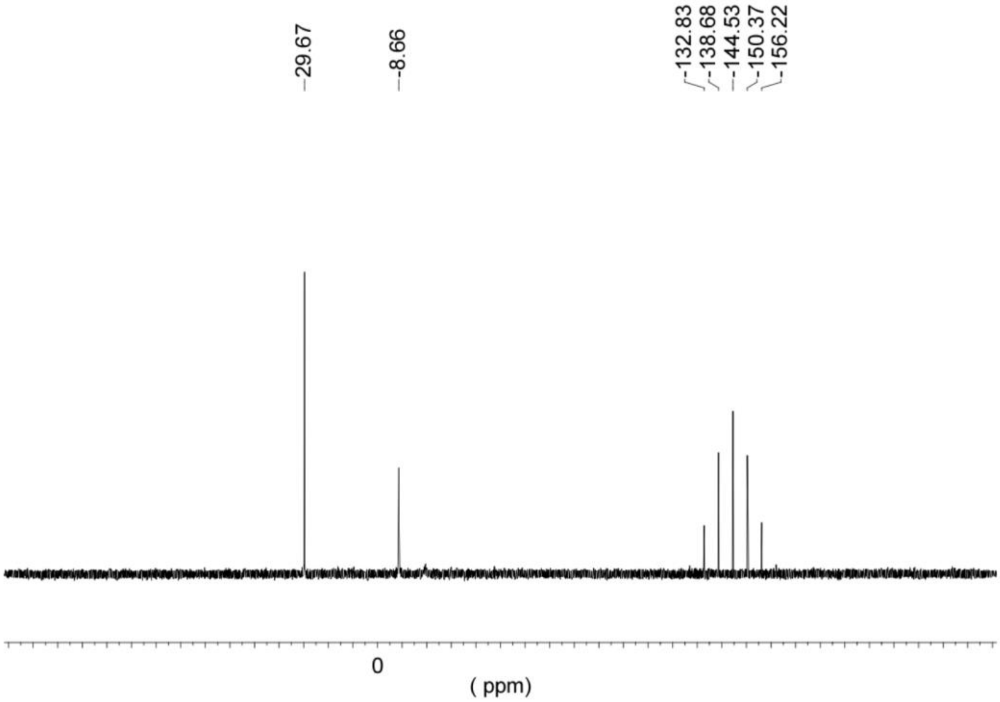

Figure S-14. $^{31}P\{^1H\}$ NMR (CCl_2D_2, 101 MHz, [ppm]) of $[Cu_2(DPEPhos)(CDP(Py)_2)](PF_6)_2$ (**6**).

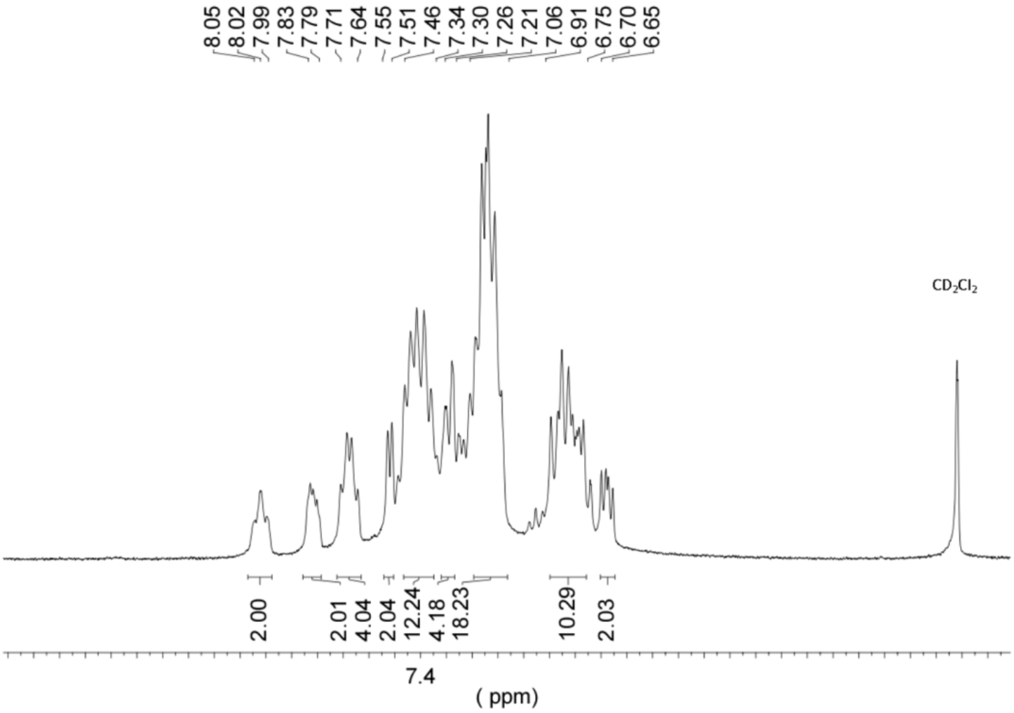

Figure S-15. ^1H NMR (CCl_2D_2, 300 MHz, [ppm]) of $[Cu_2(DPEPhos)(CDP(Py)_2)](PF_6)_2$ (**6**).

[Cu_2(XantPhos)(CDP(Py)$_2$)](PF_6)$_2$ (7)

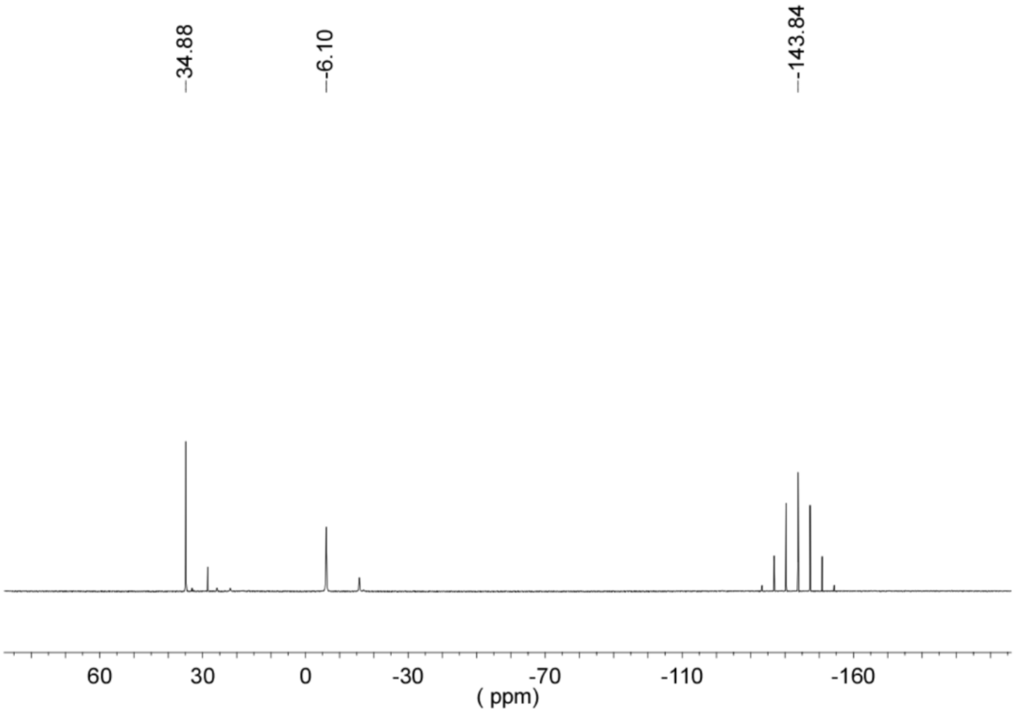

Figure S-16. $^{31}P\{^1H\}$ NMR (CCl_2D_2, 101 MHz, [ppm]) of [Cu_2(XantPhos)(CDP(Py)$_2$)](PF_6)$_2$ (**7**).

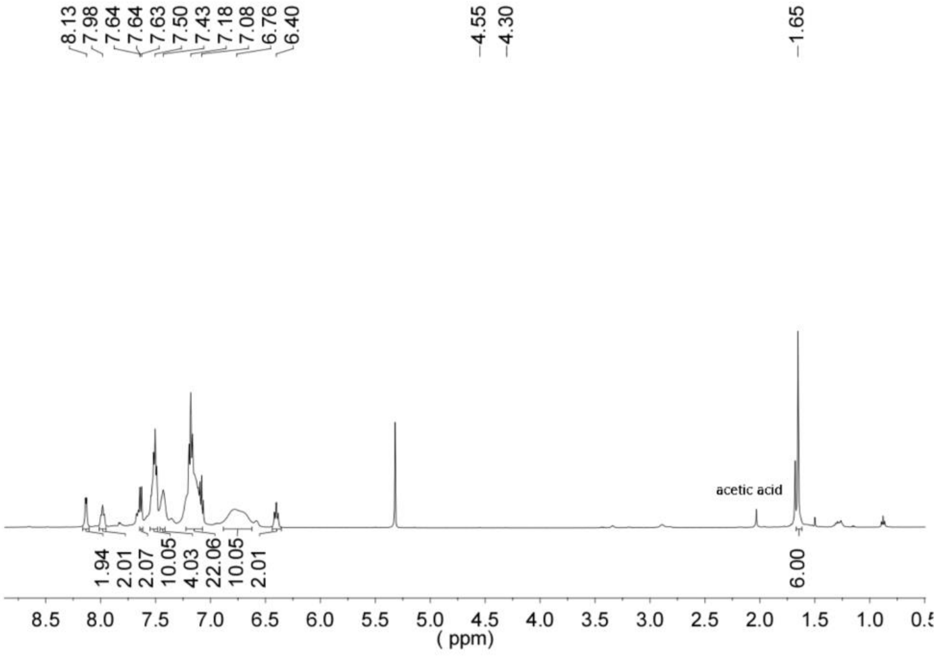

Figure S-17. ^1H NMR (CCl_2D_2, 300 MHz, [ppm]) of [Cu_2(XantPhos)(CDP(Py)$_2$)](PF_6)$_2$ (**7**).

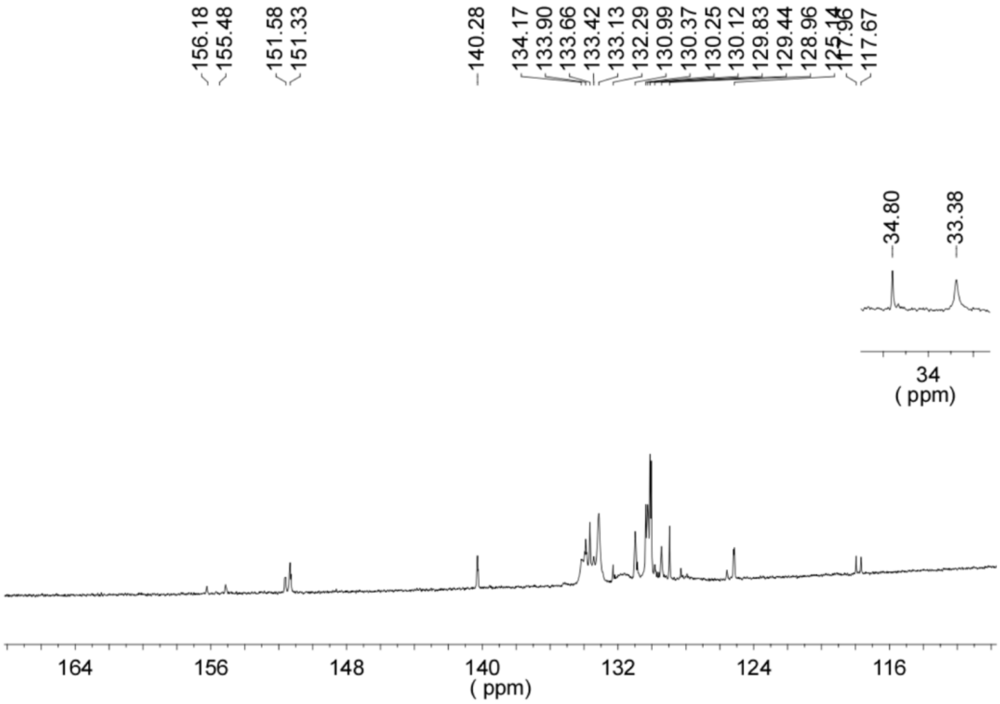

Figure S-18. $^{13}C\{^{1}H\}$ NMR (CCl_2D_2, 75 MHz, [ppm]) of $[Cu_2(XantPhos)(CDP(Py)_2)](PF_6)_2$ (**7**).

$[Cu_2(dppf)(CDP(Py)_2)](PF_6)_2$ (8)

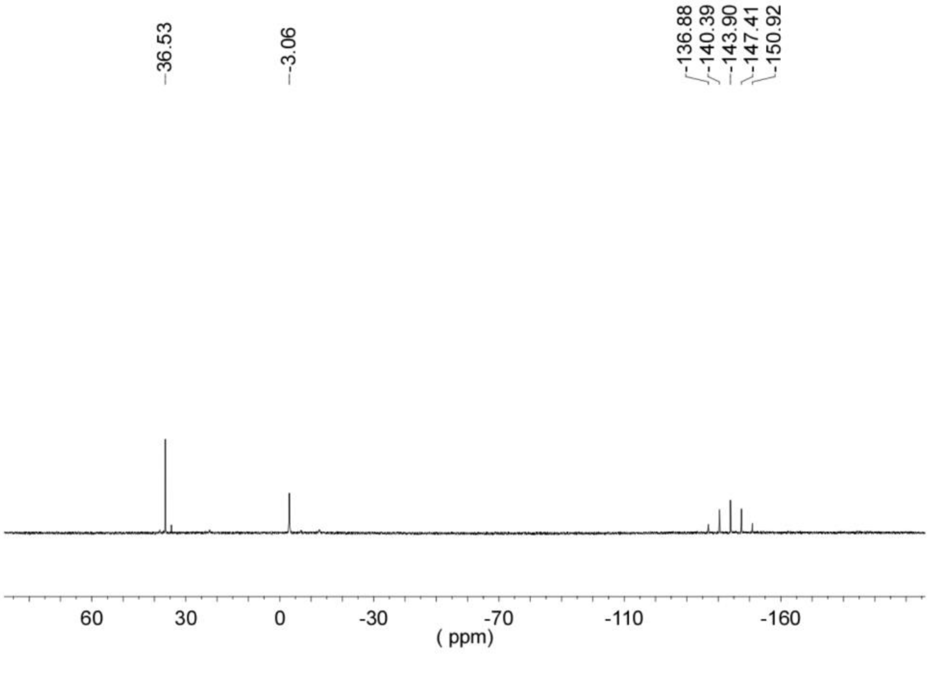

Figure S-19. $^{31}P\{^{1}H\}$ NMR (CCl_2D_2, 101 MHz, [ppm]) of $[Cu_2(dppf)(CDP(Py)_2)](PF_6)_2$ (**8**).

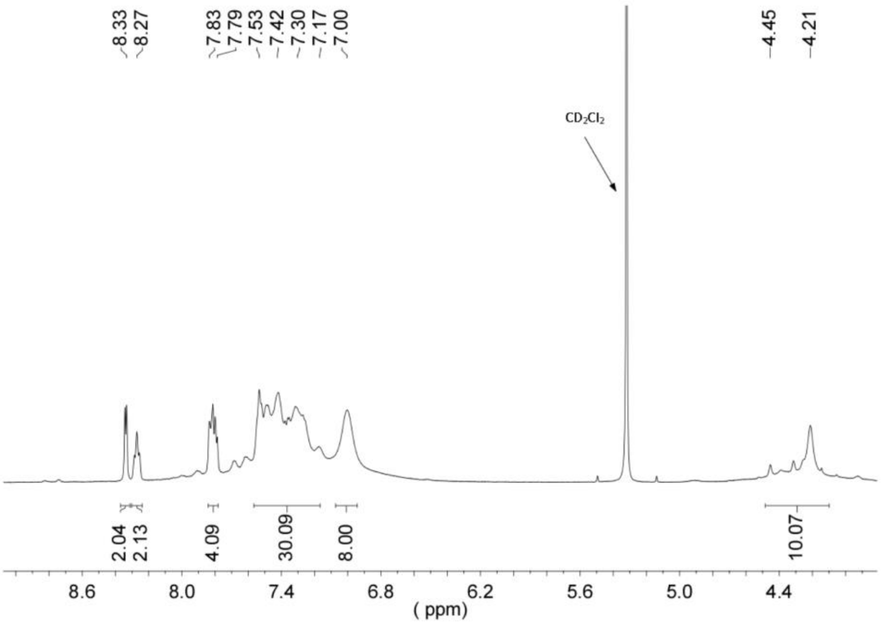

Figure S-20. ^{1}H NMR (CCl_2D_2, 300 MHz, [ppm]) of $[Cu_2(dppf)(CDP(Py)_2)](PF_6)_2$ (**8**).

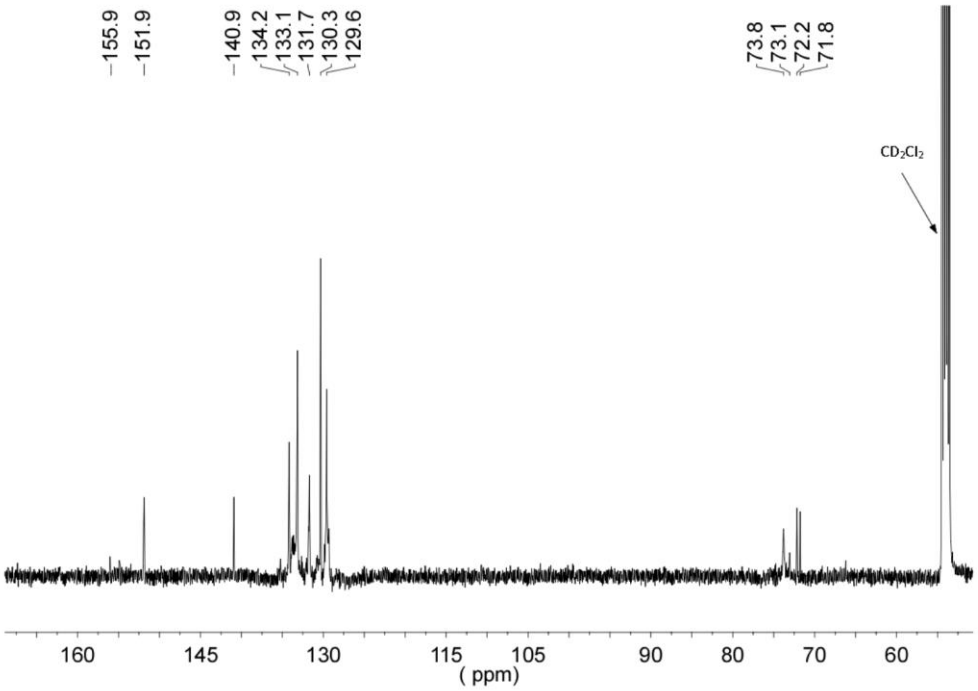

Figure S-21. $^{13}C\{^{1}H\}$ NMR (CCl_2D_2, 75 MHz, [ppm]) of $[Cu_2(dppf)(CDP(Py)_2)](PF_6)_2$ (**8**).

[(CuCarb)$_2$(CDP(Py)$_2$)] (11)

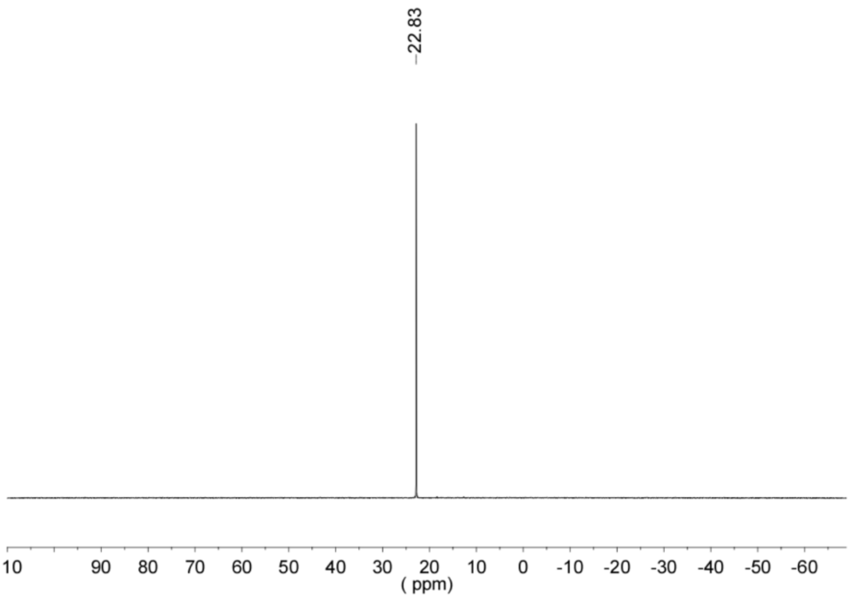

Figure S-22. $^{31}P\{^1H\}$ NMR (CCl_2D_2, 101 MHz, [ppm]) of [(CuCarb)$_2$(CDP(Py)$_2$)] (**11**).

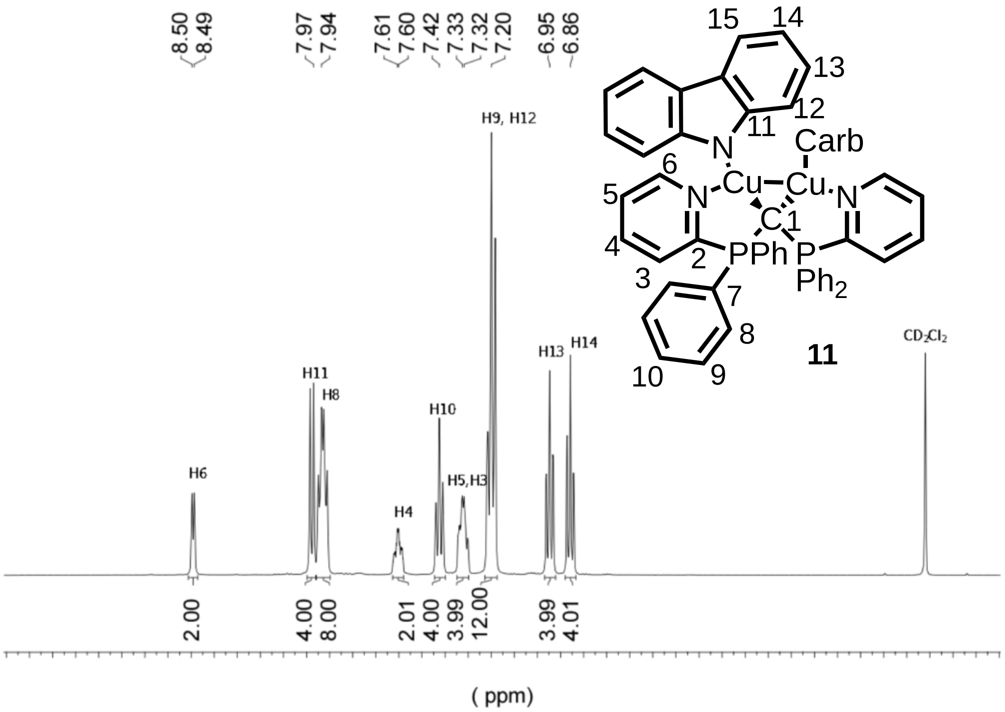

Figure S-23. ^1H NMR (CCl_2D_2, 300 MHz, [ppm]) of [(CuCarb)$_2$(CDP(Py)$_2$)] (**11**).

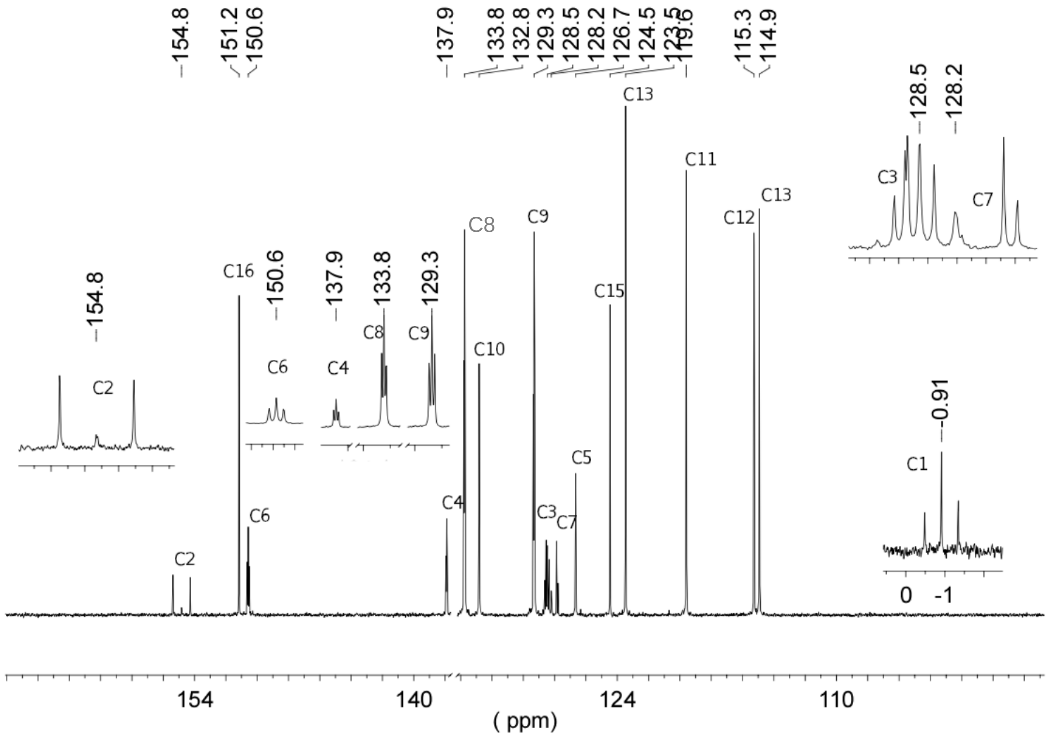

Figure S-24. $^{13}C\{^1H\}$ NMR (CCl_2D_2, 75 MHz, [ppm]) of [$(CuCarb)_2(CDP(Py)_2)$] (**11**).

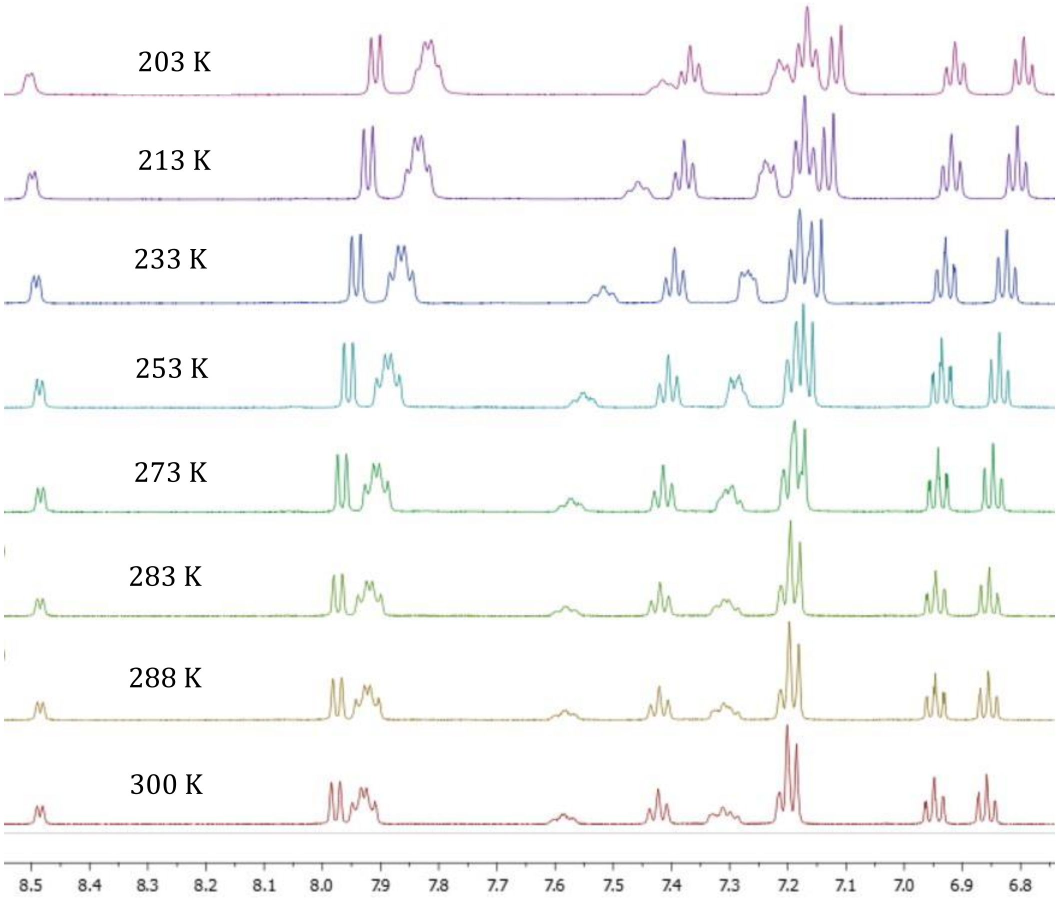

Figure S-25. ^1H NMR (CCl_2D_2, 300 MHz, [ppm]) of [$(CuCarb)_2(CDP(Py)_2)$] (**11**) at temperatures between 203-300 K.

[(CuS(C_6F_5))$_2$(CDP(Py)$_2$)] (9)

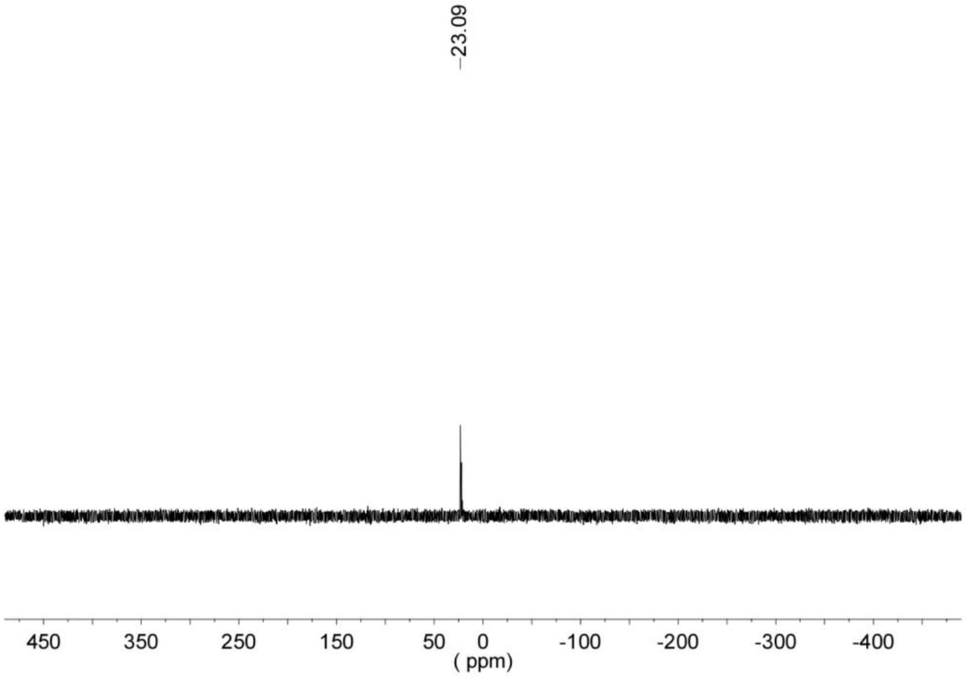

Figure S-26. $^{31}P\{^{1}H\}$ NMR (CCl_2D_2, 101 MHz, [ppm]) of [(CuS(C_6F_5))$_2$(CDP(Py)$_2$)] (**9**).

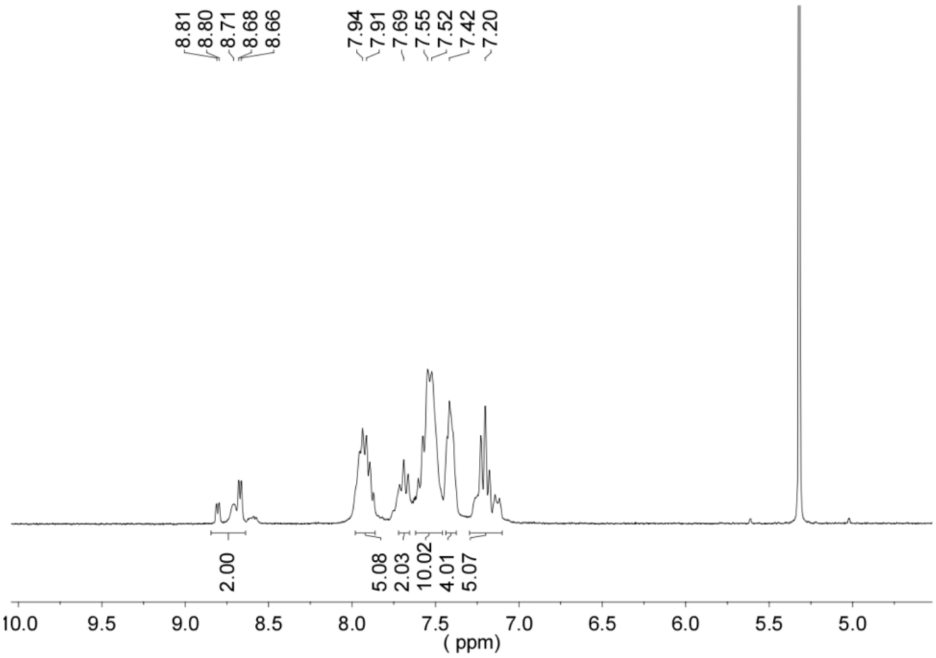

Figure S-27. ^{1}H NMR (CCl_2D_2, 300 MHz, [ppm]) of [(CuS(C_6F_5))$_2$(CDP(Py)$_2$)] (**9**).

(CDP(CH2PPh2)2 (13)

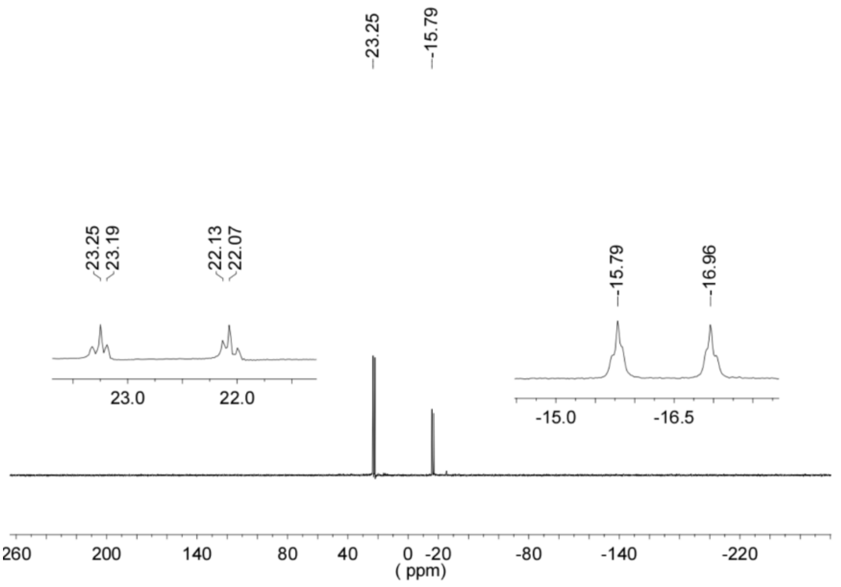

Figure S-28. $^{31}P\{^1H\}$ NMR (Toluol, 101 MHz, [ppm]) of (CDP(CH2PPh2)2 (**13**).

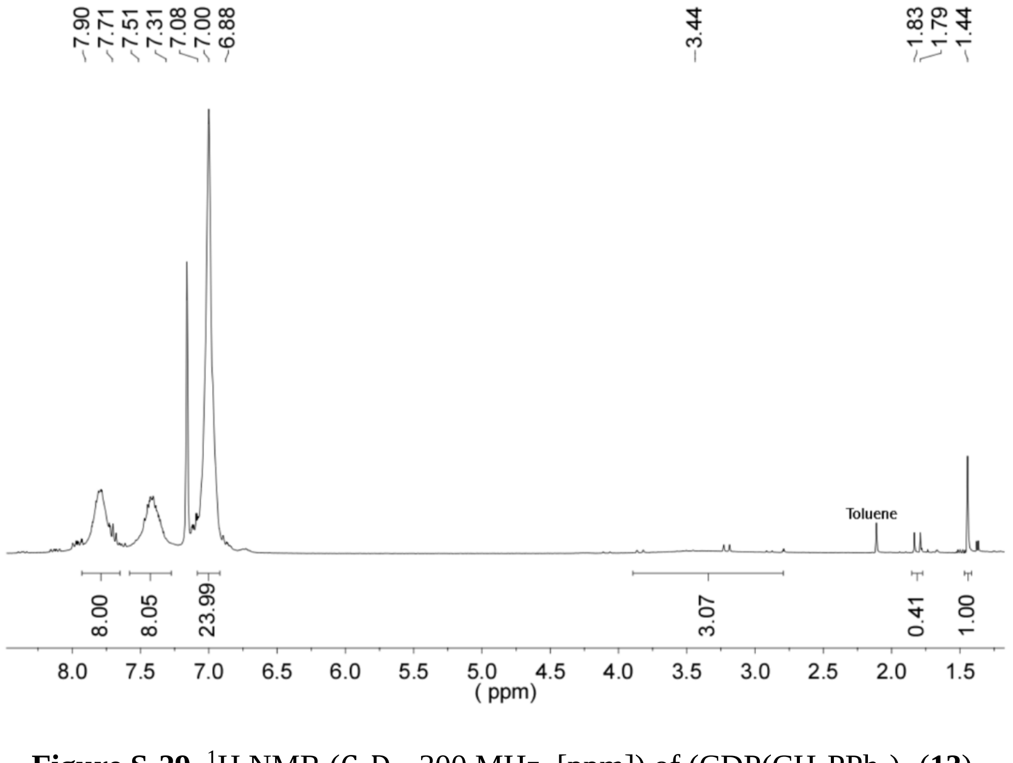

Figure S-29. ^1H NMR (C_6D_6, 300 MHz, [ppm]) of (CDP(CH2PPh2)2 (**13**).

[(CuCl)$_2$(CDP(CH$_2$PPh$_2$)$_2$] (14)

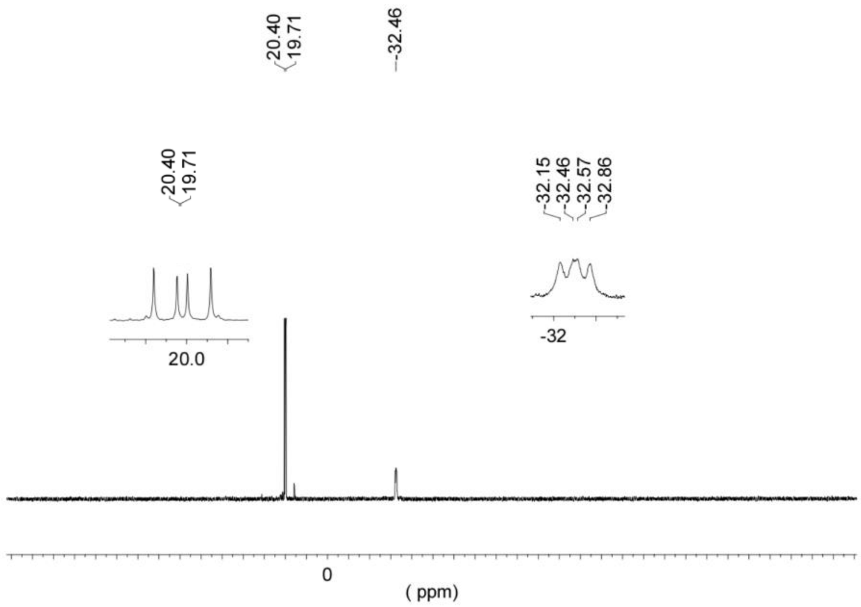

Figure S-30. ^{31}P{^{1}H} NMR (CCl_2D_2, 101 MHz, [ppm]) of [(CuCl)$_2$(CDP(CH$_2$PPh$_2$)$_2$] (**14**).

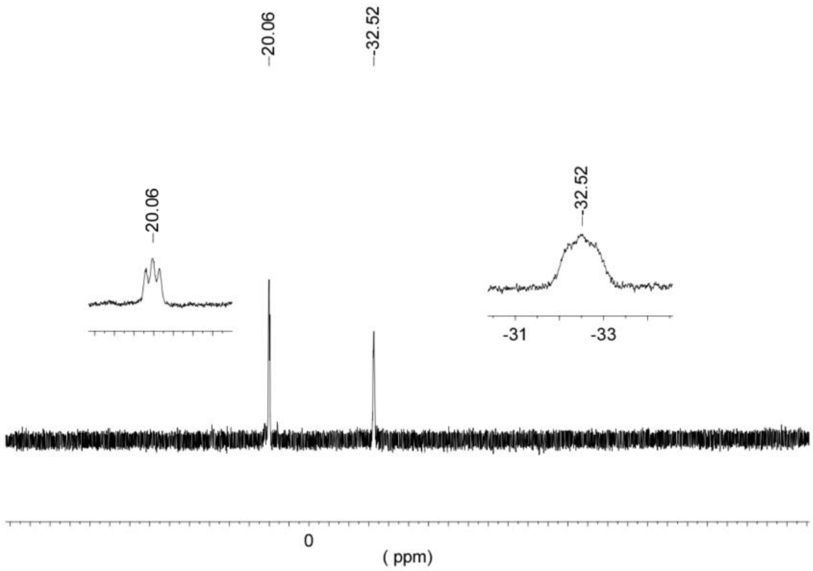

Figure S-31. ^{31}P{^{1}H} NMR (CCl_2D_2, 101 MHz, [ppm]) of [(CuCl)$_2$(CDP(CH$_2$PPh$_2$)$_2$] (**14**).

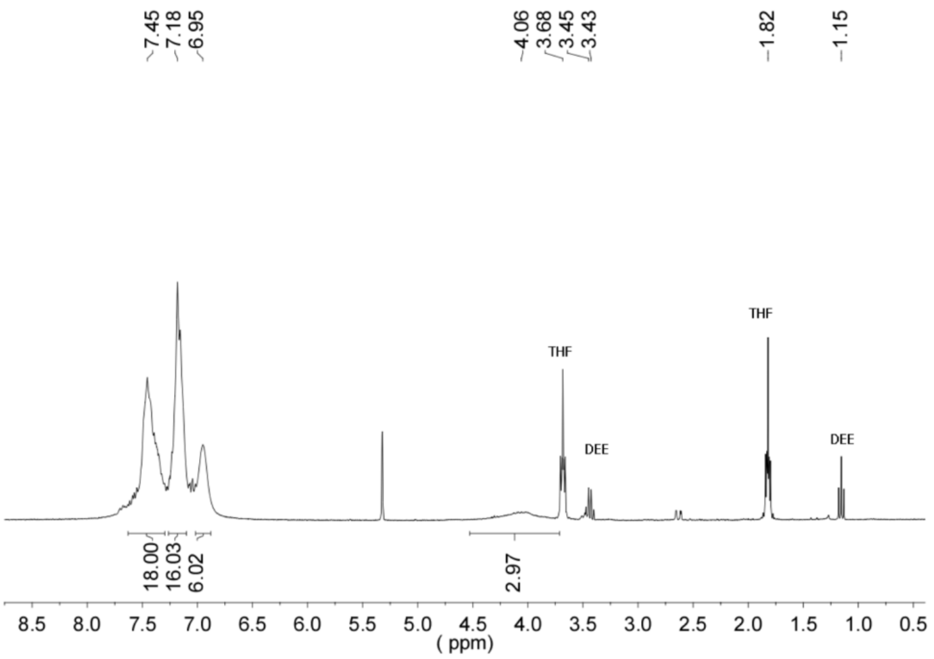

Figure S-32. ^{1}H NMR (CCl_2D_2, 300 MHz, [ppm]) of [$(CuCl)_2(CDP(CH_2PPh_2)_2$] (**14**).

[$(CuI)_2(CDP(CH_2PPh_2)_2$] (15)

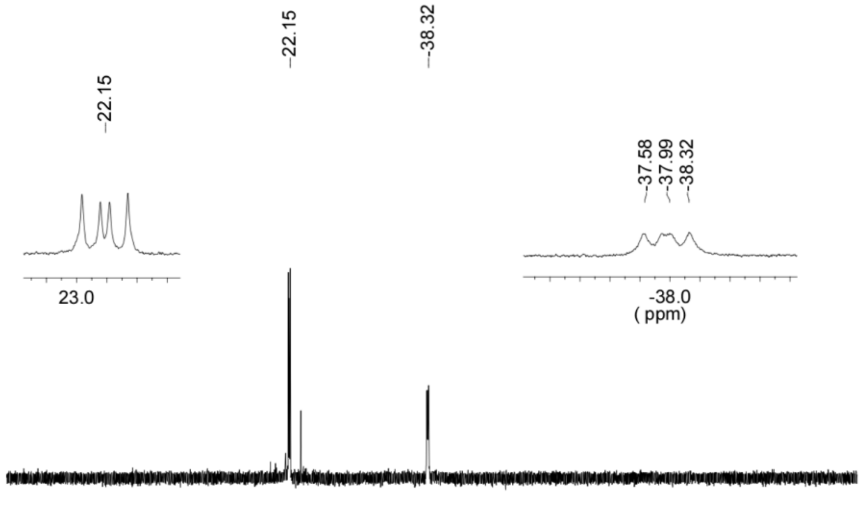

Figure S-33. $^{31}P\{^{1}H\}$ NMR (CCl_2D_2, 101 MHz, [ppm]) of [$(CuI)_2(CDP(CH_2PPh_2)_2$] (**15**).

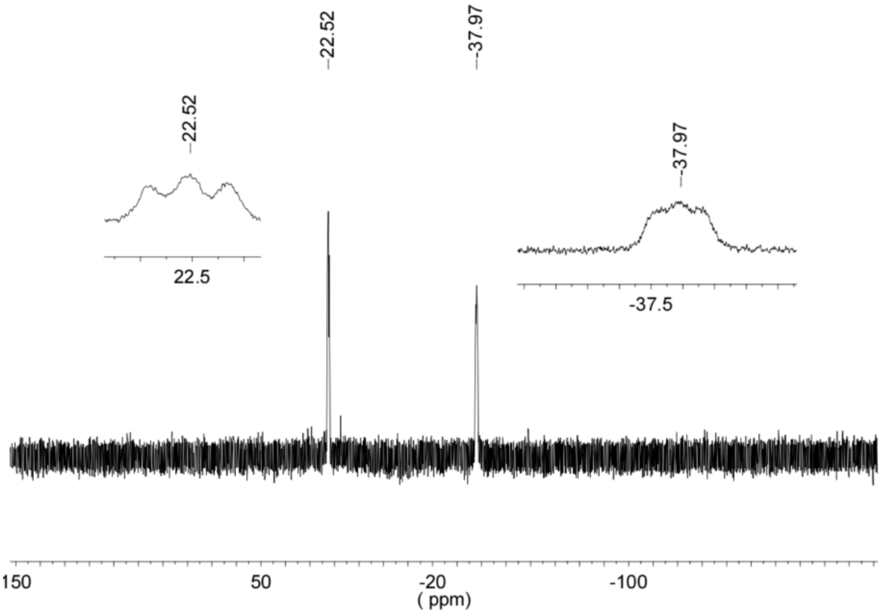

Figure S-34. $^{31}P\{^{1}H\}$ NMR (CCl_2D_2, 101 MHz, [ppm]) of $[(CuI)_2(CDP(CH_2PPh_2)_2]$ (**15**).

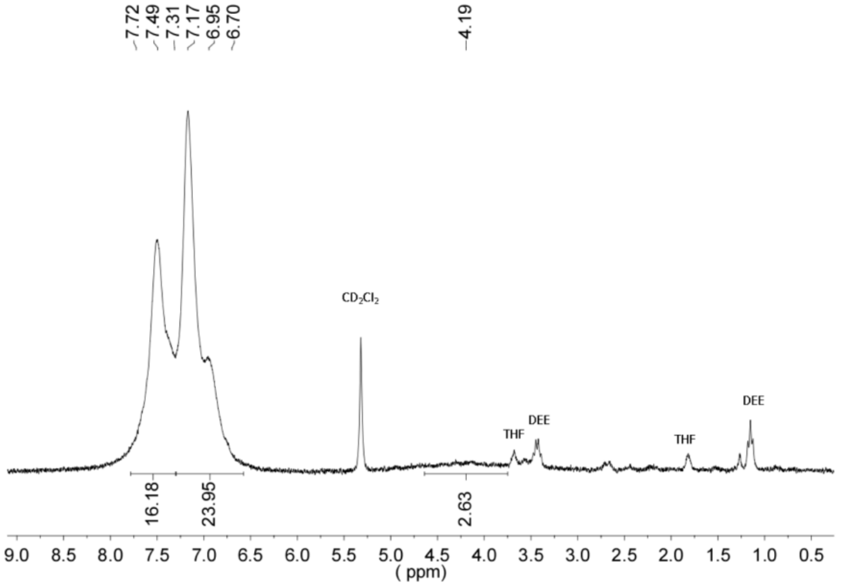

Figure S-35. ^{1}H NMR (CCl_2D_2, 300 MHz, [ppm]) of $[(CuI)_2(CDP(CH_2PPh_2)_2]$ (**15**).

[(CuSPh)$_2$(CDP(CH$_2$PPh$_2$)$_2$] (16)

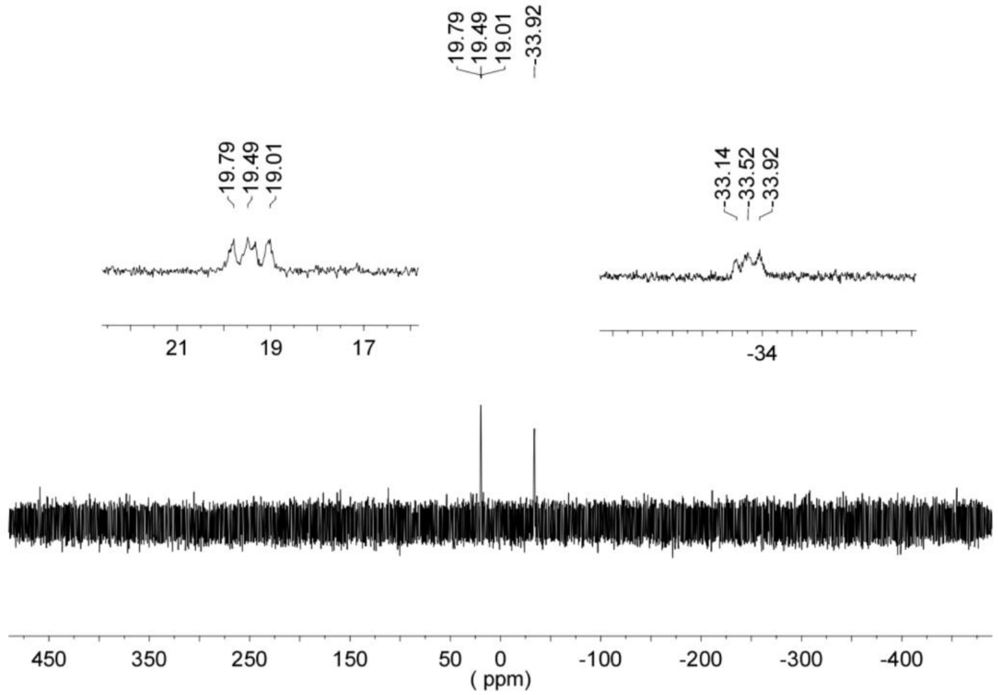

Figure S-36. $^{31}P\{^{1}H\}$ NMR (CCl_2D_2, 101 MHz, [ppm]) of [(CuSPh)$_2$(CDP(CH$_2$PPh$_2$)$_2$] (**16**).

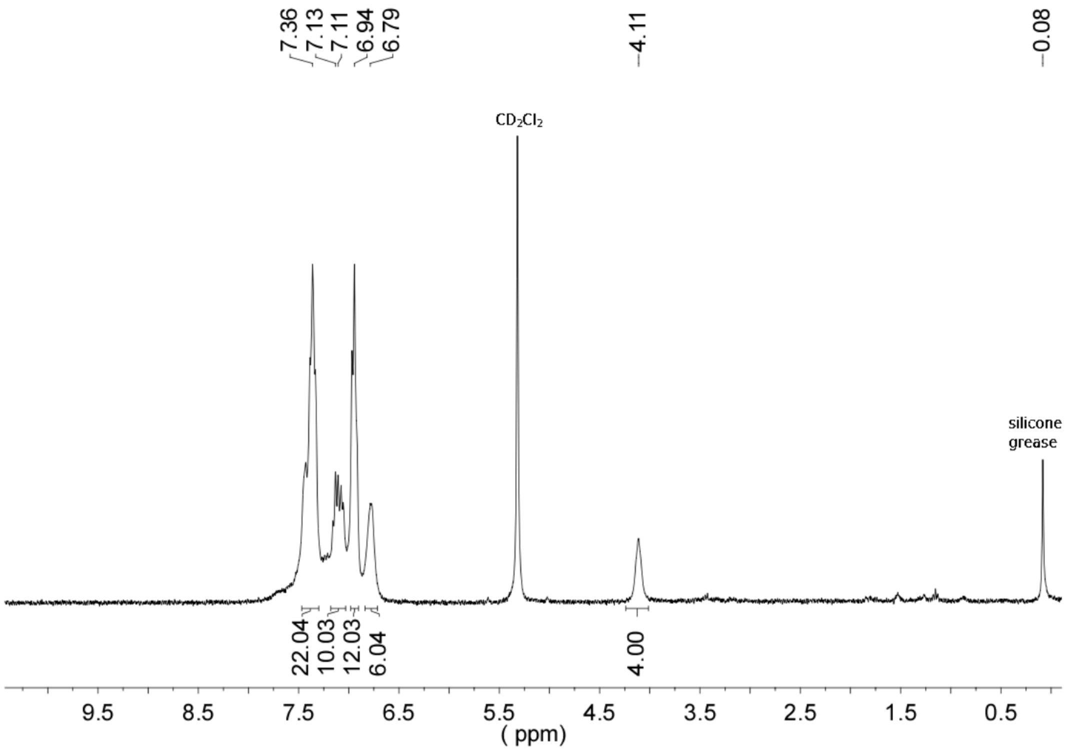

Figure S-37. ^{1}H NMR (CCl_2D_2, 300 MHz, [ppm]) of [(CuSPh)$_2$(CDP(CH$_2$PPh$_2$)$_2$] (**16**).

$[CuCl(CDPH(CH_2PPh_2)_2]PF_6$ (17)

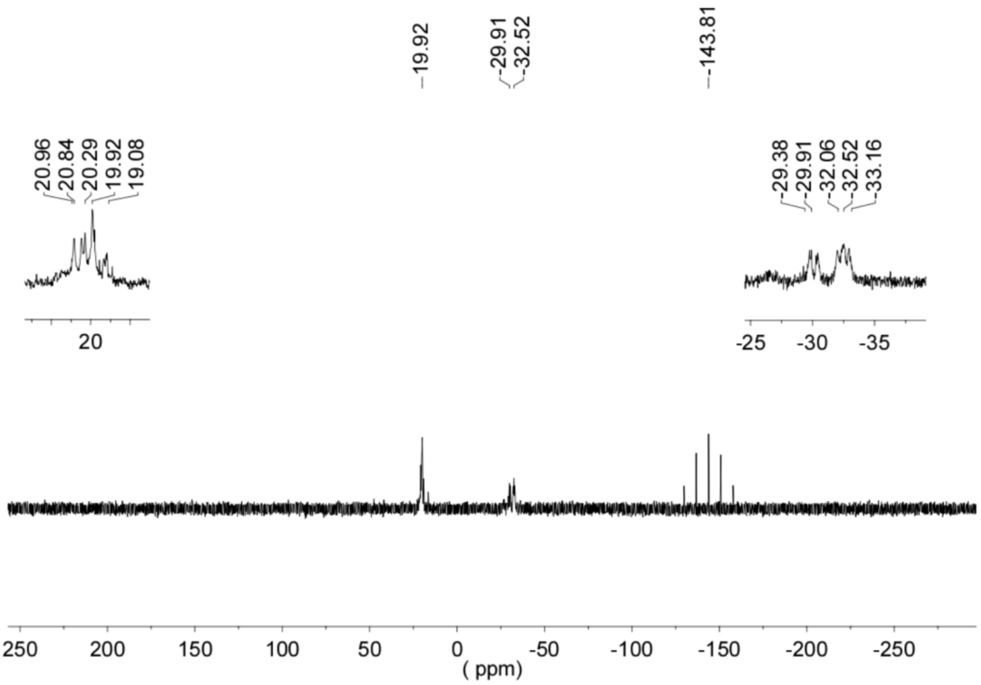

Figure S-38. $^{31}P\{^1H\}$ NMR (CCl_2D_2, 101 MHz, [ppm]) of $[CuCl(CDPH(CH_2PPh_2)_2]PF_6$ (**17**).

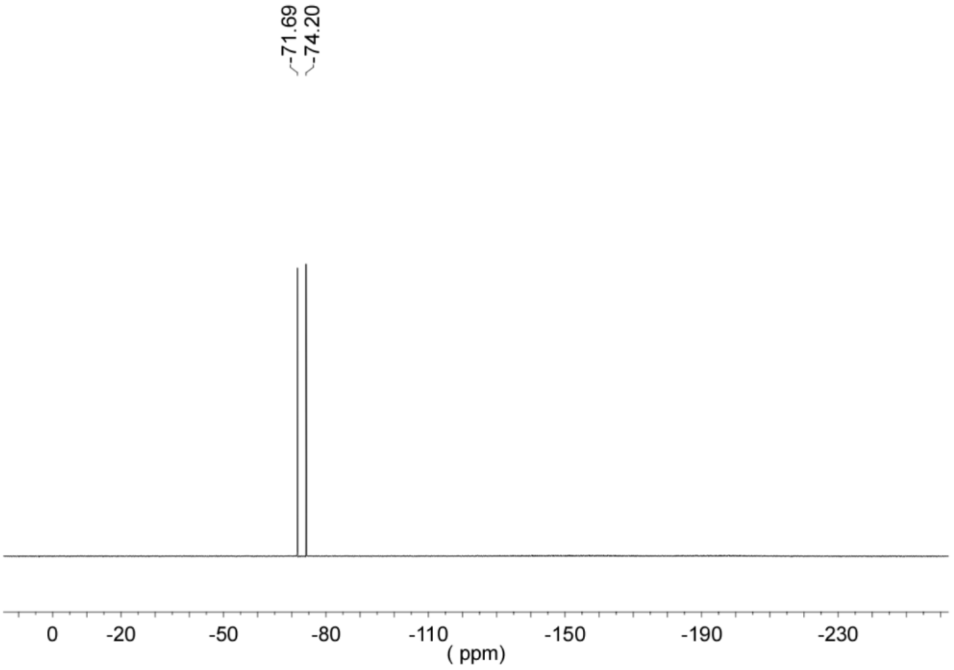

Figure S-39. ^{19}F NMR (CCl_2D_2, 282 MHz, [ppm]) of $[CuCl(CDPH(CH_2PPh_2)_2]PF_6$ (**17**).

[CuPPh$_3$(CH(PPh$_2$CHPPh$_2$)$_2$] (18)

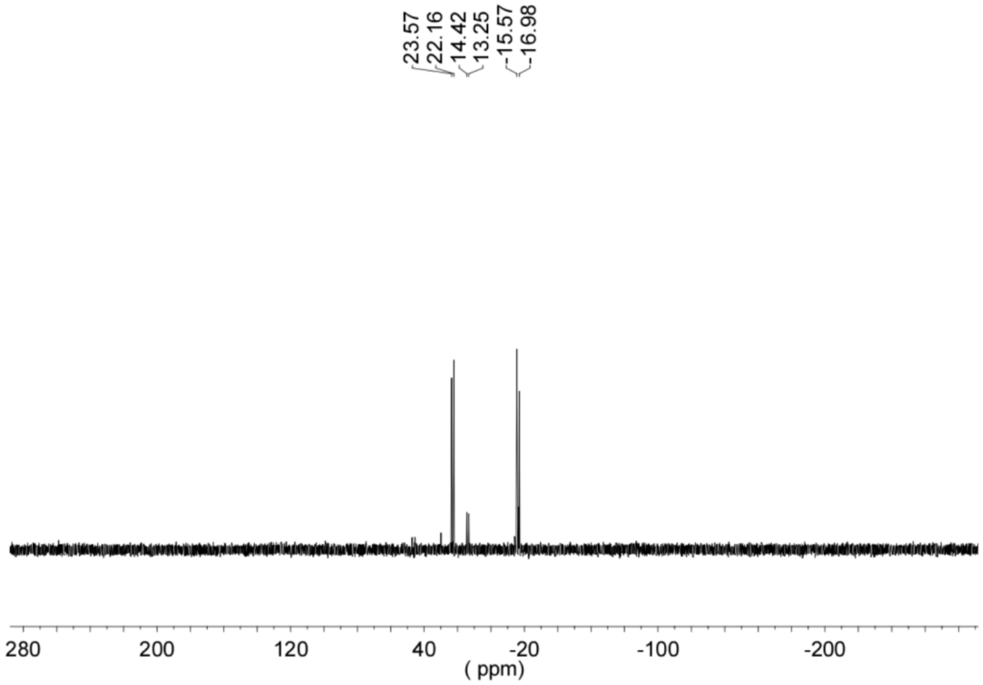

Figure S-40. $^{31}P\{^1H\}$ NMR (CCl_2D_2, 101 MHz, [ppm]) of [CuPPh$_3$(CH(PPh$_2$CHPPh$_2$)$_2$] (**18**).

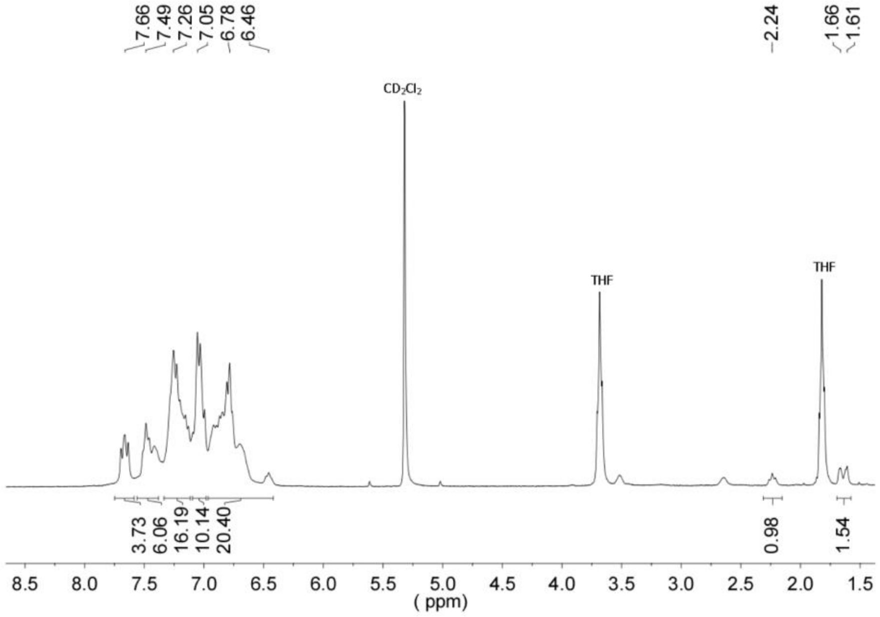

Figure S-41. ^1H NMR (CCl_2D_2, 300 MHz, [ppm]) of [CuPPh$_3$(CH(PPh$_2$CHPPh$_2$)$_2$] (**18**).

IR Spectroscopy

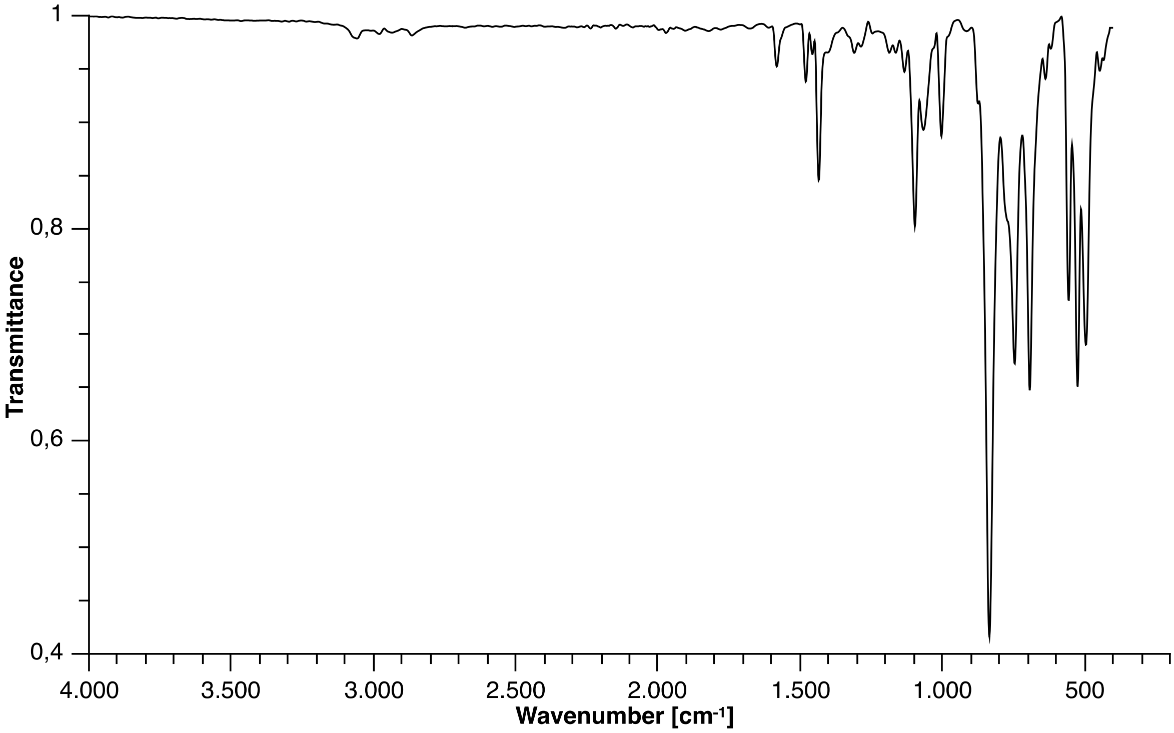

Figure S-42. Experimental IR Spectrum of $[(CuPPh_3)_2(CDP(Py)_2)](PF_6)_2$ (**4**).

Crystallographic Data

[(CuCl)$_2$(CDP(Py)$_2$)] (2)
MKML27 | CCDC: 2017394

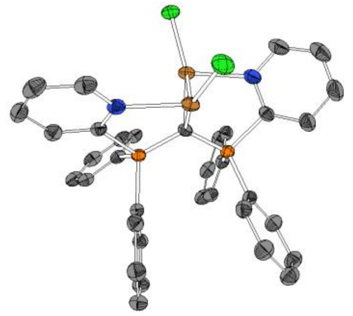

Crystal data

$C_{35}H_{28}Cl_2Cu_2N_2P_2$	Block	Yellow
Triclinic	$P\bar{1}$	Z = 4
a = 10.5074(2) Å	b = 15.9277(3) Å	c = 19.8575(4) Å
α = 82.0400(10) °	β = 80.765(2) °	γ = 80.376(2) °
V = 3212.73(11) Å^3	μ = 0.465 mm^{-1}	*F*(000) = 1496
0.182 x 0.151 x 0.094 mm^3	*M* = 0.73651 kg·mol^{-1}	D_{calc} = 1.523 g·cm^{-3}
N_{ref} = 43369	Θ_{min} = 4.3 °	Θ_{max} = 75.7 °

Data collection

X-Area Pilatus3_SV 1.31.127.0 (Stoe, 2016)		λ = 1.54178 Å
Θ_{min} = 4.310 °	Θ_{max} = 66.593 °	*T* = 100(2) K
h = 12 → –7	*k* = 18 → –18	*l* = 23→ –23

Structure solution / refinement

N_{ref} measured = 43764	N_{ref} independent = 11181	R_{Int} = 0.0277
Semi-empirical from equivalents	T_{max} = 0.9848; T_{min} = 0.4528	Full-matrix least-squares on F^2
$C_{25.00\,°}$ = 98.6%	N_{ref} used = 11181	N_{ref} $I>2\sigma(I)$ = 9821
$\Delta\rho_{max}$ = 0.507 eÅ^{-3}	$\Delta\rho_{max}$ = –0.614 eÅ^{-3}	
N_{ref} = 11181	$N_{restraints}$ = 6	$N_{parameters}$ = 795
$R_1(I>2\sigma(I))$ = 0.0337	$\omega R_2(I)$ = 0.0864	$GooF(F^2)$ = 1.045

[(CuCl)$_2$(CDP(Py)$_2$)] (3)
MKP80 | CCDC: 2017392

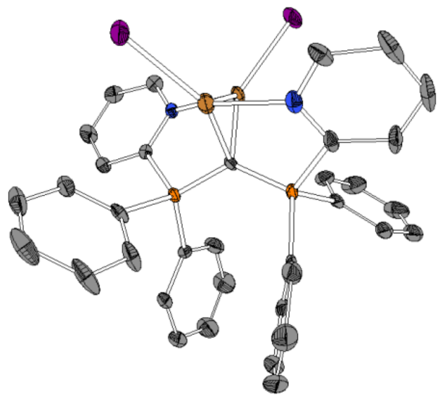

Crystal data

$C_{35}H_{28}I_2Cu_2N_2P_2$	Block	Orange
Triclinic	$P\bar{1}$	Z = 2
a = 12.0153(5) Å	b = 12.8465(5) Å	c = 13.5785(6) Å
α = 109.8360(10) °	β = 94.1550(10) °	γ = 93.9560(10) °
V = 1956.56(14) Å^3	μ = 2.770 mm^{-1}	*F*(000) = 892
0.140 x 0.130 x 0.110 mm^3	*M* = 0.91943 kg·mol^{-1}	D_{calc} = 1.561 g·cm^{-3}
N_{ref} = 9708	Θ_{min} = 2.5 °	Θ_{max} = 26.6 °

Data collection

D8 Quest (Bruker AXS Inc.)	*T* = 100(2) K	λ = 0.71073 Å
Θ_{min} = 2.282 °	Θ_{max} = 25.027 °	
h = 14 → –14	*k* = 15 → –15	*l* = 16→ –15

Structure solution / refinement

N_{ref} measured = 30066	N_{ref} independent = 6918	R_{Int} = 0.0618
Semi-empirical from equivalents	T_{max} = 0.7452; T_{min} = 0.6292	Full-matrix least-squares on F^2
$C_{25.00\,°}$ = 99.9%	N_{ref} used = 6918	N_{ref} $I>2\sigma(I)$ = 5356
$\Delta\rho_{max}$ = 0.871 eÅ^{-3}	$\Delta\rho_{max}$ = –0.884 eÅ^{-3}	
N_{ref} = 6918	$N_{restraints}$ = 36	$N_{parameters}$ = 388
$R_1(I>2\sigma(I))$ = 0.0432	$\omega R_2(I)$ = 0.0956	$GooF(F^2)$ = 1.045

[$(CuPPh_3)_2(CDP(Py)_2)](PF_6)_2$ (4)
MKP82| CCDC: 2017399

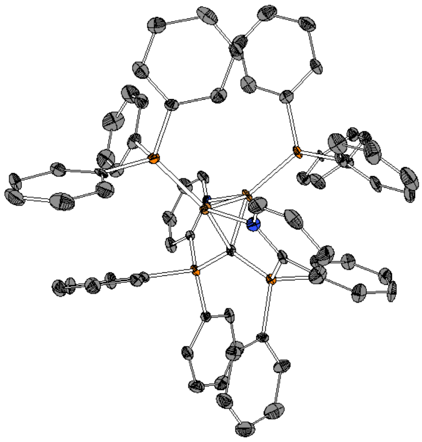

Crystal data

$C_{151}H_{129}Cl_{18}Cu_4F_{24}N_4P_{12}$	Block	Yellow
Monoclinic	*Pn*	Z = 2
a = 16.7727(6) Å	b = 22.4945(8) Å	c = 21.1551(7) Å
α = 90 °	β = 94.708(3) °	γ = 90 °
V = 7954.8(5) $Å^3$	μ = 1.030 mm^{-1}	$F(000)$ = 3762
0.722 x 0.222 x 0.198 mm^3	M = 3.71953 kg·mol^{-1}	D_{calc} = 1.553 g·cm^{-3}
N_{ref} = 9066	Θ_{min} = 2.3 °	Θ_{max} = 27.8 °

Data collection

D8 Quest (Bruker AXS Inc.)	T = 100(2) K	λ = 0.71073 Å
Θ_{min} = 2.134 °	Θ_{max} = 25.026 °	
$h = 19 \rightarrow -19$	$k = 24 \rightarrow -26$	$l = 25 \rightarrow -25$

Structure solution / refinement

N_{ref} measured = 71983	N_{ref} independent = 27071	R_{Int} = 0.0904
Semi-empirical from equivalents	T_{max} = 0.7456; T_{min} = 0.4636	Full-matrix least-squares on F^2
$C_{25.00\,°}$ = 99.9%	N_{ref} used = 27071	N_{ref} $I>2\sigma(I)$ = 22422
$\Delta\rho_{max}$ = 0.910 e$Å^{-3}$	$\Delta\rho_{max}$ = –0.651 e$Å^{-3}$	
N_{ref} = 27071	$N_{restraints}$ = 972	$N_{parameters}$ = 1928
$R_1(I>2\sigma(I))$ = 0.0609	$\omega R_2(I)$ = 0.1560	$GooF(F^2)$ = 1.035

$[(Cu(PPhOMe)_3)_2(CDP(Py)_2)](PF_6)_2$ (5)
MKND122 | CCDC: 2017397

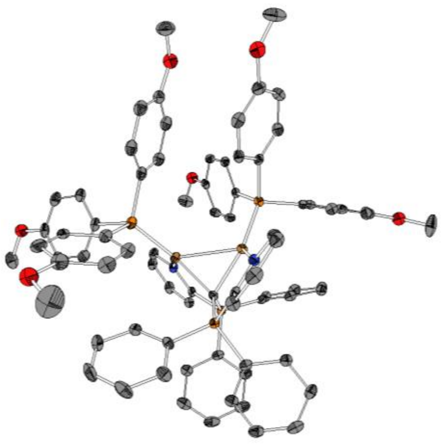

Crystal data

$C_{182}H_{196}Cu_4F_{24}N_4O_{19}P_{12}$	Block	Yellow
Triclinic	$P2_1/n$	Z = 2
a = 18.8255(11) Å	b = 24.2698(15) Å	c = 20.5171(12) Å
α = 90 °	β = 110.359(2) °	γ = 90 °
V = 8788.5(9) Å^3	μ = 0.678 mm^{-1}	F(000) = 884.2
0.220 x 0.181 x 0.133 mm^3	M = 3.82528 kg·mol^{-1}	D_{calc} = 1.446 g·cm^{-3}
N_{ref} = 99844	Θ_{min} = 2.3 °	Θ_{max} = 28.8 °

Data collection

D8 Quest (Bruker AXS Inc.)	T = 100(2) K	λ = 0.71073 Å
Θ_{min} = 2.195 °	Θ_{max} = 25.027 °	
$h = 22 \rightarrow -22$	$k = 28 \rightarrow -28$	$l = 24 \rightarrow -24$

Structure solution / refinement

N_{ref} measured = 123356	N_{ref} independent = 15519	R_{Int} = 0.0738
Semi-empirical from equivalents	T_{max} = 0.7458; T_{min} = 0.6659	Full-matrix least-squares on F^2
$C_{25.00\,°}$ = 99.4%	N_{ref} used = 15519	N_{ref} $I>2\sigma(I)$ = 12059
$\Delta\rho_{max}$ = 1.121 eÅ^{-3}	$\Delta\rho_{max}$ = –0.753 eÅ^{-3}	
N_{ref} = 15519	$N_{restraints}$ = 55	$N_{parameters}$ = 1176
$R_1(I>2\sigma(I))$ = 0.0449	$\omega R_2(I)$ = 0.1170	$GooF(F^2)$ = 1.023

$[Cu_2(DPEPhos)(CDP(Py)_2)](PF_6)_2$ (6)
MKND134 | CCDC: 2017398

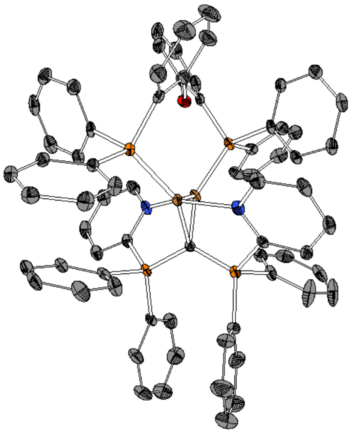

Crystal data

$C_{73}H_{60}Cl_4Cu_2F_{12}N_2OP_6$	Block	Intense Yellow
Triclinic	$P\overline{1}$	Z = 2
a = 13.2598(8) Å	b = 13.7699(8) Å	c = 23.8017(15) Å
α =87.929(2) °	β = 78.411(2) °	γ = 68.542(2) °
V = 3958.9(4) Å^3	μ = 0.864 mm^{-1}	$F(000)$ = 1688
0.520 x 0.216 x 0.199 mm^3	M = 1.66395 kg·mol^{-1}	D_{calc} = 1.396 g·cm^{-3}
N_{ref} = 9851	Θ_{min} = 2.6 °	Θ_{max} = 29.1 °

Data collection

D8 Quest (Bruker AXS Inc.)	T = 100(2) K	λ = 0.71073 Å
Θ_{min} = 2.156 °	Θ_{max} = 25.027 °	
$h = 15 \rightarrow -15$	$k = 16 \rightarrow -16$	$l = 28 \rightarrow -28$

Structure solution / refinement

N_{ref} measured = 81101	N_{ref} independent = 13988	R_{Int} = 0.0466
Semi-empirical from equivalents	T_{max} = 0.7458<br>T_{min} = 0.6532	Full-matrix least-squares on F^2
$C_{25.00\,°}$ = 99.9%	N_{ref} used = 13988	N_{ref} $I>2\sigma(I)$ = 5608
$\Delta\rho_{max}$ = 0.525 eÅ^{-3}	$\Delta\rho_{max}$ = –0.708 eÅ^{-3}	
N_{ref} = 13988	$N_{restraints}$ = 24	$N_{parameters}$ = 908
$R_1(I>2\sigma(I))$ = 0.0510	$\omega R_2(I)$ = 0.1166	$GooF(F^2)$ = 1.076

$[Cu_2(XantPhos)(CDP(Py)_2)](PF_6)_2$ (7)

MKPM123 | CCDC: 2017400

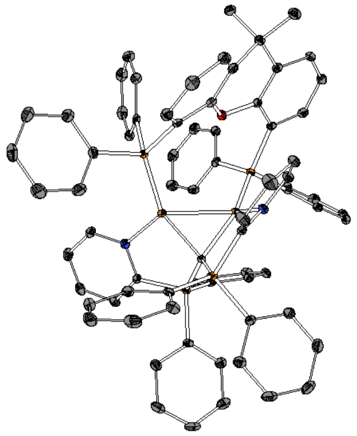

Crystal data

$C_{76}H_{64}Cl_4Cu_2F_{12}N_2OP_6$	Block	Fluorescent Yellow
Triclinic	$P\overline{1}$	Z = 2
a = 12.7458(6) Å	b = 13.1266(6) Å	c = 21.8612(11) Å
α = 87.898(2) °	β = 83.248(2) °	γ = 83.122(2) °
V = 3605.1(3) $Å^3$	μ = 0.951 mm^{-1}	F(000) = 1732
0.324 x 0.267 x 0.172 mm^3	M = 1.70402 kg·mol^{-1}	D_{calc} = 1.570 g·cm^{-3}
N_{ref} = 9459	Θ_{min} = 2.3 °	Θ_{max} = 29.2 °

Data collection

D8 Quest (Bruker AXS Inc.)	T = 100(2) K	λ = 0.71073 Å
Θ_{min} = 2.116 °	Θ_{max} = 25.027 °	
$h = 15 \rightarrow -15$	$k = 15 \rightarrow -15$	$l = 26 \rightarrow -26$

Structure solution / refinement

N_{ref} measured = 80679	N_{ref} independent = 12680	R_{Int} = 0.0270
Semi-empirical from equivalents	T_{max} = 0.7458; T_{min} = 0.6926	Full-matrix least-squares on F^2
$C_{25.00\,°}$ = 99.6%	N_{ref} used = 12680	N_{ref} $I>2\sigma(I)$ = 11570
$\Delta\rho_{max}$ = 0.735 eÅ^{-3}	$\Delta\rho_{max}$ = –0.496 eÅ^{-3}	
N_{ref} = 12680	$N_{restraints}$ = 0	$N_{parameters}$ = 930
$R_1(I>2\sigma(I))$ = 0.0276	$\omega R_2(I)$ = 0.0718	$GooF(F^2)$ = 1.023

$[Cu_2(dppf)(CDP(Py)_2)](PF_6)_2$ (8)
MKPM124 | CCDC: 2017406

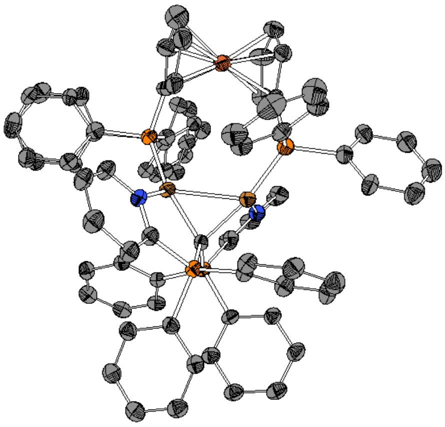

Crystal data

$C_{72}H_{62}Cl_6Cu_2F_{12}FeN_2P_6$	Block	Yellow
Triclinic	$P\bar{1}$	Z = 2
a = 13.1967(3) Å	b = 13.7697(3) Å	c = 22.6159(5) Å
α = 85.242(2) °	β = 81.225(2) °	γ = 67.120(2) °
V = 3740.72(15) $Å^3$	μ = 6.015 mm^{-1}	F(000) = 1784
0.135 x 0.090 x 0.046 mm^3	M = 1.76471 kg·mol^{-1}	D_{calc} = 1.567 g·cm^{-3}
N_{ref} = 27362	$Θ_{min}$ = 3.7 °	$Θ_{max}$ = 75.6 °

Data collection

X-Area Pilatus3_SV 1.31.127.0 (Stoe, 2016)		λ = 1.54178 Å
$Θ_{min}$ = 3.667 °	$Θ_{max}$ = 66.595 °	T = 100(2) K
h = 15 → –14	k = 16 → –14	l = 26→ –23

Structure solution / refinement

N_{ref} measured = 65269	N_{ref} independent = 13098	R_{Int} = 0.0734
Semi-empirical from equivalents	T_{max} = 0.9498; T_{min} = 0.2242	Full-matrix least-squares on F^2
$C_{25.00\,°}$ = 99.0%	N_{ref} used = 13098	N_{ref} I>2σ(I) = 8522
$Δρ_{max}$ = 3.149 e$Å^{-3}$	$Δρ_{max}$ = –1.060 e$Å^{-3}$	
N_{ref} = 13098	$N_{restraints}$ = 100	$N_{parameters}$ = 1098
R_1(I>2σ(I)) = 0.1041	$ωR_2$(I) = 0.3047	$GooF$(F^2) = 1.120

[(CuCarb)$_2$(CDP(Py)$_2$)] (11)

MKML23b | CCDC: 2017393

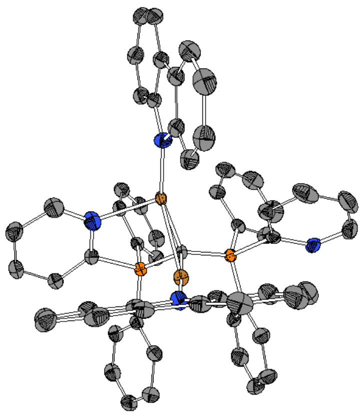

Crystal data

$C_{61}H_{48}Cl_4Cu_2N_4P_2$	Block	Yellow
Monoclinic	$P2_1/n$	Z = 4
a =12.9899(2) Å	b = 26.8839(4) Å	c = 16.5110(2) Å
α = 90 °	β = 111.9520(10) °	γ = 90 °
V = 5347.91(14) Å^3	μ = 3.730 mm^{-1}	F(000) = 2392
0.183 x 0.127 x 0.072 mm^3	M = 1.16787 kg·mol^{-1}	D_{calc} = 1.450 g·cm^{-3}
N_{ref} = 48688	Θ_{min} = 3.3°	Θ_{max} = 76.1 °

Data collection

X-Area Pilatus3_SV 1.31.127.0 (Stoe, 2016)		λ = 1.54178 Å
Θ_{min} = 3.725 °	Θ_{max} = 66.590 °	T = 100(2) K
h = 15 → –15	k = 32 → –19	l = 19→ –19

Structure solution / refinement

N_{ref} measured = 51910	N_{ref} independent = 9382	R_{Int} = 0.0306
Semi-empirical from equivalents	T_{max} = 0.9848;<br>T_{min} = 0.4696	Full-matrix least-squares on F^2
$C_{25.00\,°}$ = 99.3%	N_{ref} used = 9382	N_{ref} I>2σ(I) = 8139
$\Delta\rho_{max}$ = 1.069 eÅ^{-3}	$\Delta\rho_{max}$ = –1.097 eÅ^{-3}	
N_{ref} = 9382	$N_{restraints}$ = 22	$N_{parameters}$ = 680
R_1(I>2σ(I)) = 0.0445	ωR_2(I) = 0.1200	$GooF$(F^2) = 1.031

[(CuS(C_6F_5))$_2$(CDP(Py)$_2$)] (9)
MKMN26 | CCDC: 2017395

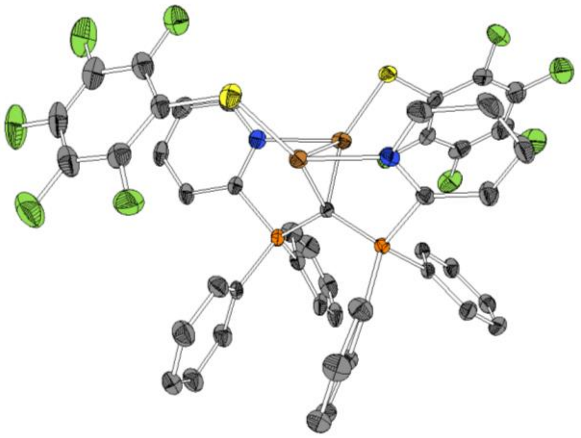

Crystal data

$C_{47}H_{28}Cu_2F_{10}N_2P_2S_2$	Needle	Orange
Triclinic	$P\overline{1}$	Z = 2
a = 10.295(2) Å	b = 13.312(3) Å	c = 16.579(3) Å
α = 71.143(5) °	β = 85.319(6) °	γ = 80.351(6) °
V = 2118.8(8) Å^3	μ = 1.261 mm^{-1}	F(000) = 4032
0.358 x 0.117 x 0.056 mm^3	M = 1.06385 kg·mol^{-1}	D_{calc} = 1.667 g·cm^{-3}
N_{ref} = 9758	Θ_{min} = 2.4°	Θ_{max} = 28.5 °

Data collection

D8 Quest (Bruker AXS Inc.)	T = 100(2) K	λ = 0.71073 Å
Θ_{min} = 2.359 °	Θ_{max} = 29.161 °	
$h = 14 \rightarrow -14$	$k = 18 \rightarrow -18$	$l = 22 \rightarrow -20$

Structure solution / refinement

N_{ref} measured = 69728	N_{ref} independent = 11371	R_{Int} = 0.0698
Semi-empirical from equivalents	T_{max} = 0.7458; T_{min} = 0.6599	Full-matrix least-squares on F^2
$C_{25.00\,°}$ = 99.9%	N_{ref} used = 11371	N_{ref} $I>2\sigma(I)$ = 7885
$\Delta\rho_{max}$ = 2.804 eÅ^{-3}	$\Delta\rho_{max}$ = –1.643 eÅ^{-3}	
N_{ref} = 11371	$N_{restraints}$ = 0	$N_{parameters}$ = 586
$R_1(I>2\sigma(I))$ = 0.0527	$\omega R_2(I)$ = 0.1115	$GooF(F^2)$ = 1.028

$[(CuCl)_2(CDP(CH_2PPh_2)_2]$ (14)

MKMW30 | CCDC: 2017408

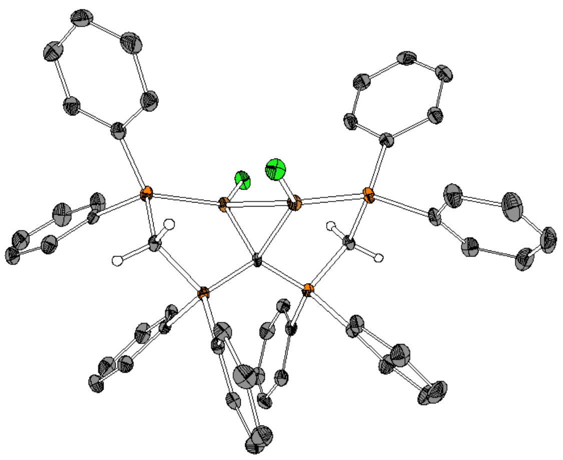

Crystal data

$C_{55}H_{52}Cl_{10}Cu_2P_4$	Block	Clear Colourless
Triclinic	$P\overline{1}$	Z = 2
a = 12.0702(8) Å	b = 12.8612(9) Å	c = 20.0342(13) Å
α = 84.966(2) °	β = 75.555(2) °	γ = 73.976(2) °
V = 2894.1(3) Å^3	μ = 1.344 mm^{-1}	F(000) = 1340
0.560 x 0.390 x 0.240 mm^3	M = 1.31845 kg·mol^{-1}	D_{calc} = 1.513 g·cm^{-3}
N_{ref} = 9305	Θ_{min} = 2.4 °	Θ_{max} = 29.2 °

Data collection

D8 Quest (Bruker AXS Inc.)	T = 100(2) K	λ = 0.71073 Å
Θ_{min} = 2.293 °	Θ_{max} = 25.027 °	
$h = 14 \rightarrow -14$	$k = 15 \rightarrow -15$	$l = 23 \rightarrow -23$

Structure solution / refinement

N_{ref} measured = 64909	N_{ref} independent = 10222	R_{Int} = 0.0377
Semi-empirical from equivalents	T_{max} = 0.7458; T_{min} = 0.6081	Full-matrix least-squares on F^2
$C_{25.00\,°}$ = 99.9%	N_{ref} used = 10222	N_{ref} $I>2\sigma(I)$ = 8783
$\Delta\rho_{max}$ = 0.439 eÅ^{-3}	$\Delta\rho_{max}$ = –0.635 eÅ^{-3}	
N_{ref} = 10222	$N_{restraints}$ = 6	$N_{parameters}$ = 695
$R_1(I>2\sigma(I))$ = 0.0314	$\omega R_2(I)$ = 0.0736	$GooF(F^2)$ = 1.056

$[(CuI)_2(CDP(CH_2PPh_2)_2]$ (15)
MKCR5 | CCDC: 2017410

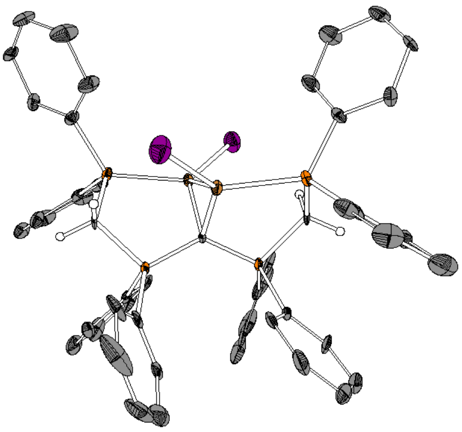

Crystal data

$C_{51}H_{44}Cu_2I_2P_4$	Block	Translucent Yellow
Monoclinic	$C2/c$	Z = 4
a = 15.1057(8) Å	b = 17.1264(9) Å	c = 20.0035(11) Å
α = 90 °	β = 93.099(2) °	γ = 90 °
V = 5167.5(5) $Å^3$	μ = 2.173 mm^{-1}	$F(000)$ = 2296
0.440 x 0.286 x 0.231 mm^3	M = 1.16162 $kg{\cdot}mol^{-1}$	D_{calc} = 1.4923$g{\cdot}cm^{-3}$
N_{ref} = 9712	Θ_{min} = 2.4 °	Θ_{max} = 28.5 °

Data collection

D8 Quest (Bruker AXS Inc.)	T = 100(2) K	λ = 0.71073 Å
Θ_{min} = 2.104 °	Θ_{max} = 29.272 °	
$h = 20 \rightarrow -19$	$k = 23 \rightarrow -23$	$l = 26 \rightarrow -26$

Structure solution / refinement

N_{ref} measured = 82870	N_{ref} independent = 6967	R_{Int} = 0.0740
Semi-empirical from equivalents	T_{max} = 0.7458; T_{min} = 0.6741	Full-matrix least-squares on F^2
$C_{25.00\,°}$ = 99.9%	N_{ref} used = 6967	N_{ref} $I>2\sigma(I)$ = 5325
$\Delta\rho_{max}$ = 2.286 $eÅ^{-3}$	$\Delta\rho_{max}$ = –2.211 $eÅ^{-3}$	
N_{ref} = 6967	$N_{restraints}$ = 12	$N_{parameters}$ = 268
$R_1(I>2\sigma(I))$ = 0.0603	$\omega R_2(I)$ = 0.1889	$GooF(F^2)$ = 1.043

[$(CuSPh)_2(CDP(CH_2PPh_2)_2]$ (16)
MKP199 | CCDC: 2017409

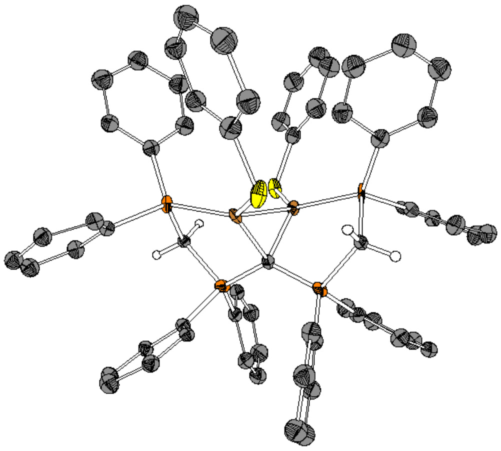

Crystal data

$C_{138}H_{132}Cu_4O_3P_8S_4$	Needle	Fluorescent Yellow
Triclinic	$P\overline{1}$	Z = 2
a = 16.3134(17) Å	b = 20.256(2) Å	c = 20.393(2) Å
α = 63.399(3) °	β = 84.454(3) °	γ = 86.908(3) °
V = 5996.6(11) $Å^3$	μ = 0.930 mm^{-1}	$F(000)$ = 2568
0.693 x 0.179 x 0.098 mm^3	M = 2.46864 kg·mol^{-1}	D_{calc} = 1.367 g·cm^{-3}
N_{ref} = 7286	Θ_{min} = 2.2 °	Θ_{max} = 23.9 °

Data collection

D8 Quest (Bruker AXS Inc.)	T = 100(2) K	λ = 0.71073 Å
Θ_{min} = 2.241 °	Θ_{max} = 25.027 °	
$h = 19 \rightarrow -19$	$k = 21 \rightarrow -24$	$l = 24 \rightarrow 0$

Structure solution / refinement

N_{ref} measured = 21786	N_{ref} independent = 21786	R_{Int} = 0.00
Semi-empirical from equivalents	T_{max} = 0.205084;<br>T_{min} = 0.161605	Full-matrix least-squares on F^2
$C_{25.00\,°}$ = 99.7%	N_{ref} used = 21786	N_{ref} $I>2\sigma(I)$ = 12003
$\Delta\rho_{max}$ = 0.994 e$Å^{-3}$	$\Delta\rho_{max}$ = –0.940 e$Å^{-3}$	
N_{ref} = 21786	$N_{restraints}$ = 2036	$N_{parameters}$ = 1415
$R_1(I>2\sigma(I))$ = 0.1125	$\omega R_2(I)$ = 0.1874	$GooF(F^2)$ = 1.050

[CuCl(CDPH(CH2PPh2)2]PF6 (17)
MKP42 | CCDC: 2017407

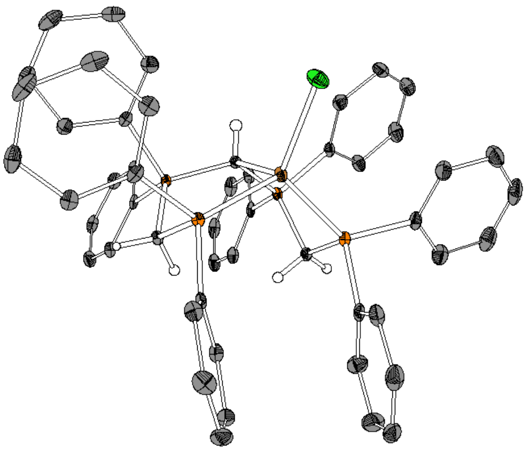

Crystal data

$C_{52}H_{47}Cl_3CuF_6P_5$	Block	Cleat Colourless
Monoclinic	$C2/c$	Z = 2
a = 23.7049(12) Å	b = 11.6976(6) Å	c = 38.147(2) Å
α = 90 °	β = 105.731(2) °	γ = 90 °
V = 10181.5(10) $Å^3$	μ = 0.801 mm^{-1}	$F(000)$ = 4544
0.440 x 0.221 x 0.186 mm^3	M = 1.449 $kg{\cdot}mol^{-1}$	D_{calc} = 1.449 $g{\cdot}cm^{-3}$
N_{ref} = 9113	Θ_{min} = 2.2 °	Θ_{max} = 27.1 °

Data collection

D8 Quest (Bruker AXS Inc.)	T = 100(2) K	λ = 0.71073 Å
Θ_{min} = 2.219 °	Θ_{max} = 25.024 °	
$h = 28 \rightarrow -28$	$k = 13 \rightarrow -13$	$l = 45 \rightarrow -45$

Structure solution / refinement

N_{ref} measured = 114090	N_{ref} independent = 8988	R_{Int} = 0.0449
Semi-empirical from equivalents	T_{max} = 0.7455;<br>T_{min} = 0.7042	Full-matrix least-squares on F^2
$C_{25.00\,°}$ = 99.9%	N_{ref} used = 8988	N_{ref} $I>2\sigma(I)$ = 7804
$\Delta\rho_{max}$ = 0.748 $eÅ^{-3}$	$\Delta\rho_{max}$ = –0.941 $eÅ^{-3}$	
N_{ref} = 8988	$N_{restraints}$ = 0	$N_{parameters}$ = 625
$R_1(I>2\sigma(I))$ = 0.0332	$\omega R_2(I)$ = 0.0755	$GooF(F^2)$ = 1.050

[$CuPPh_3(CH(PPh_2CHPPh_2)_2]$ (18)

MKPCR28 | CCDC: 2017411

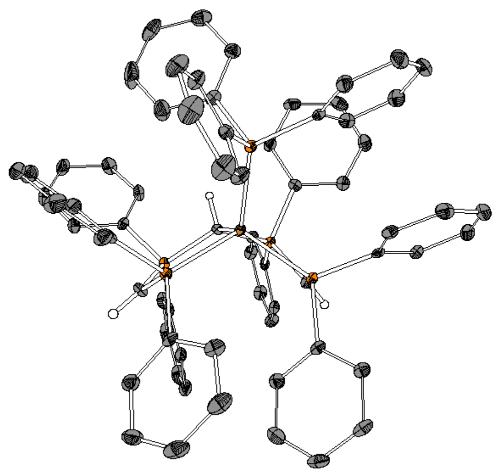

Crystal data

$C_{73}H_{66}CuOP_5$	Block	Yellow
Monoclinic	$P2_1/c$	Z = 4
a = 15.5803(8) Å	b = 15.8868(8) Å	c = 24.0979(11) Å
α = 90 °	β = 94.733(2) °	γ = 90 °
V = 5944.4(5) Å^3	μ = 0.548 mm^{-1}	F(000) = 2464
0.440 x 0.221 x 0.186 mm^3	M = 1.17764 kg·mol^{-1}	D_{calc} = 1.492 g·cm^{-3}
N_{ref} = 9982	Θ_{min} = 2.4 °	Θ_{max} = 28.7 °

Data collection

D8 Quest (Bruker AXS Inc.)	T = 100(2) K	λ = 0.71073 Å
Θ_{min} = 2.065 °	Θ_{max} = 25.025 °	
h = 18 → –18	k = 18 → –18	l = 28→ –28

Structure solution / refinement

N_{ref} measured = 104671	N_{ref} independent = 10497	R_{Int} = 0.0679
Semi-empirical from equivalents	T_{max} = 0.7458; T_{min} = 0.6758	Full-matrix least-squares on F^2
$C_{25.00\,°}$ = 99.9%	N_{ref} used = 10497	N_{ref} I>2σ(I) = 8931
$\Delta\rho_{max}$ = 0.838 eÅ^{-3}	$\Delta\rho_{max}$ = –0.424 eÅ^{-3}	
N_{ref} = 10497	$N_{restraints}$ = 0	$N_{parameters}$ = 721
R_1(I>2σ(I)) = 0.0471	ωR_2(I) = 0.1098	$GooF$(F^2) = 1.164

Chemical calculations

Density functional theory (DFT) using the PBE[8,9] functional was performed to complex **8** and **10**. The def2-TZVPP[10–12] basis set was used, employing the resolution-of-identity approximation.[13–16] Further, D3-dispersion correction[17] was considered applying the BECKE-JOHNSON damping.[18–21] To verify that ground state and excited states are minima on the potential energy surface, analytical harmonic vibrational frequency calculations were conducted. Structural optimizations, TD-DFT and SOC-TD-DFT calculations were performed using ORCA 3.0.3.[22]

Deprotonation of 19

Table 1 Atomic partial charges *q*(C) of **19** calculated by the NBO method at the PBE-D3(BJ)/def2-TZVPP level of theory.

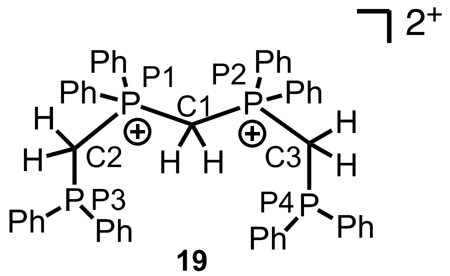

19

	q(C)
C1	-1.10401
C2	-1.09837
C3	-1.09833
P1	1.67805
P2	1.67807
P3	0.83356
P4	0.83358
Sum =	1.72255

Table 2 Atomic partial charges *q*(C) of **19a** calculated by the NBO method at the PBE-D3(BJ)/def2-TZVPP level of theory.

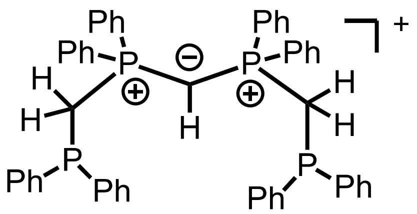

12a

	q(C)
C1	-1.39295
C2	-1.08756
C3	-1.08921
P1	1.67469
P2	1.66442

P3	0.82549
P4	0.82719
Sum =	1.42207

Table 3 Atomic partial charges *q*(C) of **12b** calculated by the NBO method at the PBE-D3(BJ)/def2-TZVPP level of theory.

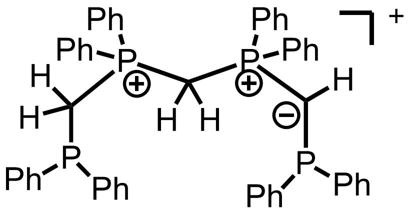

	q(C)
C1	-1.10903
C2	-1.11819
C3	-1.34607
P1	1.69060
P2	1.66433
P3	0.82873
P4	0.82147
Sum =	1.43184

Table 4 Atomic partial charges *q*(C) of **13a** calculated by the NBO method at the PBE-D3(BJ)/def2-TZVPP level of theory.

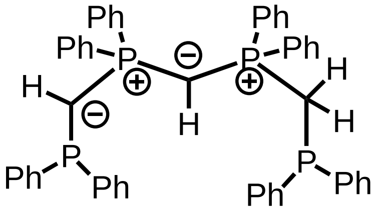

	q(C)
C1	-1.38484
C2	-1.35280
C3	-1.10428
P1	1.66372
P2	1.68057
P3	0.81241
P4	0.80365
Sum =	1.11843

Table 5 Atomic partial charges q(C) of **13b** calculated by the NBO method at the PBE-D3(BJ)/def2-TZVPP level of theory.

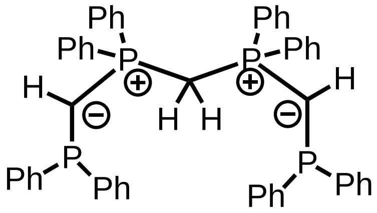

13b

	q(C)
C1	-1.07747
C2	-1.34641
C3	-1.34810
P1	1.66134
P2	1.66292
P3	0.82119
P4	0.82274
Sum =	1.19621

Table 6 Atomic partial charges q(C) of **13c** calculated by the NBO method at the PBE-D3(BJ)/def2-TZVPP level of theory.

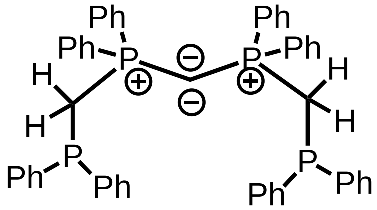

13c

	q(C)
C1	-1.45006
C2	-1.09378
C3	-1.09380
P1	1.61564
P2	1.61565
P3	0.81602
P4	0.81602
Sum =	1. 22569

Table 7 Atomic partial charges *q*(C) of **13d** calculated by the NBO method at the PBE-D3(BJ)/def2-TZVPP level of theory.

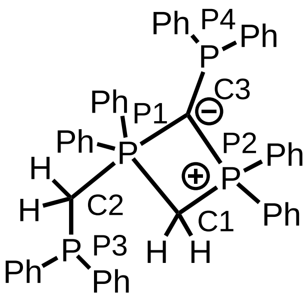

13d

	q(C)
C1	-1.04315
C2	-1.06590
C3	-1.38125
P1	1.58938
P2	1.58578
P3	0.82062
P4	0.79698
Sum =	1.30246

Table 8 Atomic partial charges *q*(C) of **20a** calculated by the NBO method at the PBE-D3(BJ)/def2-TZVPP level of theory.

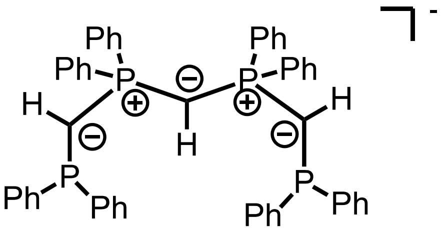

20a

	q(C)
C1	-1.38576
C2	-1.37257
C3	-1.37253
P1	1.67775
P2	1.67769
P3	0.83322
P4	0.83321
Sum =	0.89101

Table 8 Atomic partial charges q(C) of **20b** calculated by the NBO method at the PBE-D3(BJ)/def2-TZVPP level of theory.

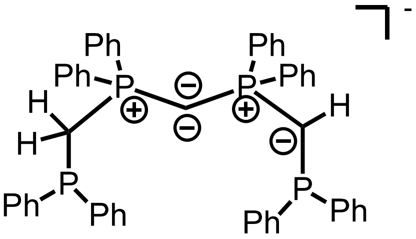

20b

	q(C)
C1	-1.49850
C2	-1.11141
C3	-1.36223
P1	1.59666
P2	1.64607
P3	0.82008
P4	0.82936
Sum =	0.92003

Table 8 Atomic partial charges q(C) of **20c** calculated by the NBO method at the PBE-D3(BJ)/def2-TZVPP level of theory.

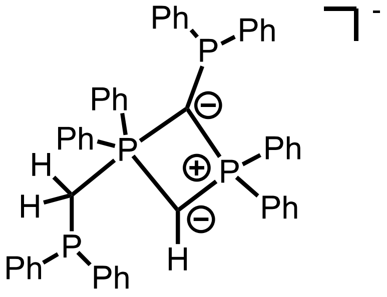

20c

	q(C)
C1	-1.31933
C2	-1.06983
C3	-1.41583
P1	1.58006
P2	1.60939
P3	0.83700
P4	0.84920
Sum =	1.07066

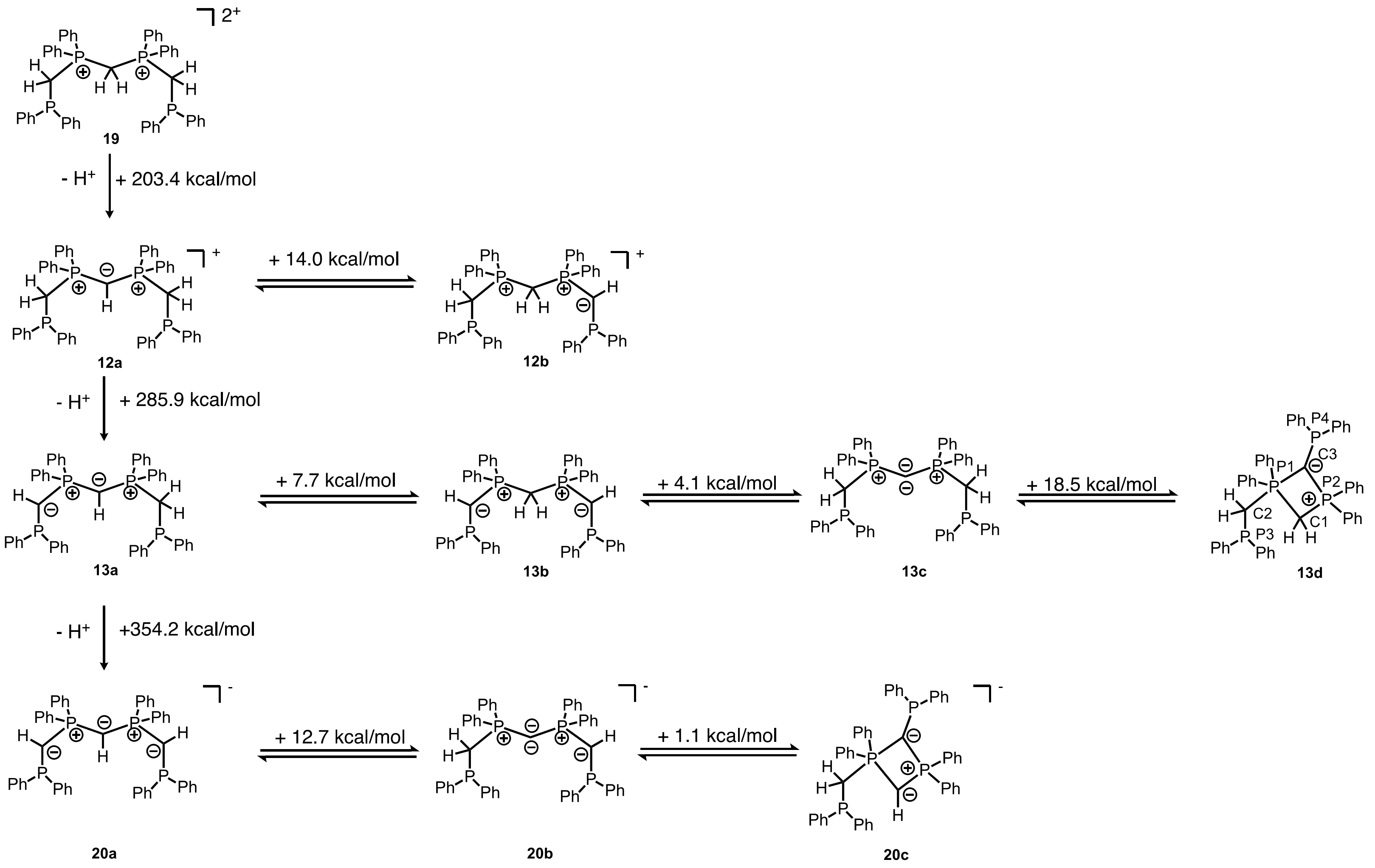

Scheme S-1. Calculation of the stepwise deprotonation of $[CH_2(PPh_2CHPPh_2)_2]Cl_2$ (**19**). Stable tautomers are shown with their corresponding energy difference in kcal/mol. The positive value of the engery corresponds to the energy that has to be applied in order to convert one molecule into the other. The most stable tautomer is shown on the left side.

Kohn-Sham orbitals

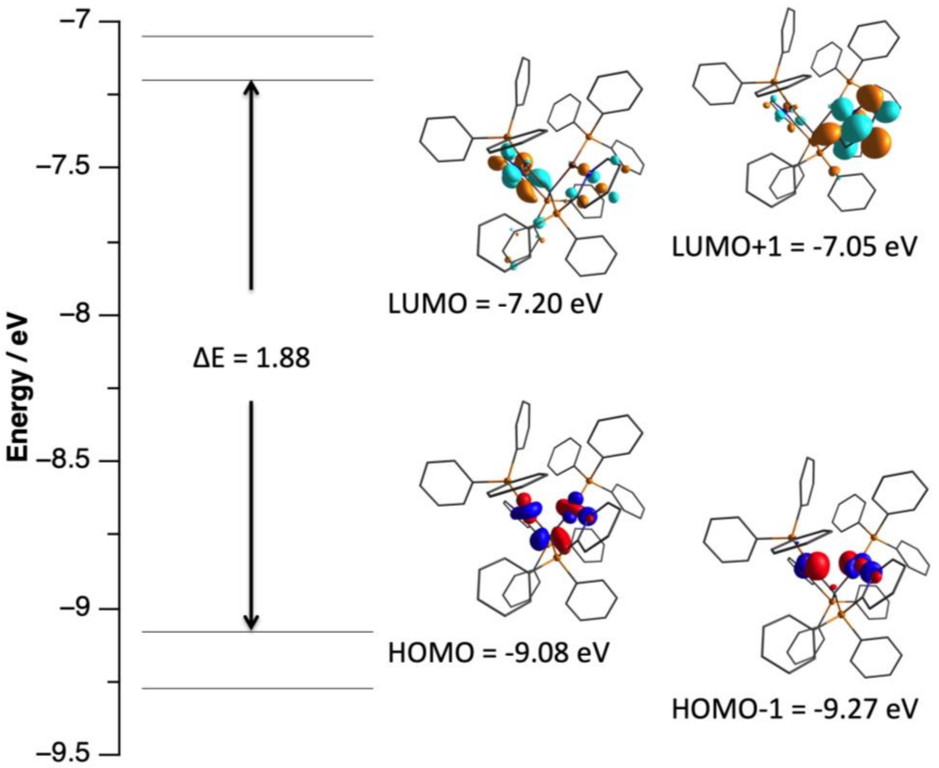

Figure S-43. Kohn-Sham orbitals of HOMO-1, HOMO, LUMO and LUMO+1 for $[(CuCl)_2(CDP(CH_2PPh_2)_2]$ (**14**)calculated for the optimized gas phase S_0 state geometry (isovalue = 0.05). Calculations were performed at the PBE-D3(BJ)/def2-TZVPP level of theory.

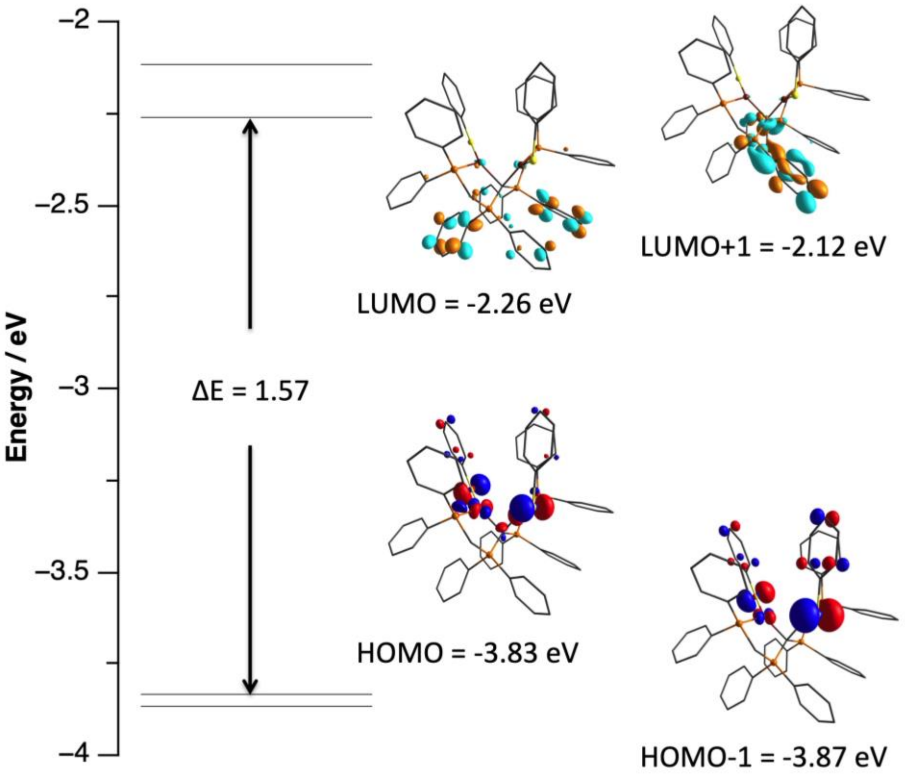

Figure S-44. Kohn-Sham orbitals of HOMO-1, HOMO, LUMO and LUMO+1 for $[(CuSPh)_2(CDP(CH_2PPh_2)_2]$ (**16**) calculated for the optimized gas phase S_0 state geometry (isovalue = 0.05). Calculations were performed at the PBE-D3(BJ)/def2-TZVPP level of theory.

Atomic coordinates

For Atomic coordinates see the XYZ file.

Literature

(1) Armarego, W. L. F.; Perin, D. D. *Purification of Laboratory Chemicals*, 4th ed.; Butterworth Heinemann: Oxford, Aucklnad, Boston, Johannesburg, Melbourne, New Delhi, 1996.

(2) Klein, M.; Xie, X.; Burghaus, O.; Sundermeyer, J. Synthesis and Characterization of a N,C,N-Carbodiphosphorane Pincer Ligand and Its Complexes. *Organometallics* **2019**.

(3) Stallinger, S.; Reitsamer, C.; Schuh, W.; Kopacka, H.; Wurst, K.; Peringer, P. Novel Route to Carbodiphosphoranes Producing a New P,C,P Pincer Carbene Ligand. *Chem. Commun.* **2007**, No. 5, 510–512.

(4) Reitsamer, C.; Stallinger, S.; Schuh, W.; Kopacka, H.; Wurst, K.; Obendorf, D.; Peringer, P. Novel Access to Carbodiphosphoranes in the Coordination Sphere of Group 10 Metals: Template Synthesis and Protonation of PCP Pincer Carbodiphosphorane Complexes of C(Dppm)2. *Dalt. Trans.* **2012**, *41* (12), 3503–3514.

(5) Fulmer, G. R.; Miller, A. J. M.; Sherden, N. H.; Gottlieb, H. E.; Nudelman, A.; Stoltz, B. M.; Bercaw, J. E.; Goldberg, K. I. NMR Chemical Shifts of Trace Impurities: Common Laboratory Solvents, Organics, and Gases in Deuterated Solvents Relevant to the Organometallic Chemist. *Organometallics* **2010**, *29* (9), 2176–2179.

(6) Williams, D. B. G.; Lawton, M. Drying of Organic Solvents: Quantitative Evaluation of the Efficiency of Several Desiccants. *J. Org. Chem.* **2010**, *75* (24), 8351–8354.

(7) Reitsamer, C.; Schuh, W.; Kopacka, H.; Wurst, K.; Peringer, P. Synthesis and Structure of the First Heterodinuclear PCP−Pincer−CDP Complex with a Pd−Au D8−d10 Pseudo-Closed-Shell Interaction. *Organometallics* **2009**, *28* (22), 6617–6620.

(8) Perdew, J.; Burke, K.; Ernzerhof, M. Generalized Gradient Approximation Made Simple. *Phys. Rev. Lett.* **1996**, *77* (18), 3865–3868.

(9) Perdew, J. P.; Burke, K.; Ernzerhof, M. Errata: Generalized Gradient Approximation Made Simple. *Phys. Rev. Lett.* **1997**, *78* (7), 1396.

(10) Schäfer, A.; Huber, C.; Ahlrichs, R. Fully Optimized Contracted Gaussian Basis Sets of Triple Zeta Valence Quality for Atoms Li to Kr. *J. Chem. Phys.* **1994**, *100* (8), 5829–5835.

(11) Weigend, F.; Ahlrichs, R. Balanced Basis Sets of Split Valence{,} Triple Zeta Valence and Quadruple Zeta Valence Quality for H to Rn: Design and Assessment of Accuracy. *Phys. Chem. Chem. Phys.* **2005**, *7* (18), 3297–3305.

(12) Weigend, F. Accurate Coulomb-Fitting Basis Sets for H to Rn. *Phys. Chem. Chem. Phys.* **2006**, *8* (9), 1057–1065.

(13) Weigend, F. A Fully Direct RI-HF Algorithm: Implementation, Optimised Auxiliary Basis Sets, Demonstration of Accuracy and Efficiency. *Phys. Chem. Chem. Phys.* **2002**, *4* (18), 4285–4291.

(14) Eichkorn, K.; Treutler, O.; Öhm, H.; Häser, M.; Ahlrichs, R. Auxiliary Basis Sets to Approximate Coulomb Potentials. *Chem. Phys. Lett.* **1995**, *240* (4), 283–290.

(15) Eichkorn, K.; Weigend, F.; Treutler, O.; Ahlrichs, R. Auxiliary Basis Sets for Main Row Atoms and Transition Metals and Their Use to Approximate Coulomb Potentials. *Theor. Chem. Acc.* **1997**, *97* (1–4), 119–124.

(16) Von Arnim, M.; Ahlrichs, R. Performance of Parallel TURBOMOLE for Density Functional Calculations. *J. Comput. Chem.* **1998**, *19* (15), 1746–1757.

(17) Grimme, S.; Antony, J.; Ehrlich, S.; Krieg, H. A Consistent and Accurate Ab Initio Parametrization of Density Functional Dispersion Correction (DFT-D) for the 94 Elements H-Pu. *J. Chem. Phys.* **2010**, *132* (15), 154104–154119.

(18) Becke, A. D.; Johnson, E. R. A Density-Functional Model of the Dispersion Interaction. *J. Chem. Phys.* **2005**, *123* (15), 154101–154109.

(19) Johnson, E. R.; Becke, A. D. A Post-Hartree-Fock Model of Intermolecular Interactions. *J. Chem. Phys.* **2005**, *123* (2), 24101–24107.

(20) Johnson, E. R.; Becke, A. D. A Post-Hartree-Fock Model of Intermolecular Interactions: Inclusion of Higher-Order Corrections. *J. Chem. Phys.* **2006**, *124* (17), 174104–174109.

(21) Grimme, S.; Ehrlich, S.; Goerigk, L. Effect of the Damping Function in Dispersion Corrected Density Functional Theory. *J. Comput. Chem.* **2011**, *32* (7), 1456–1465.

(22) Neese, F. The ORCA Program System. *Wiley Interdiscip. Rev. Comput. Mol. Sci.* **2011**, *2* (1), 73–78.

Cartesian coordinates of calculated structures (XYZ).

Atomic coordinates of $[(CuPPh_3)_2(CDP(Py)_2)](PF_6)_2$ (**4**) optimized (PBE-D3(BJ)/def2-TZVPP) in the ground state S_0 in the gas phase.

C	9.682569000	-3.076939000	6.222884000
C	4.133480000	-2.809885000	10.067991000
H	3.267635000	-2.280305000	9.669426000
C	8.366448000	0.376654000	1.027132000
H	7.649098000	-0.388528000	0.730428000
C	5.360387000	0.371049000	9.244084000
C	8.749802000	2.373392000	7.680944000
C	13.845768000	-2.740264000	9.438367000
H	14.603646000	-3.036305000	10.163644000
C	11.334731000	-1.082793000	4.838133000
C	5.826349000	1.431306000	3.552941000
C	11.567150000	-2.112866000	8.930361000
H	10.546472000	-1.918552000	9.258588000
C	7.283953000	3.294171000	6.143257000
H	6.744729000	3.167103000	5.204302000
C	9.857650000	0.788395000	9.839297000
C	8.393855000	0.841720000	2.348671000
C	9.586724000	-5.392130000	5.595499000
H	10.026311000	-6.270936000	5.124066000
C	12.533946000	-2.497450000	9.857074000
H	12.263399000	-2.607159000	10.907333000
C	3.956009000	-3.801732000	11.031831000
H	2.952487000	-4.039764000	11.384548000
C	4.927254000	2.754730000	9.143284000

H	4.738301000	3.652824000	8.555009000
C	6.540368000	-1.302512000	3.014159000
C	11.909268000	-1.959753000	7.576080000
C	10.279284000	-4.180363000	5.613012000
H	11.269313000	-4.093515000	5.166356000
C	8.950884000	-0.128353000	10.386442000
H	8.326938000	-0.727266000	9.714785000
C	12.955601000	0.195755000	3.567315000
H	13.795954000	0.889696000	3.543672000
C	9.260125000	0.892554000	0.086042000
H	9.231480000	0.528640000	-0.941240000
C	9.334739000	1.817188000	2.717296000
H	9.373954000	2.164093000	3.752877000
C	3.694832000	2.126822000	4.481534000
H	2.863923000	1.948040000	5.164563000
C	12.413685000	-0.182747000	4.794224000
H	12.835980000	0.218131000	5.714509000
C	11.551287000	1.692474000	7.642865000
C	12.712758000	1.191621000	8.254991000
H	12.641361000	0.437698000	9.037957000
C	8.332084000	-5.460986000	6.201231000
H	7.764276000	-6.390235000	6.222171000
C	10.180279000	1.876603000	0.454388000
H	10.870161000	2.282199000	-0.285827000
C	10.217316000	2.338116000	1.772895000
H	10.940573000	3.099277000	2.066223000
C	9.629495000	0.502427000	12.616192000
H	9.547514000	0.387237000	13.697004000
C	3.698593000	3.253913000	3.652476000

H	2.870681000	3.962009000	3.688669000
C	14.070971000	2.625030000	6.853572000
H	15.051670000	2.993116000	6.552255000
C	5.822019000	2.555552000	2.715901000
H	6.646143000	2.725124000	2.022892000
C	5.421826000	-2.500684000	9.605606000
C	5.524393000	-1.321464000	2.046487000
H	5.096607000	-0.385021000	1.686684000
C	4.352086000	-1.366570000	7.160703000
C	4.925853000	2.819096000	10.540214000
H	4.740663000	3.767705000	11.044272000
C	7.808374000	-4.314455000	6.795180000
H	6.835797000	-4.321936000	7.289076000
C	5.355066000	0.439334000	10.643486000
H	5.499667000	-0.465778000	11.233287000
C	14.182824000	-2.615072000	8.088657000
H	15.201929000	-2.816673000	7.758826000
C	4.759670000	3.463516000	2.769865000
H	4.760800000	4.333194000	2.112464000
C	12.918029000	3.127765000	6.244766000
H	12.996637000	3.888497000	5.468186000
C	5.146128000	1.661332000	11.287058000
H	5.138176000	1.702881000	12.376346000
C	13.218666000	-2.232736000	7.154366000
H	13.491597000	-2.138961000	6.104244000
C	3.103026000	-0.747801000	7.324383000
H	2.921427000	-0.099067000	8.181496000
C	4.753478000	1.221880000	4.437162000
H	4.742974000	0.343902000	5.084965000

C	5.053452000	-2.538114000	1.550664000
H	4.267032000	-2.546374000	0.795807000
C	5.059573000	-4.491349000	11.543841000
H	4.915349000	-5.266390000	12.296548000
C	8.650723000	3.486341000	8.513324000
H	9.195818000	3.520910000	9.455510000
C	12.430513000	-0.315103000	2.378012000
H	12.859733000	-0.021127000	1.420575000
C	6.344255000	-4.192883000	11.085535000
H	7.205429000	-4.734381000	11.477847000
C	8.837761000	-0.274834000	11.766596000
H	8.130390000	-0.995717000	12.177498000
C	5.586132000	-3.742243000	2.020034000
H	5.216145000	-4.690370000	1.629574000
C	10.643507000	1.576630000	10.696800000
H	11.353504000	2.296987000	10.290879000
C	13.966050000	1.657422000	7.856389000
H	14.862695000	1.265910000	8.336615000
C	7.842034000	4.550408000	8.112316000
H	7.749443000	5.437688000	8.738242000
C	2.321993000	-1.772984000	5.272535000
H	1.531765000	-1.925806000	4.537537000
C	6.523583000	-3.203891000	10.116129000
H	7.526190000	-2.978507000	9.744074000
C	7.057743000	-2.513330000	3.492660000
H	7.818823000	-2.490919000	4.272642000
C	10.799697000	-1.578658000	3.638337000
H	9.949055000	-2.258365000	3.649096000
C	4.573337000	-2.186314000	6.043606000

H	5.550360000	-2.648061000	5.896317000
C	6.586082000	-3.728935000	2.996246000
H	6.994233000	-4.666797000	3.374221000
C	3.562243000	-2.393236000	5.105994000
H	3.751883000	-3.024360000	4.238214000
C	7.156785000	4.455844000	6.902538000
H	6.519559000	5.264769000	6.547495000
N	8.070366000	2.268987000	6.521699000
N	8.467208000	-3.140929000	6.801642000
P	7.234889000	0.264950000	3.637786000
P	5.713251000	-1.190301000	8.364372000
P	9.918368000	0.993914000	8.031623000
P	10.576805000	-1.477568000	6.436021000
Cu	8.232395000	0.383608000	5.602609000
Cu	7.729865000	-1.315633000	7.488155000
C	9.432460000	-0.342734000	7.055836000
C	5.157997000	1.542863000	8.495811000
H	5.157779000	1.502302000	7.405207000
C	11.662442000	2.662208000	6.632827000
H	10.769472000	3.055674000	6.146757000
C	10.526709000	1.429313000	12.079901000
H	11.143804000	2.038380000	12.740303000
C	2.094984000	-0.951934000	6.380611000
H	1.125893000	-0.470252000	6.513571000
C	11.351102000	-1.199123000	2.415813000
H	10.928961000	-1.588873000	1.490342000

Atomic coordinates of $[(CuSPh)_2(CDP(CH_2PPh_2)_2]$ (**16**) optimized (PBE-D3(BJ)/def2-TZVPP) in the ground state S_0 in the gas phase.

C	7.280043000	9.351566000	5.006288000
C	6.855258000	8.810595000	2.207455000
H	7.802462000	8.254804000	2.118951000
H	6.597817000	9.268757000	1.243875000
C	5.613390000	11.168240000	3.271097000
C	4.502640000	10.952421000	4.095660000
H	4.563237000	10.215508000	4.898614000
C	3.318535000	11.655414000	3.877594000
H	2.459168000	11.469807000	4.521542000
C	3.235515000	12.578482000	2.834487000
H	2.307768000	13.124928000	2.661188000
C	4.338868000	12.795943000	2.004401000
H	4.274975000	13.511942000	1.184267000
C	5.523159000	12.091039000	2.216968000
H	6.378514000	12.258167000	1.561179000
C	8.501483000	11.136991000	2.963496000
C	9.620745000	10.535987000	2.366851000
H	9.613077000	9.475823000	2.108796000
C	10.771750000	11.288379000	2.129201000
H	11.637564000	10.810039000	1.670804000
C	10.816240000	12.638589000	2.484665000
H	11.718832000	13.222231000	2.300535000
C	9.705075000	13.240052000	3.080750000
H	9.738462000	14.291351000	3.367925000
C	8.552591000	12.493379000	3.321282000
H	7.689109000	12.963015000	3.793880000
C	3.975759000	8.211958000	2.317235000
C	2.893543000	7.972556000	3.178530000

H	3.073522000	7.482419000	4.139385000
C	1.609674000	8.388768000	2.822936000
H	0.774643000	8.196072000	3.497568000
C	1.399591000	9.061068000	1.617339000
H	0.398349000	9.397793000	1.345469000
C	2.474815000	9.304996000	0.758152000
H	2.314361000	9.830125000	-0.184233000
C	3.756026000	8.873530000	1.101432000
H	4.584261000	9.055303000	0.415525000
C	5.875746000	6.076153000	1.914144000
C	7.148292000	5.481698000	1.935964000
H	7.992431000	5.999396000	2.398447000
C	7.343228000	4.219921000	1.377375000
H	8.336479000	3.770508000	1.417283000
C	6.271900000	3.534183000	0.797872000
H	6.422420000	2.541776000	0.371398000
C	5.005002000	4.121910000	0.774799000
H	4.163466000	3.591971000	0.326648000
C	4.801984000	5.386001000	1.330642000
H	3.806373000	5.829464000	1.320206000
P	7.091039000	10.114877000	3.480950000
P	5.608951000	7.605792000	2.871780000
Cu	5.863253000	7.927078000	5.104538000
C	7.701980000	8.760396000	7.794771000
H	6.754044000	8.204275000	7.872194000
H	7.959708000	9.200236000	8.766780000
C	8.945452000	11.137464000	6.774898000
C	10.056470000	10.937353000	5.946743000
H	9.997162000	10.214626000	5.130980000

C	11.239819000	11.637661000	6.177340000
H	12.099450000	11.464319000	5.530338000
C	11.321772000	12.542327000	7.236522000
H	12.248875000	13.086726000	7.419444000
C	10.218186000	12.743806000	8.070307000
H	10.281253000	13.445290000	8.902957000
C	9.034659000	12.041511000	7.845253000
H	8.179102000	12.196119000	8.503832000
C	6.057089000	11.100025000	7.079866000
C	4.937009000	10.487850000	7.663541000
H	4.944364000	9.422996000	7.901545000
C	3.785583000	11.235588000	7.913529000
H	2.919177000	10.748736000	8.361740000
C	3.741400000	12.592186000	7.583187000
H	2.838420000	13.172148000	7.776799000
C	4.853326000	13.204775000	7.000060000
H	4.820205000	14.261218000	6.732404000
C	6.006287000	12.462825000	6.747376000
H	6.870440000	12.941350000	6.285051000
C	10.581155000	8.163988000	7.669796000
C	11.661769000	7.937220000	6.803082000
H	11.479964000	7.461186000	5.835531000
C	12.946212000	8.348700000	7.162117000
H	13.779947000	8.165971000	6.483131000
C	13.158397000	9.003832000	8.376751000
H	14.160007000	9.337191000	8.651411000
C	12.084784000	9.234992000	9.241475000
H	12.246909000	9.746774000	10.190888000
C	10.803077000	8.807962000	8.894612000

H	9.976127000	8.979601000	9.584669000
C	8.682399000	6.021853000	8.043772000
C	7.409120000	5.429140000	8.020141000
H	6.562451000	5.954914000	7.571549000
C	7.216554000	4.158298000	8.558813000
H	6.222741000	3.710333000	8.517136000
C	8.290569000	3.462147000	9.120457000
H	8.141605000	2.462971000	9.531390000
C	9.558151000	4.048434000	9.145867000
H	10.401814000	3.510616000	9.580418000
C	9.758988000	5.321212000	8.609531000
H	10.755087000	5.763542000	8.621304000
P	7.468273000	10.087834000	6.544821000
P	8.946681000	7.566320000	7.109422000
Cu	8.694489000	7.923249000	4.884525000
S	4.490711000	7.082051000	6.614658000
S	10.067461000	7.117903000	3.352023000
C	4.360258000	5.376162000	6.158350000
C	5.281660000	4.755887000	5.292071000
C	3.326052000	4.586970000	6.697572000
C	5.163631000	3.407615000	4.959012000
C	3.217560000	3.235230000	6.371534000
C	4.131422000	2.635647000	5.498190000
H	6.099716000	5.345329000	4.875681000
H	2.606176000	5.049535000	7.374296000
H	5.890191000	2.964936000	4.275407000
H	2.406128000	2.644832000	6.801250000
H	4.039659000	1.579544000	5.243024000
C	10.215864000	5.402995000	3.766966000

C	9.312007000	4.756246000	4.632381000
C	11.246920000	4.633832000	3.193680000
C	9.443590000	3.401070000	4.930917000
C	11.369013000	3.275519000	3.485581000
C	10.472160000	2.649044000	4.357749000
H	8.497072000	5.330286000	5.075349000
H	11.953210000	5.117286000	2.517231000
H	8.730098000	2.937267000	5.614316000
H	12.177477000	2.700955000	3.029618000
H	10.574324000	1.587685000	4.585695000

Atomic coordinates of $[CH_2(dppm)_2]Cl_2$ (**19**) optimized (PBE-D3(BJ)/def2-TZVPP) in the ground state S_0 in the gas phase.

P	13.673184000	20.273340000	8.094706000
P	11.496699000	21.725838000	9.660522000
C	15.237835000	20.512293000	7.145058000
H	15.113870000	21.390494000	6.494200000
H	15.361946000	19.633888000	6.494500000
C	13.250948000	21.799004000	8.943265000
H	13.385012000	22.630857000	8.238270000
H	13.951465000	21.954933000	9.772580000
C	12.397683000	19.853003000	6.911366000
C	11.427885000	18.897275000	7.258532000
H	11.506088000	18.344685000	8.194037000
C	10.352278000	18.671928000	6.400906000
H	9.599734000	17.929717000	6.665226000
C	10.236674000	19.398464000	5.214154000
H	9.391225000	19.221058000	4.549746000

C	11.197025000	20.355707000	4.874034000
H	11.099160000	20.926976000	3.951701000
C	12.278624000	20.588058000	5.718944000
H	13.009702000	21.351663000	5.450568000
C	13.950801000	18.928543000	9.247426000
C	14.217269000	17.648373000	8.728160000
H	14.120369000	17.453321000	7.658608000
C	14.604349000	16.621286000	9.586819000
H	14.810585000	15.629222000	9.186172000
C	14.731874000	16.865341000	10.957456000
H	15.058450000	16.067527000	11.623832000
C	14.438137000	18.128691000	11.477301000
H	14.518157000	18.309059000	12.548753000
C	14.043111000	19.163065000	10.628480000
H	13.812005000	20.143349000	11.042754000
C	10.538742000	22.467870000	8.285998000
C	9.245667000	21.952054000	8.101932000
H	8.886457000	21.157407000	8.758267000
C	8.420181000	22.446868000	7.090460000
H	7.415733000	22.042724000	6.964696000
C	8.880990000	23.456745000	6.244738000
H	8.237356000	23.845264000	5.455509000
C	10.167994000	23.975528000	6.415078000
H	10.525876000	24.772253000	5.762341000
C	10.990576000	23.484817000	7.428516000
H	11.985125000	23.918882000	7.550718000
C	11.755335000	23.073555000	10.887468000
C	11.558097000	24.437826000	10.632911000
H	11.173084000	24.766584000	9.667903000

C	11.827132000	25.381804000	11.626531000
H	11.655662000	26.439645000	11.425614000
C	12.295857000	24.975032000	12.878450000
H	12.491616000	25.714986000	13.654656000
C	12.477857000	23.615634000	13.145370000
H	12.806662000	23.291046000	14.133082000
C	12.198739000	22.669323000	12.158392000
H	12.295340000	21.604737000	12.385446000
P	16.802342000	20.751528000	8.094916000
P	18.979518000	19.298567000	9.659488000
C	17.224553000	19.226055000	8.943831000
H	17.089418000	18.393941000	8.239353000
H	16.524707000	19.070876000	9.773850000
C	18.077909000	21.171784000	6.911613000
C	19.047346000	22.127965000	7.258545000
H	18.968848000	22.680870000	8.193841000
C	20.122995000	22.353352000	6.400979000
H	20.875256000	23.095912000	6.665123000
C	20.238994000	21.626413000	5.214514000
H	21.084466000	21.803860000	4.550144000
C	19.279019000	20.668707000	4.874637000
H	19.377212000	20.097102000	3.952546000
C	18.197395000	20.436296000	5.719500000
H	17.466664000	19.672283000	5.451349000
C	16.524456000	22.096539000	9.247309000
C	16.259285000	23.376871000	8.727778000
H	16.357243000	23.571829000	7.658305000
C	15.872080000	24.404206000	9.586073000
H	15.666778000	25.396380000	9.185217000

C	15.743121000	24.160243000	10.956595000
H	15.416496000	24.958286000	11.622670000
C	16.035587000	22.896717000	11.476732000
H	15.954454000	22.716420000	12.548112000
C	16.430785000	21.862107000	10.628281000
H	16.660838000	20.881663000	11.042750000
C	19.935829000	18.555839000	8.284179000
C	21.229002000	19.070904000	8.098773000
H	21.589350000	19.865352000	8.754727000
C	22.053153000	18.575615000	7.086439000
H	23.057718000	18.979151000	6.959651000
C	21.590868000	17.566032000	6.241172000
H	22.233444000	17.177167000	5.451251000
C	20.303732000	17.048010000	6.412830000
H	19.944684000	16.251543000	5.760417000
C	19.482481000	17.539200000	7.427110000
H	18.487755000	17.105819000	7.550286000
C	18.721430000	17.951472000	10.887274000
C	18.916531000	16.586877000	10.632846000
H	19.299265000	16.257304000	9.667224000
C	18.648309000	15.643579000	11.627356000
H	18.818174000	14.585472000	11.426484000
C	18.182563000	16.051332000	12.880042000
H	17.987499000	15.311895000	13.656913000
C	18.002666000	17.411055000	13.146809000
H	17.676172000	17.736396000	14.135042000
C	18.280982000	18.356648000	12.158945000
H	18.186106000	19.421423000	12.385867000

Atomic coordinates of [CH(dppm)$_2$]Cl (**12a**) optimized (PBE-D3(BJ)/def2-TZVPP) in the ground state S_0 in the gas phase.

P	14.014030000	20.560295000	7.824260000
P	11.964642000	21.367264000	10.241484000
C	15.542723000	21.080511000	7.227316000
C	13.466340000	21.681522000	9.156034000
H	13.393536000	22.660377000	8.657836000
H	14.328960000	21.760579000	9.835964000
C	12.832511000	20.610767000	6.461982000
C	11.793270000	19.671998000	6.398737000
H	11.717398000	18.887476000	7.151098000
C	10.854011000	19.747097000	5.371626000
H	10.044107000	19.019556000	5.328310000
C	10.949100000	20.751299000	4.406311000
H	10.214678000	20.804464000	3.602504000
C	11.981143000	21.690279000	4.470105000
H	12.054885000	22.476050000	3.718356000
C	12.920702000	21.626566000	5.497931000
H	13.719152000	22.367013000	5.552567000
C	14.082899000	18.859978000	8.464035000
C	14.510145000	17.864869000	7.566503000
H	14.650048000	18.109564000	6.512155000
C	14.785515000	16.579861000	8.026984000
H	15.111314000	15.811163000	7.325739000
C	14.661898000	16.281774000	9.389362000
H	14.907385000	15.284499000	9.754243000
C	14.227644000	17.262103000	10.280720000
H	14.134557000	17.033721000	11.342084000

C	13.927210000	18.546955000	9.821017000
H	13.573730000	19.300967000	10.524646000
C	10.584156000	21.504895000	9.041231000
C	9.591743000	20.515694000	9.092121000
H	9.674252000	19.713150000	9.827366000
C	8.503550000	20.556804000	8.216329000
H	7.732601000	19.787766000	8.270678000
C	8.405805000	21.583543000	7.277307000
H	7.558622000	21.617913000	6.591769000
C	9.392095000	22.574026000	7.217910000
H	9.314769000	23.378020000	6.485442000
C	10.470972000	22.538805000	8.098161000
H	11.222649000	23.328767000	8.059025000
C	11.941814000	23.013565000	11.066897000
C	10.748725000	23.739573000	11.207576000
H	9.830385000	23.371404000	10.749960000
C	10.728154000	24.938168000	11.923159000
H	9.792740000	25.490217000	12.019201000
C	11.895502000	25.431094000	12.508453000
H	11.877428000	26.368837000	13.063769000
C	13.085619000	24.710807000	12.382992000
H	14.003078000	25.082190000	12.841116000
C	13.107764000	23.508650000	11.676840000
H	14.048962000	22.959674000	11.608746000
P	16.931202000	21.255648000	8.224293000
P	18.763974000	19.402098000	10.052839000
C	17.070125000	19.842245000	9.371816000
H	16.643317000	18.979578000	8.847541000
H	16.422395000	20.049749000	10.234472000

C	18.395489000	21.350988000	7.173125000
C	19.464316000	22.188480000	7.521572000
H	19.392653000	22.835165000	8.395702000
C	20.619081000	22.196925000	6.738641000
H	21.447800000	22.852176000	7.006451000
C	20.709472000	21.373176000	5.615500000
H	21.613473000	21.381759000	5.006284000
C	19.644722000	20.536826000	5.269386000
H	19.718994000	19.887418000	4.397516000
C	18.487516000	20.522032000	6.044337000
H	17.659879000	19.861624000	5.786151000
C	16.907306000	22.740251000	9.281620000
C	16.239298000	23.876768000	8.801483000
H	15.761832000	23.838785000	7.820922000
C	16.165093000	25.029146000	9.581755000
H	15.638922000	25.906723000	9.206365000
C	16.754037000	25.053151000	10.848879000
H	16.689250000	25.952359000	11.461866000
C	17.425872000	23.927319000	11.329943000
H	17.892392000	23.947084000	12.315054000
C	17.508930000	22.772151000	10.549602000
H	18.059844000	21.906374000	10.920942000
C	19.455261000	18.461037000	8.634745000
C	20.796073000	18.703188000	8.305345000
H	21.367720000	19.426291000	8.889241000
C	21.396558000	18.036132000	7.234985000
H	22.440122000	18.234224000	6.989487000
C	20.660103000	17.122326000	6.480940000
H	21.127141000	16.600484000	5.645250000

C	19.321428000	16.873779000	6.800669000
H	18.745211000	16.155014000	6.216528000
C	18.723059000	17.537240000	7.870479000
H	17.681610000	17.321217000	8.115154000
C	18.145999000	18.084357000	11.187457000
C	18.433651000	16.721171000	11.038237000
H	19.049374000	16.380832000	10.206344000
C	17.933480000	15.789211000	11.951988000
H	18.168670000	14.732014000	11.823673000
C	17.142125000	16.204336000	13.023483000
H	16.754094000	15.474766000	13.734492000
C	16.867511000	17.564753000	13.192927000
H	16.268187000	17.901737000	14.039457000
C	17.374637000	18.495996000	12.288747000
H	17.172288000	19.558527000	12.447575000
H	15.636843000	21.312089000	6.169830000

Atomic coordinates of [CH(dppm)$_2$]Cl (**12b**) optimized (PBE-D3(BJ)/def2-TZVPP) in the ground state S_0 in the gas phase.

P	13.831673000	20.308138000	7.165529000
P	11.994954000	20.880642000	9.503136000
C	15.473075000	20.906229000	6.546917000
C	13.368144000	21.310554000	8.440426000
H	13.620271000	22.363007000	8.278490000
C	12.834393000	20.214649000	5.634038000
C	11.451075000	20.062081000	5.827663000
H	11.048799000	20.001658000	6.841862000
C	10.593596000	19.998852000	4.731587000

H	9.521857000	19.880747000	4.890986000
C	11.105405000	20.097324000	3.434266000
H	10.432247000	20.055766000	2.577973000
C	12.477872000	20.253035000	3.234735000
H	12.878807000	20.331576000	2.223994000
C	13.343121000	20.307893000	4.330202000
H	14.412700000	20.422368000	4.150037000
C	14.077279000	18.570361000	7.643761000
C	14.372199000	17.622099000	6.651542000
H	14.343180000	17.904100000	5.597395000
C	14.683293000	16.309928000	7.009173000
H	14.912399000	15.577457000	6.235045000
C	14.685320000	15.934064000	8.355586000
H	14.925973000	14.907656000	8.633497000
C	14.367349000	16.868904000	9.344183000
H	14.365756000	16.582204000	10.396046000
C	14.065184000	18.183835000	8.991420000
H	13.829709000	18.915337000	9.763403000
C	10.852168000	22.313293000	9.308325000
C	10.004784000	22.696051000	10.361714000
H	10.082949000	22.200115000	11.330483000
C	9.072247000	23.718096000	10.187891000
H	8.429264000	24.007912000	11.019844000
C	8.961200000	24.371047000	8.957176000
H	8.230743000	25.169078000	8.823220000
C	9.793870000	23.994093000	7.902349000
H	9.716112000	24.497636000	6.937579000
C	10.732591000	22.974610000	8.076176000
H	11.382374000	22.691040000	7.246838000

C	12.663427000	21.234016000	11.191983000
C	12.216103000	20.460020000	12.271856000
H	11.487472000	19.665963000	12.094794000
C	12.693304000	20.697205000	13.565348000
H	12.328222000	20.095127000	14.398619000
C	13.637736000	21.699822000	13.789394000
H	14.016609000	21.880592000	14.795480000
C	14.095922000	22.472450000	12.716309000
H	14.828258000	23.262988000	12.889352000
C	13.609261000	22.243054000	11.429783000
H	13.962005000	22.855239000	10.599323000
P	16.646187000	21.399566000	7.852006000
P	18.011788000	20.109453000	10.403398000
C	16.587029000	20.180382000	9.176616000
H	16.456933000	19.200538000	8.698700000
H	15.651330000	20.406692000	9.709807000
C	18.273098000	21.432720000	7.072034000
C	19.113884000	22.541549000	7.243862000
H	18.774522000	23.403289000	7.817737000
C	20.387727000	22.537428000	6.674050000
H	21.039113000	23.401146000	6.805120000
C	20.822388000	21.434600000	5.938291000
H	21.817752000	21.435146000	5.494092000
C	19.986850000	20.326392000	5.770765000
H	20.330557000	19.459467000	5.207826000
C	18.714384000	20.321269000	6.334219000
H	18.082953000	19.439992000	6.215341000
C	16.266524000	23.045677000	8.462427000
C	15.717365000	24.013966000	7.605681000

H	15.483839000	23.778653000	6.567422000
C	15.451672000	25.293934000	8.087006000
H	15.009498000	26.037670000	7.424880000
C	15.746175000	25.619343000	9.413942000
H	15.532303000	26.620413000	9.787849000
C	16.315413000	24.665003000	10.258948000
H	16.553774000	24.919154000	11.291590000
C	16.576941000	23.377372000	9.791342000
H	17.024688000	22.637120000	10.455804000
C	19.207173000	19.100311000	9.437667000
C	20.528079000	19.561406000	9.360637000
H	20.802344000	20.487737000	9.868067000
C	21.488034000	18.846329000	8.639623000
H	22.513337000	19.213395000	8.589275000
C	21.133958000	17.663773000	7.989735000
H	21.882986000	17.101555000	7.431396000
C	19.818166000	17.193369000	8.063083000
H	19.542082000	16.262397000	7.566422000
C	18.861198000	17.905355000	8.783898000
H	17.844238000	17.512752000	8.855193000
C	17.235003000	18.859346000	11.514264000
C	17.895123000	17.671879000	11.864925000
H	18.871446000	17.441843000	11.439566000
C	17.305274000	16.770799000	12.753845000
H	17.830596000	15.851204000	13.013450000
C	16.052122000	17.042750000	13.304453000
H	15.590816000	16.335702000	13.994165000
C	15.399486000	18.234588000	12.979292000
H	14.428375000	18.471503000	13.413071000

C	15.989550000	19.143233000	12.101075000
H	15.465607000	20.077177000	11.886674000
H	15.955908000	20.116552000	5.955588000
H	15.324490000	21.764733000	5.877520000

Atomic coordinates of $(CDP(CH_2PPh_2)_2$ (**13a**) optimized (PBE-D3(BJ)/def2-TZVPP) in the ground state S_0 in the gas phase.

P	14.542831000	19.591548000	7.987285000
P	13.534671000	19.013857000	10.835411000
C	15.466268000	20.831018000	7.190943000
C	14.257726000	20.081251000	9.607154000
H	13.908666000	21.120624000	9.591552000
C	13.053923000	19.298796000	6.957488000
C	12.319817000	18.115467000	7.134436000
H	12.715253000	17.323628000	7.772340000
C	11.079149000	17.956346000	6.521110000
H	10.510399000	17.040219000	6.682657000
C	10.558369000	18.974596000	5.717769000
H	9.585149000	18.850998000	5.241345000
C	11.283297000	20.154102000	5.534858000
H	10.880666000	20.951863000	4.908891000
C	12.522391000	20.318851000	6.157118000
H	13.080953000	21.248388000	6.035838000
C	15.401527000	17.970910000	7.929003000
C	15.613674000	17.375368000	6.676050000
H	15.166015000	17.819873000	5.784986000
C	16.393663000	16.225259000	6.560955000
H	16.552079000	15.772464000	5.581279000

C	16.973288000	15.656460000	7.699257000
H	17.588911000	14.760591000	7.609867000
C	16.761075000	16.239521000	8.949583000
H	17.206562000	15.798695000	9.842212000
C	15.979332000	17.390856000	9.065815000
H	15.802385000	17.838500000	10.044147000
C	11.718227000	18.908298000	10.430250000
C	11.046153000	17.698861000	10.650059000
H	11.588642000	16.863003000	11.098169000
C	9.705378000	17.548366000	10.281837000
H	9.195629000	16.599242000	10.456502000
C	9.025602000	18.607112000	9.678228000
H	7.983849000	18.489217000	9.376724000
C	9.689293000	19.817759000	9.451117000
H	9.165149000	20.644946000	8.969389000
C	11.023137000	19.964962000	9.825772000
H	11.544437000	20.903393000	9.626409000
C	13.419624000	20.154855000	12.281943000
C	12.800784000	19.679137000	13.452035000
H	12.372783000	18.674220000	13.460776000
C	12.723922000	20.471744000	14.595636000
H	12.237476000	20.083386000	15.491857000
C	13.273458000	21.759020000	14.597824000
H	13.220069000	22.377553000	15.494596000
C	13.892561000	22.240606000	13.444366000
H	14.331589000	23.239672000	13.431140000
C	13.965001000	21.446252000	12.296620000
H	14.467845000	21.831870000	11.410716000
P	16.703636000	21.596633000	8.081462000

P	19.229234000	20.886582000	9.978665000
C	17.725610000	20.385943000	8.992363000
H	17.027715000	19.777929000	9.582149000
C	17.848388000	22.383647000	6.907301000
C	18.212316000	23.731451000	7.004547000
H	17.781862000	24.355432000	7.787475000
C	19.125224000	24.275797000	6.098689000
H	19.402384000	25.327322000	6.178255000
C	19.680866000	23.478353000	5.097366000
H	20.394920000	23.905600000	4.392538000
C	19.320499000	22.131448000	4.997115000
H	19.750991000	21.504947000	4.215312000
C	18.407294000	21.584477000	5.897097000
H	18.119261000	20.535079000	5.814087000
C	16.136665000	22.911414000	9.210300000
C	14.866190000	23.457255000	8.975539000
H	14.271571000	23.057046000	8.152060000
C	14.370838000	24.466068000	9.801118000
H	13.376666000	24.875382000	9.619887000
C	15.139778000	24.936052000	10.868851000
H	14.746535000	25.714334000	11.523802000
C	16.411003000	24.405030000	11.099356000
H	17.015062000	24.767198000	11.931677000
C	16.914023000	23.402424000	10.269203000
H	17.916430000	23.012762000	10.450650000
C	20.195578000	19.339849000	9.692750000
C	21.187906000	19.371663000	8.701521000
H	21.399073000	20.310798000	8.185490000
C	21.899469000	18.215440000	8.373704000

H	22.668742000	18.251678000	7.601205000
C	21.632234000	17.018711000	9.041553000
H	22.190407000	16.116033000	8.790501000
C	20.651785000	16.981935000	10.037147000
H	20.440954000	16.048908000	10.561854000
C	19.936189000	18.134345000	10.361538000
H	19.170770000	18.100922000	11.137863000
C	18.667672000	20.630707000	11.710375000
C	19.664260000	20.707185000	12.700965000
H	20.701715000	20.885555000	12.408985000
C	19.344188000	20.542426000	14.046611000
H	20.131444000	20.592163000	14.800140000
C	18.017025000	20.319718000	14.428437000
H	17.762885000	20.195524000	15.481650000
C	17.017630000	20.266867000	13.457315000
H	15.977082000	20.110030000	13.742138000
C	17.338288000	20.421297000	12.104663000
H	16.529430000	20.381832000	11.371343000
H	15.473473000	20.936440000	6.108347000
H	18.097855000	19.738635000	8.185214000

Atomic coordinates of ($CDP(CH_2PPh_2)_2$ (**13b**) optimized (PBE-D3(BJ)/def2-TZVPP) in the ground state S_0 in the gas phase.

P	14.396010000	19.160942000	8.311562000
P	12.265397000	19.353041000	10.446719000
C	15.456800000	20.301422000	7.333806000
C	13.615716000	20.027438000	9.519276000
H	13.971529000	21.042684000	9.680976000

C	13.251597000	18.440205000	7.077831000
C	13.346476000	17.134273000	6.581731000
H	14.137926000	16.472507000	6.932287000
C	12.408808000	16.668391000	5.656936000
H	12.479659000	15.646006000	5.284175000
C	11.378803000	17.503021000	5.220180000
H	10.646227000	17.134573000	4.501164000
C	11.276718000	18.805794000	5.718087000
H	10.462958000	19.455556000	5.394157000
C	12.203256000	19.270117000	6.649010000
H	12.103100000	20.265801000	7.086000000
C	15.499105000	17.781013000	8.800211000
C	16.427690000	17.198971000	7.922423000
H	16.477300000	17.513115000	6.878611000
C	17.320092000	16.232581000	8.385625000
H	18.060611000	15.808565000	7.707106000
C	17.281647000	15.830888000	9.723511000
H	17.989800000	15.085875000	10.087860000
C	16.347576000	16.392847000	10.596284000
H	16.321585000	16.088313000	11.642738000
C	15.462366000	17.369345000	10.138118000
H	14.757794000	17.847464000	10.820564000
C	10.951245000	20.629399000	10.153005000
C	9.879766000	20.297726000	9.311046000
H	9.803743000	19.278729000	8.925487000
C	8.922625000	21.254376000	8.961359000
H	8.092980000	20.980546000	8.307297000
C	9.022126000	22.555930000	9.457393000
H	8.273706000	23.303295000	9.190360000

C	10.080708000	22.893953000	10.307192000
H	10.159451000	23.908045000	10.703758000
C	11.035653000	21.938129000	10.653387000
H	11.860089000	22.208630000	11.315618000
C	12.709420000	19.774058000	12.188211000
C	11.748921000	19.605127000	13.200976000
H	10.744773000	19.264301000	12.938613000
C	12.059714000	19.878747000	14.531866000
H	11.299770000	19.747382000	15.303866000
C	13.342113000	20.318135000	14.879740000
H	13.585149000	20.530970000	15.921538000
C	14.306745000	20.481218000	13.884294000
H	15.309314000	20.827643000	14.141265000
C	13.991660000	20.210568000	12.549403000
H	14.740663000	20.349365000	11.767698000
P	16.666131000	21.323708000	8.270796000
P	19.148382000	20.908306000	9.929311000
C	17.614647000	20.339611000	9.247411000
H	17.281034000	19.310257000	9.359391000
C	17.615273000	22.189528000	6.967319000
C	17.509617000	23.559468000	6.698140000
H	16.826172000	24.178841000	7.278145000
C	18.300418000	24.139381000	5.703145000
H	18.223578000	25.209487000	5.507718000
C	19.192547000	23.355906000	4.969611000
H	19.810804000	23.812554000	4.195923000
C	19.305446000	21.988237000	5.239147000
H	20.014872000	21.374997000	4.682482000
C	18.527682000	21.409139000	6.239021000

H	18.650165000	20.354794000	6.494705000
C	15.667330000	22.632556000	9.077019000
C	14.636522000	23.322090000	8.418636000
H	14.436903000	23.142964000	7.360803000
C	13.837212000	24.223439000	9.120935000
H	13.017764000	24.732366000	8.612901000
C	14.071822000	24.453547000	10.479389000
H	13.436301000	25.147770000	11.030243000
C	15.108900000	23.785625000	11.133781000
H	15.287628000	23.955742000	12.195680000
C	15.901203000	22.873099000	10.436242000
H	16.684874000	22.307924000	10.943996000
C	20.351974000	19.697182000	9.200182000
C	21.257742000	20.155483000	8.232965000
H	21.288305000	21.221401000	7.997528000
C	22.106879000	19.264683000	7.569605000
H	22.808129000	19.636563000	6.820537000
C	22.064574000	17.902445000	7.872741000
H	22.729473000	17.205733000	7.360364000
C	21.172471000	17.436522000	8.844917000
H	21.141231000	16.373464000	9.091704000
C	20.325448000	18.326957000	9.503920000
H	19.630605000	17.957781000	10.260554000
C	19.098414000	20.262799000	11.657246000
C	20.267789000	20.312391000	12.436526000
H	21.190740000	20.697447000	11.997000000
C	20.265503000	19.865195000	13.756458000
H	21.184077000	19.906303000	14.344060000
C	19.089802000	19.367109000	14.329321000

H	19.088182000	19.018325000	15.362772000
C	17.920763000	19.320421000	13.568545000
H	16.998208000	18.929735000	14.001545000
C	17.926126000	19.765038000	12.242930000
H	17.015313000	19.717309000	11.643983000
H	14.783056000	20.966450000	6.778032000
H	16.031314000	19.718614000	6.602271000

Atomic coordinates of (CDP(CH_2PPh_2)$_2$ (**13c**) optimized (PBE-D3(BJ)/def2-TZVPP) in the ground state S_0 in the gas phase.

P	13.813093000	20.340547000	8.223191000
P	11.446434000	21.961912000	9.624071000
C	15.238316000	20.513591000	7.381598000
C	13.204601000	21.897489000	9.007276000
H	13.397882000	22.673657000	8.254318000
H	13.885242000	22.109428000	9.842756000
C	12.469504000	19.820265000	7.107935000
C	11.365284000	19.086516000	7.562042000
H	11.306155000	18.783067000	8.607678000
C	10.343941000	18.741192000	6.675062000
H	9.491143000	18.161183000	7.030391000
C	10.410649000	19.143462000	5.339900000
H	9.607589000	18.880216000	4.650182000
C	11.508357000	19.880611000	4.886338000
H	11.562726000	20.193342000	3.842819000
C	12.540935000	20.209256000	5.764274000
H	13.424107000	20.755561000	5.427457000
C	13.795768000	19.094437000	9.571687000

C	13.991077000	17.749228000	9.211000000
H	14.010718000	17.479808000	8.153460000
C	14.182017000	16.771703000	10.184822000
H	14.335391000	15.733183000	9.889283000
C	14.191877000	17.123043000	11.538877000
H	14.352666000	16.359342000	12.300392000
C	13.997533000	18.454499000	11.908897000
H	13.996229000	18.735861000	12.962972000
C	13.794356000	19.433664000	10.932041000
H	13.638567000	20.465770000	11.242767000
C	10.566504000	22.571862000	8.123000000
C	9.366493000	21.943013000	7.767686000
H	9.000151000	21.116047000	8.377442000
C	8.652861000	22.352382000	6.638547000
H	7.724905000	21.846066000	6.370102000
C	9.133610000	23.398952000	5.852055000
H	8.582008000	23.717110000	4.966554000
C	10.327117000	24.039158000	6.200653000
H	10.705697000	24.860401000	5.590529000
C	11.036036000	23.629431000	7.328413000
H	11.959043000	24.145808000	7.597559000
C	11.533413000	23.501216000	10.639388000
C	10.555100000	24.504736000	10.544129000
H	9.774203000	24.428186000	9.787029000
C	10.577849000	25.608479000	11.398907000
H	9.811678000	26.379064000	11.301206000
C	11.576117000	25.732752000	12.366926000
H	11.595901000	26.598008000	13.030173000
C	12.549253000	24.736626000	12.477598000

H	13.336009000	24.818867000	13.229133000
C	12.523205000	23.630629000	11.629195000
H	13.295859000	22.868043000	11.741475000
P	16.663122000	20.685357000	8.224141000
P	19.028159000	19.062457000	9.625993000
C	17.270655000	19.127613000	9.007367000
H	17.078177000	18.352169000	8.253457000
H	16.589079000	18.914910000	9.841879000
C	18.007509000	21.206056000	7.110043000
C	19.111732000	21.939085000	7.565306000
H	19.170417000	22.241679000	8.611215000
C	20.133639000	22.284802000	6.679130000
H	20.986435000	22.864260000	7.035366000
C	20.067490000	21.883632000	5.343610000
H	20.870988000	22.147183000	4.654517000
C	18.969780000	21.147198000	4.888892000
H	18.915843000	20.835332000	3.845092000
C	17.936643000	20.818179000	5.766031000
H	17.053459000	20.272446000	5.428321000
C	16.680004000	21.930334000	9.573682000
C	16.485777000	23.275987000	9.214080000
H	16.467374000	23.546438000	8.156782000
C	16.294349000	24.252679000	10.188642000
H	16.141826000	25.291562000	9.893936000
C	16.282927000	23.900064000	11.542350000
H	16.121739000	24.663118000	12.304426000
C	16.476192000	22.568150000	11.911298000
H	16.476275000	22.285778000	12.965104000
C	16.679842000	21.589815000	10.933718000

H	16.834709000	20.557321000	11.243619000
C	19.909449000	18.452870000	8.125556000
C	21.109517000	19.082098000	7.771114000
H	21.475180000	19.909154000	8.381153000
C	21.824071000	18.672979000	6.642466000
H	22.752057000	19.179594000	6.374689000
C	21.344213000	17.626270000	5.855616000
H	21.896546000	17.308306000	4.970501000
C	20.150669000	16.985669000	6.203364000
H	19.772792000	16.164306000	5.592966000
C	19.440825000	17.395160000	7.330623000
H	18.517805000	16.878473000	7.599133000
C	18.939674000	17.522685000	10.640477000
C	19.918215000	16.519294000	10.546170000
H	20.700224000	16.596289000	9.790260000
C	19.894282000	15.415111000	11.400342000
H	20.660660000	14.644637000	11.303385000
C	18.894572000	15.290252000	12.366799000
H	18.873862000	14.424647000	13.029562000
C	17.921182000	16.286231000	12.476522000
H	17.133298000	16.203525000	13.226823000
C	17.948402000	17.392669000	11.628725000
H	17.175498000	18.155111000	11.740242000

Atomic coordinates of $(CDP(CH_2PPh_2)_2$ (**13d**) optimized (PBE-D3(BJ)/def2-TZVPP) in the ground state S_0 in the gas phase.

P	14.492460000	19.873261000	7.919310000
P	12.248591000	20.876674000	10.079552000

C	15.114506000	20.395983000	6.172129000
C	13.998313000	20.879086000	9.422840000
H	14.364860000	21.893303000	9.215342000
H	14.659600000	20.490275000	10.210095000
C	12.703303000	19.891311000	7.139768000
C	11.950855000	18.765775000	6.778046000
H	12.310845000	17.765721000	7.019378000
C	10.729792000	18.895001000	6.106948000
H	10.163086000	18.000088000	5.843533000
C	10.235747000	20.156824000	5.775133000
H	9.281684000	20.256772000	5.256007000
C	10.981183000	21.290660000	6.106794000
H	10.612545000	22.286704000	5.856662000
C	12.199198000	21.150840000	6.770939000
H	12.766470000	22.054438000	7.009832000
C	14.480398000	18.082526000	8.309858000
C	15.436462000	17.236503000	7.737053000
H	16.184041000	17.657454000	7.063494000
C	15.472929000	15.881590000	8.069367000
H	16.218635000	15.230141000	7.611778000
C	14.573283000	15.368262000	9.008599000
H	14.617546000	14.315416000	9.289449000
C	13.618241000	16.208410000	9.585459000
H	12.913803000	15.814781000	10.319356000
C	13.555173000	17.555478000	9.222129000
H	12.786132000	18.201237000	9.651539000
C	11.579204000	22.500848000	9.520397000
C	10.197281000	22.575729000	9.295734000
H	9.587411000	21.682696000	9.443135000

C	9.606411000	23.763904000	8.861587000
H	8.530108000	23.803142000	8.688936000
C	10.395372000	24.892663000	8.630835000
H	9.938774000	25.817911000	8.277157000
C	11.774041000	24.830763000	8.852977000
H	12.399729000	25.706600000	8.674384000
C	12.358480000	23.647112000	9.304109000
H	13.434727000	23.626290000	9.481421000
C	12.613896000	21.246596000	11.852267000
C	12.108211000	22.373949000	12.518686000
H	11.543352000	23.123534000	11.964944000
C	12.324396000	22.549399000	13.887551000
H	11.924758000	23.434519000	14.385097000
C	13.049723000	21.604649000	14.614287000
H	13.220356000	21.743720000	15.682415000
C	13.555845000	20.477735000	13.961087000
H	14.130190000	19.732929000	14.511770000
C	13.334784000	20.295875000	12.597221000
H	13.737006000	19.403734000	12.111859000
P	16.585175000	21.137493000	6.890729000
P	17.570465000	20.486363000	9.639185000
C	16.376919000	20.224121000	8.306422000
C	18.118671000	20.928424000	5.935406000
C	18.864870000	22.013714000	5.457758000
H	18.510309000	23.031335000	5.623365000
C	20.067616000	21.791395000	4.783371000
H	20.648213000	22.639846000	4.419334000
C	20.528785000	20.489395000	4.583021000
H	21.472128000	20.318639000	4.063143000

C	19.788512000	19.403923000	5.062280000
H	20.156775000	18.386653000	4.928918000
C	18.592128000	19.621884000	5.738767000
H	18.035880000	18.783358000	6.159357000
C	16.265691000	22.943079000	6.930757000
C	15.678658000	23.609322000	5.843124000
H	15.526288000	23.084426000	4.898217000
C	15.276381000	24.940998000	5.965966000
H	14.807277000	25.445904000	5.120646000
C	15.470043000	25.621867000	7.169929000
H	15.155866000	26.662068000	7.264434000
C	16.064000000	24.967559000	8.253563000
H	16.216309000	25.496115000	9.195462000
C	16.453089000	23.632276000	8.139755000
H	16.892610000	23.104974000	8.990024000
C	19.004837000	19.422999000	9.113725000
C	20.227718000	20.067311000	8.883709000
H	20.302796000	21.140630000	9.070467000
C	21.335082000	19.357708000	8.409290000
H	22.275997000	19.878308000	8.224895000
C	21.233698000	17.987741000	8.168246000
H	22.096085000	17.429468000	7.801210000
C	20.018985000	17.332019000	8.399807000
H	19.935430000	16.258639000	8.218815000
C	18.914048000	18.045152000	8.862880000
H	17.970076000	17.526146000	9.027872000
C	17.000309000	19.539734000	11.120316000
C	17.312491000	20.111447000	12.365334000
H	17.795980000	21.089844000	12.389657000

C	17.020421000	19.445178000	13.556398000
H	17.275633000	19.905581000	14.511849000
C	16.391546000	18.198970000	13.523670000
H	16.158596000	17.676483000	14.452718000
C	16.055682000	17.627890000	12.293782000
H	15.556680000	16.658317000	12.254236000
C	16.362829000	18.289408000	11.103570000
H	16.104042000	17.823939000	10.156848000
H	15.323003000	19.480979000	5.601129000
H	14.432071000	21.036849000	5.606083000

Atomic coordinates of (CH(CHPPh$_2$)$_2$ (**20a**) optimized (PBE-D3(BJ)/def2-TZVPP) in the ground state S$_0$ in the gas phase.

P	13.686917000	20.596651000	8.541027000
P	11.268477000	22.381783000	8.942494000
C	15.237925000	20.512105000	7.791811000
C	12.973062000	22.156420000	8.605230000
H	13.652245000	22.977877000	8.367969000
C	12.544322000	19.502549000	7.584986000
C	12.052740000	18.269204000	8.030721000
H	12.324433000	17.902322000	9.020063000
C	11.196662000	17.513863000	7.225233000
H	10.810019000	16.561914000	7.594037000
C	10.827779000	17.975768000	5.960476000
H	10.156023000	17.385901000	5.334515000
C	11.310593000	19.207654000	5.508809000
H	11.014255000	19.588852000	4.530081000
C	12.151769000	19.967846000	6.319425000

H	12.489789000	20.951768000	5.990766000
C	13.808332000	19.753270000	10.175709000
C	14.418308000	18.495159000	10.297783000
H	14.835724000	18.014964000	9.413386000
C	14.516624000	17.876189000	11.544624000
H	14.998850000	16.900707000	11.628336000
C	14.013929000	18.511737000	12.683258000
H	14.105827000	18.034658000	13.660619000
C	13.403373000	19.762354000	12.567570000
H	13.015032000	20.267656000	13.453301000
C	13.296694000	20.376122000	11.317998000
H	12.829215000	21.356297000	11.214840000
C	10.491442000	23.104183000	7.413846000
C	9.095244000	23.090535000	7.267485000
H	8.479862000	22.672158000	8.067827000
C	8.487777000	23.571931000	6.104875000
H	7.400157000	23.548791000	6.009595000
C	9.271552000	24.062149000	5.057551000
H	8.801253000	24.428264000	4.143254000
C	10.664553000	24.061669000	5.182774000
H	11.285759000	24.434073000	4.365298000
C	11.265565000	23.583876000	6.348293000
H	12.353851000	23.558807000	6.441340000
C	11.212197000	23.879052000	10.031190000
C	10.071083000	24.693799000	10.147716000
H	9.206880000	24.507620000	9.508223000
C	10.031041000	25.751752000	11.055772000
H	9.135648000	26.374066000	11.118259000
C	11.127822000	26.021037000	11.881086000

H	11.094454000	26.848147000	12.592097000
C	12.267839000	25.221044000	11.775728000
H	13.137285000	25.421352000	12.406286000
C	12.310776000	24.168520000	10.859355000
H	13.208274000	23.555363000	10.762090000
P	16.788644000	20.428050000	8.541602000
P	19.207500000	18.643176000	8.942303000
C	17.502752000	18.868389000	8.605566000
H	16.823437000	18.046660000	8.369640000
C	17.932177000	21.522523000	7.587035000
C	18.423468000	22.755621000	8.033779000
H	18.151163000	23.121931000	9.023165000
C	19.280060000	23.511428000	7.229275000
H	19.666454000	24.463170000	7.598873000
C	19.649761000	23.050248000	5.964494000
H	20.321900000	23.640480000	5.339290000
C	19.167257000	21.818609000	5.511825000
H	19.464194000	21.437984000	4.533055000
C	18.325539000	21.057969000	6.321458000
H	17.987666000	20.074281000	5.991961000
C	16.665676000	21.271064000	10.176330000
C	16.055717000	22.529207000	10.298268000
H	15.638970000	23.009646000	9.413699000
C	15.956599000	23.147863000	11.545213000
H	15.474416000	24.123375000	11.628837000
C	16.458543000	22.512042000	12.684013000
H	16.366038000	22.988909000	13.661420000
C	17.069157000	21.261443000	12.568440000
H	17.456931000	20.755943000	13.454307000

C	17.176609000	20.647962000	11.318798000
H	17.644249000	19.667859000	11.215667000
C	19.983969000	17.920388000	7.413520000
C	21.380047000	17.934949000	7.266102000
H	21.995738000	18.354209000	8.065743000
C	21.986986000	17.453377000	6.103288000
H	23.074519000	17.477246000	6.007182000
C	21.202782000	16.962079000	5.056799000
H	21.672661000	16.595813000	4.142346000
C	19.809869000	16.961695000	5.183040000
H	19.188317000	16.588435000	4.366219000
C	19.209391000	17.439683000	6.348752000
H	18.121150000	17.464075000	6.442585000
C	19.264022000	17.146138000	10.031374000
C	20.405495000	16.331966000	10.148559000
H	21.269873000	16.518432000	9.509399000
C	20.445685000	15.274203000	11.056826000
H	21.341359000	14.652344000	11.119806000
C	19.348710000	15.004551000	11.881761000
H	19.382194000	14.177603000	12.592955000
C	18.208348000	15.803959000	11.775767000
H	17.338746000	15.603381000	12.406025000
C	18.165261000	16.856267000	10.859154000
H	17.267472000	17.468912000	10.761405000
H	15.237899000	20.509887000	6.701213000

Atomic coordinates of $(CH(CHPPh_2)_2$ (**20b**) optimized (PBE-D3(BJ)/def2-TZVPP) in the ground state S_0 in the gas phase.

P	14.200012000	20.721706000	8.299698000
P	11.604060000	21.904444000	9.411170000
C	15.796958000	21.061303000	7.879669000
C	13.182986000	22.068295000	8.680289000
H	13.482969000	22.990654000	8.172443000
C	13.505869000	19.732520000	6.897169000
C	12.180057000	19.275707000	6.928591000
H	11.561922000	19.483229000	7.805582000
C	11.645981000	18.573161000	5.848558000
H	10.612141000	18.224799000	5.886208000
C	12.427401000	18.331485000	4.712792000
H	12.005837000	17.790392000	3.863407000
C	13.746545000	18.791259000	4.670304000
H	14.360398000	18.609020000	3.784970000
C	14.283635000	19.481030000	5.761152000
H	15.314169000	19.845281000	5.770363000
C	14.062617000	19.515198000	9.691938000
C	14.180404000	18.132303000	9.484264000
H	14.186998000	17.742006000	8.465003000
C	14.290032000	17.254388000	10.565553000
H	14.394441000	16.182813000	10.387091000
C	14.280433000	17.748200000	11.872512000
H	14.367882000	17.062860000	12.717649000
C	14.161047000	19.124956000	12.090108000
H	14.148010000	19.518942000	13.108242000
C	14.053524000	19.999568000	11.008984000
H	13.963096000	21.075429000	11.166524000
C	10.296720000	22.163928000	8.114616000
C	8.945397000	21.902355000	8.402056000

H	8.662846000	21.604916000	9.415269000
C	7.966918000	21.986179000	7.409172000
H	6.922640000	21.780797000	7.655250000
C	8.325749000	22.312789000	6.097951000
H	7.565540000	22.364701000	5.316397000
C	9.669572000	22.548252000	5.792994000
H	9.963060000	22.784539000	4.767926000
C	10.642455000	22.472232000	6.791024000
H	11.698104000	22.624382000	6.554376000
C	11.421136000	23.468397000	10.386318000
C	10.207465000	24.153666000	10.573298000
H	9.312682000	23.824314000	10.043746000
C	10.131285000	25.271225000	11.406992000
H	9.177304000	25.790798000	11.522903000
C	11.264381000	25.733313000	12.082323000
H	11.202936000	26.607541000	12.732837000
C	12.479017000	25.064343000	11.902600000
H	13.379592000	25.413207000	12.413314000
C	12.555424000	23.951287000	11.065694000
H	13.508839000	23.445136000	10.903041000
P	17.090838000	21.194852000	8.873461000
P	19.074512000	18.783030000	9.603945000
C	17.512076000	19.711927000	9.949705000
H	16.637239000	19.058168000	9.833999000
H	17.539612000	20.030610000	10.999925000
C	18.590483000	21.530145000	7.861188000
C	19.857369000	21.767239000	8.410255000
H	19.992477000	21.795958000	9.491222000
C	20.959674000	21.968447000	7.574711000

H	21.944698000	22.145590000	8.010219000
C	20.800957000	21.939437000	6.187660000
H	21.663635000	22.091523000	5.536138000
C	19.534384000	21.718600000	5.637006000
H	19.405521000	21.694683000	4.553486000
C	18.435405000	21.517212000	6.470854000
H	17.427188000	21.336307000	6.088340000
C	17.144543000	22.573754000	10.120109000
C	16.245668000	23.627480000	9.908434000
H	15.572310000	23.563391000	9.051847000
C	16.189336000	24.703463000	10.796123000
H	15.471462000	25.507171000	10.624740000
C	17.030601000	24.737421000	11.911185000
H	16.980988000	25.573579000	12.610939000
C	17.927582000	23.689328000	12.134752000
H	18.582730000	23.705863000	13.007925000
C	17.983034000	22.614300000	11.244221000
H	18.686212000	21.801590000	11.438165000
C	18.884931000	18.340086000	7.824480000
C	20.036883000	18.326393000	7.026568000
H	20.993835000	18.605870000	7.470996000
C	19.960822000	17.994718000	5.671984000
H	20.864035000	17.998343000	5.059670000
C	18.724858000	17.685367000	5.100869000
H	18.658647000	17.438763000	4.039739000
C	17.568870000	17.706412000	5.886436000
H	16.596676000	17.485625000	5.443533000
C	17.646280000	18.030077000	7.241172000
H	16.734285000	18.063993000	7.835777000

C	18.683784000	17.142310000	10.360977000
C	19.350934000	15.996136000	9.892120000
H	20.016447000	16.078337000	9.030436000
C	19.153815000	14.755021000	10.494581000
H	19.672364000	13.877122000	10.104617000
C	18.288447000	14.630678000	11.586554000
H	18.129046000	13.658498000	12.055321000
C	17.622576000	15.760958000	12.061784000
H	16.929908000	15.678141000	12.901298000
C	17.818523000	17.004959000	11.457072000
H	17.264520000	17.866098000	11.830220000

Atomic coordinates of (CH(CHPPh$_2$)$_2$ (**20c**) optimized (PBE-D3(BJ)/def2-TZVPP) in the ground state S_0 in the gas phase.

P	14.334905000	19.824083000	8.072039000
P	12.060620000	20.912042000	10.144503000
C	14.548209000	20.782990000	6.621921000
C	13.791872000	20.320388000	9.821233000
H	14.528855000	21.031070000	10.204009000
H	13.895197000	19.403594000	10.422496000
C	12.523329000	19.265435000	7.625856000
C	12.006746000	18.031068000	8.055063000
H	12.662344000	17.325366000	8.568456000
C	10.670697000	17.680226000	7.848257000
H	10.303991000	16.712210000	8.197656000
C	9.803840000	18.563748000	7.201324000
H	8.757841000	18.294878000	7.041350000
C	10.291987000	19.797708000	6.767981000

H	9.625680000	20.507790000	6.272743000
C	11.630395000	20.138429000	6.980386000
H	11.981283000	21.121791000	6.665608000
C	14.980344000	18.095009000	8.123230000
C	15.047067000	17.380583000	6.917909000
H	14.651167000	17.842261000	6.011265000
C	15.613327000	16.106120000	6.874329000
H	15.673398000	15.572076000	5.923565000
C	16.094882000	15.509789000	8.043266000
H	16.544144000	14.516107000	8.010665000
C	16.006933000	16.203715000	9.252543000
H	16.390780000	15.754763000	10.170619000
C	15.460351000	17.486773000	9.290278000
H	15.442511000	18.028194000	10.236771000
C	12.032403000	22.535591000	9.280821000
C	10.779462000	23.146967000	9.108675000
H	9.887114000	22.639954000	9.483141000
C	10.665459000	24.374640000	8.456092000
H	9.684098000	24.836762000	8.332750000
C	11.807562000	25.000674000	7.946176000
H	11.722329000	25.954504000	7.422233000
C	13.056493000	24.395347000	8.096844000
H	13.950995000	24.867750000	7.689728000
C	13.170046000	23.171738000	8.762765000
H	14.152511000	22.709255000	8.861247000
C	12.317319000	21.466870000	11.893630000
C	12.461881000	22.803938000	12.296000000
H	12.468934000	23.593027000	11.543420000
C	12.603974000	23.132751000	13.646679000

H	12.715404000	24.179504000	13.937076000
C	12.616231000	22.132605000	14.621206000
H	12.734504000	22.390443000	15.674849000
C	12.479501000	20.796270000	14.232612000
H	12.494683000	20.003436000	14.982653000
C	12.321052000	20.471289000	12.886887000
H	12.200660000	19.425363000	12.594213000
P	16.131568000	21.368671000	6.939148000
P	17.661152000	20.549826000	9.426637000
C	16.217668000	20.578623000	8.447937000
C	17.384320000	20.863170000	5.695369000
C	18.694944000	21.364754000	5.745695000
H	18.942386000	22.152661000	6.459802000
C	19.684816000	20.840090000	4.915949000
H	20.704549000	21.224289000	4.978462000
C	19.377761000	19.814870000	4.015306000
H	20.155104000	19.401463000	3.370559000
C	18.072636000	19.319871000	3.949649000
H	17.825773000	18.520313000	3.248294000
C	17.082467000	19.841097000	4.784038000
H	16.062621000	19.451967000	4.762097000
C	16.331208000	23.199530000	6.895798000
C	16.065146000	23.928378000	5.727156000
H	15.791044000	23.398441000	4.812386000
C	16.138306000	25.321927000	5.729154000
H	15.911140000	25.881356000	4.819676000
C	16.498632000	26.002095000	6.898287000
H	16.556565000	27.091962000	6.900575000
C	16.784045000	25.281584000	8.060432000

H	17.072705000	25.806999000	8.972535000
C	16.698148000	23.886082000	8.059745000
H	16.916589000	23.303858000	8.957509000
C	18.654126000	19.017320000	9.129719000
C	19.758588000	18.695197000	9.941832000
H	19.944034000	19.273378000	10.850523000
C	20.620939000	17.652905000	9.601879000
H	21.464667000	17.413066000	10.253377000
C	20.413588000	16.920960000	8.426648000
H	21.092003000	16.109596000	8.156367000
C	19.332507000	17.245390000	7.602673000
H	19.151993000	16.683530000	6.684018000
C	18.464970000	18.280137000	7.951320000
H	17.615730000	18.528653000	7.314994000
C	17.075160000	20.325003000	11.161401000
C	16.461202000	21.433096000	11.776728000
H	16.363088000	22.362432000	11.209882000
C	15.954091000	21.358641000	13.071422000
H	15.449314000	22.221435000	13.507876000
C	16.066539000	20.170825000	13.802174000
H	15.658509000	20.107939000	14.811885000
C	16.687342000	19.066477000	13.215810000
H	16.773744000	18.129098000	13.770201000
C	17.181035000	19.140753000	11.909860000
H	17.646707000	18.263501000	11.459263000
H	13.867144000	20.944844000	5.794871000
